# Exploring the genome of Arctic *Psychrobacter* sp. DAB_AL32B and construction of novel *Psychrobacter*-specific cloning vectors of an increased carrying capacity

**DOI:** 10.1007/s00203-018-1595-y

**Published:** 2018-11-17

**Authors:** Anna Ciok, Lukasz Dziewit

**Affiliations:** Department of Bacterial Genetics, Faculty of Biology, Institute of Microbiology, University of Warsaw, Miecznikowa 1, 02-096 Warsaw, Poland

**Keywords:** *Psychrobacter* sp. DAB_AL32B, *Psychrobacter*-specific vector, Draft genome sequence, Stress adaptation

## Abstract

Cold-active bacteria are currently of great interest in biotechnology, and their genomic and physiological features have been extensively studied. One of the model psychrotolerant bacteria are *Psychrobacter* spp. Analysis of Arctic psychrophilic *Psychrobacter* sp. DAB_AL32B genome content provided an insight into its overall stress response, and genes conferring protection against various life-limiting factors (i.e., low temperature, increased ultraviolet radiation, oxidative stress and osmotic pressure) were recognized and described. Moreover, it was revealed that the strain carries a large plasmid pP32BP2. Its replication system was used for the construction of two novel shuttle vectors (pPS-NR—*Psychrobacter*-*Escherichia coli*-specific plasmid and pPS-BR—*Psychrobacter*-various *Proteobacteria*-specific plasmid) of an increased carrying capacity, which may be used for genetic engineering of *Psychrobacter* spp.

**Electronic supplementary material:**

The online version of this article (10.1007/s00203-018-1595-y) contains supplementary material, which is available to authorized users.

## Introduction

*Psychrobacter* spp. belong to the *Moraxellaceae* family (*Gammaproteobacteria*). Bacteria of this genus are frequently isolated from various cold environments, including seawater, ice, permafrost and Arctic and Antarctic ornithogenic soils. Some strains (e.g., *P. pulmonis* and *P. phenylpyruvicus*) are considered to be opportunistic pathogens, as they are occasionally isolated from human patients, as well as from infected animals (Bowman et al. 1996; Bowman 2006; Ortiz-Alcantara et al. 2016).

*Psychrobacter* strains grow in temperatures between − 10 and 38 °C, and the majority of strains are psychrotolerants. Usually, *Psychrobacter* spp. are also recognized as halotolerants, since they are able to grow in the presence of 6.5% saline and above. Their ability to thrive in permanently cold and high-salt environments distinguish *Psychrobacter* spp. from their closest relatives, i.e., *Acinetobacter* and *Moraxella* (Maruyama et al. 2000; Garrity 2005; Bowman 2006).

According to the NCBI genome browser (http://ncbi.nlm.nih.gov/genome/browse), 77 genomes (including 65 drafts) and an additional 65 plasmid sequences of *Psychrobacter* spp. are available (22nd June 2018). Analysis of the *Psychrobacter* genomes revealed the presence of various adaptation mechanisms allowing their survival in extremely cold environments. Hence, psychrophilic *Psychrobacter* strains are model, cold-active bacteria useful for studying bacterial adaptation to extreme conditions (Bakermans et al. 2007; Zheng et al. 2007; Ayala-del-Rio et al. 2010; Moghadam et al. 2016; Dias et al. 2018). Moreover, they constitute a biotechnologically valuable source of cold-active enzymes, e.g., carbonic anhydrase and lipases (Zhang et al. 2007; Parra et al. 2008; Li et al. 2016; Lasa and Romalde 2017). It is also worth mentioning that, *Psychrobacter* spp. were reported to exhibit resistances to various heavy-metal ions, including arsenate, arsenite, copper, tellurite, and chromate (Dziewit et al. 2013; Arenas et al. 2014; Munoz-Villagran et al. 2018). Heavy-metal resistance may be beneficial in the light of a potential application of *Psychrobacter* strains in various bioremediation technologies.

Considering the application of *Psychrobacter* spp. or their enzymes in various biotechnologies, it was found that these bacteria are usually quite easy to cultivate under laboratory conditions, as they grow rapidly at 20–25 °C and undergo genetic recombination by conjugation and electroporation. There are reports concerning genetic modifications applying transposon mutagenesis and allele exchange carried out for *Psychrobacter* spp. (Bakermans et al. 2009; Jeong et al. 2013). Therefore, it is possible to use bacteria of this genus as biological factories, e.g., for synthesis of cold-adapted enzymes, which cannot be produced in commonly found mesophilic strains. However, there is an urgent need for novel vectors specific for *Psychrobacter* spp., which can be used for cloning of exogenous DNA and expression of proteins in that host.

It was previously reported that plasmids carrying ColE1- and p15a-type replication systems were stably maintained in *P. arcticus* 274-3 (Bakermans et al. 2009). Nonetheless, in our experiments with several cold-active *Psychrobacter* strains, we were unable to introduce (neither by electroporation nor triparental mating) a pABW1 vector containing a ColE1-type replication system. Therefore, the broad host range vector pBBR1 MCS-2 with pBBR1-type replication system was tested. Surprisingly, this replication system was also found to be inactive in tested *Psychrobacter* spp. These negative results encouraged us to construct novel, shuttle *Psychrobacter*–*Escherichia coli*-specific vectors.

So far, five such vectors have been made available. Four shuttle vectors (pPCV1-4), based on the pBGS18 (carrying *E. coli*-specific replication system originating from the pMB1 plasmid, i.e., high copy number replicon) or pWSK29 (carrying *E. coli*-specific replication system originating from the pSC101 plasmid, i.e., low copy-number replicon) plasmids, and carrying replication systems of the pP43BP3 and pP43BP4 plasmids originating from psychrophilic *Psychrobacter* sp. DAB_AL43B, were reported (Lasek et al. 2017). Additionally, a pUC-ORIT/rep vector, based on *E. coli*-specific pUC18 vector and pTAUp replication system originating from 1.9-kb plasmid of Antarctic *Psychrobacter* sp. TA144, was constructed (Tutino et al. 2000). It is noteworthy that all above-mentioned vectors were constructed applying replication systems of relatively small replicons (1.9–6.45 kb). Cloning of large fragments using vectors containing replication systems of small-size plasmids may result in an insert instability, as was shown for, e.g., vector pSUP106 with a replication system derived from 8.7-kb plasmid RSF1010 (Priefer et al. 1985). Therefore, we recognized the need for *Psychrobacter*-specific vectors based on replication systems of large plasmids, and therefore potentially having an increased carrying capacity.

In this study, the draft genome sequence of *Psychrobacter* sp. DAB_AL32B isolated from the Hornsund, Spitsbergen (Arctic) was obtained. The genome analyses provided brief insights into the stress adaptation mechanisms of the strain. Moreover, we found that the strain carries a large, approximately 60-kb, plasmid. Although this plasmid was not fully assembled during genome drafting, its replication system was used for the construction of two novel *Psychrobacter*-specific vectors that are suitable for cloning of large DNA fragments.

## Materials and methods

### Bacterial strains, plasmids and culture conditions

The bacterial strains and plasmids used in this study are listed in Table 1. All strains were grown on lysogeny broth (LB) medium (Sambrook and Russell 2001) at 22 °C (*Psychrobacter* spp.) or 37 °C (*Escherichia coli* DH5α). The medium was solidified by the addition of 1.5% (w/v) agar. Where necessary, the media were supplemented with X-gal, IPTG and antibiotics: kanamycin (20 µg/ml for *Psychrobacter* spp. or 50 µg/ml for *E. coli*) and rifampin (50 µg/ml).

**Table 1:** Strains and plasmids used in this study

Strain or plasmid	Characteristics	Reference or source
*E. coli* DH5α	*F*^−^; Φ80*lacZΔ*M15 Δ(*lac*ZYA-*arg*F) U169 *rec*A1 *end*A1 *hsd*R17 (r_K_^−^, m_K_^+^) *pho*A *sup*E44 *λ*^−^*thi*-1 *gyr*A96 *rel*A1 *λ*^−^	Hanahan (1983)
*Psychrobacter* sp. DAB_AL12R	Rif^r^, recipient strain	Lasek et al. (2017)
*Psychrobacter* sp. DAB_AL32B	Wild type, Arctic strain	Dziewit et al. (2013)
*Psychrobacter* sp. DAB_AL43BR	Rif^r^, recipient strain	Lasek et al. (2017)
pABW1	Km^r^; 4.5 kb; *ori* pMB1; *oriT* RK2; *lacZα*; MCS	Bartosik et al. (1997)
pBBR1 MCS-2	Km^r^; 5.1 kb; *ori* pBBR1; Mob^+^; *oriT* RK2; *lacZα*; MCS	Kovach et al. (1994)
pPS-BR	Km^r^; 6.9 kb; pBBR1 MCS-2 derivative carrying PCR-amplified replication system of the pP32BP2 plasmid cloned within PfoI site	This study
pPS-NR	Km^r^; 6.2 kb; pABW1 derivative carrying PCR-amplified replication system of the pP32BP2 plasmid cloned within the PfoI site	This study
pRK2013	Km^r^; 48.0 kb; helper plasmid carrying genes for conjugal transfer of RK2	Ditta et al. (1980)

### DNA sequencing

The CTAB/lysozyme method was used for isolation of genomic DNA (Sambrook and Russell 2001). An Illumina TruSeq library was constructed following the manufacturer’s instructions. Sequencing was performed on an Illumina MiSeq instrument using the v3 chemistry kit. Sequence reads were filtered for quality and assembled using Newbler version 3.0 software with default settings (Roche, Basel, Switzerland), as it generated longest contigs (compared to other assembling tools). PCR products were cloned into the pABW1 and pBBR1 MCS-2 vectors and the resulting plasmids were used for DNA sequencing applying a dye terminator sequencing kit and an automated sequencer (ABI 377 PerkinElmer) (Applied Biosystems, Waltham, USA). Primer walking was employed to obtain the complete nucleotide sequence of the cloned PCR products.

All sequencing was performed in the DNA Sequencing and Oligonucleotide Synthesis Laboratory at the Institute of Biochemistry and Biophysics, Polish Academy of Sciences (Warsaw, Poland).

### DNA manipulations and introduction of plasmid DNA into bacterial cells

Plasmid DNA was isolated using a GeneMATRIX Plasmid Miniprep DNA Purification Kit (EURx, Gdansk, Poland) and alkaline lysis method (Birnboim and Doly 1979). Standard DNA manipulations were performed according to Sambrook and Russell (2001). PCR was performed in a Mastercycler (Eppendorf, Hamburg, Germany) using KAPA HiFi PCR Kit (KAPA polymerase with supplied components) (KAPA Biosystems, Cape Town, South Africa) and primer pair (the restriction enzyme sites are underlined) 32B_REPF—5′-ATACCGGTGCGAACCACTGTGAGTATTG-3′ and 32B_REPR—5′-ATACCGGTTTAATTCTATCGCCCGCCTG-3′. To obtain the PCR product, the following conditions were applied: 35 cycles (denaturation at 98 °C for 20 s, annealing at 61.4 °C for 60 s, extension at 72 °C for 30 s per 1 kb) preceded by 3-min denaturation at 95 °C and followed by 2-min extension at 72 °C.

The shuttle vectors were introduced into *E. coli* DH5α by transformation (Kushner 1978) and into *Psychrobacter* spp. via triparental mating. The triparental mating was conducted as follows: recipient Rif^r^-*Psychrobacter* strains were cultivated on LB agar plates for 1 day at 22 °C, then donor and helper strains were spread on a plate with pre-grown recipient strain and cultivated for 2 days at 22 °C. After 2 days of incubation, the bacteria were washed off the plate and suitable dilutions were plated on selective medium containing rifampin and kanamycin to select transconjugants carrying introduced Km^r^-vector. The plates were cultivated for the next 3 days at 22 °C.

### Plasmid stability assay

The pPS-NR plasmid was introduced into the DAB_AL12R and DAB_AL43BR strains via triparental mating. The presence of pPS-NR within analyzed strains was confirmed via alkaline lysis. Segregational stability of the pPS-NR vector in the DAB_AL12R and DAB_AL43BR strains was tested by replica plating, following growth under non-selective conditions for 30 generations, as described previously (Dziewit et al. 2007; Romaniuk et al. 2017).

### Bioinformatics

The draft genome was automatically annotated using the NCBI Prokaryotic Genome Annotation Pipeline. Similarity searches were performed using the BLAST programs (Altschul et al. 1997). Secondary structures of proteins were predicted using InterPro (Jones et al. 2014) and Motif Scan (Pagni et al. 2007). For plasmids’ assembly from the draft genome, the plasmidSPAdes v. 3.12.0 tool (Antipov et al. 2016) was used. All programs were used with default settings.

Genome completeness was assessed by the presence/absence of bacterial orthologs according to the OrthoDB database using BUSCO (Simao et al. 2015). Genome contamination was determined using Taxoblast v. 1.1 (Dittami and Corre 2017) followed by manual evaluation of results. All contigs representing analyzed genome were splitted into sequences of 1000 bp length. Each DNA fragment was individually searched for homologous sequence in GenBank nr database (e value cut off 0.01) excluding genome of the analyzed DAB_AL32B strain. The *Psychrobacter* (taxon ID 497) was specified as taxon separating from other bacterial taxa.

PYANI (Pritchard et al. 2016) with ANIb method was used to calculate ANI values between the DAB_AL32B strain and each of 76 genomes of *Psychrobacter* spp.

### 16S rRNA gene phylogenetic analysis

Phylogenetic analysis was performed based on the comparison of partial 16S rRNA gene sequences of the DAB_AL32B strain, DAB_AL43B strain and strains representing 48 *Psychrobacter* species described to date. Sequences were aligned using the RDP Aligner tool (structure-aware multiple sequence aligner) available at the Ribosomal Database Project (RDP) website (Nawrocki and Eddy 2007). Phylogenetic tree was built using Tree Builder (with default settings) available at the RDP website. The 16S rRNA gene sequences used for phylogenetic analysis were collected from GenBank (NCBI). The GenBank sequences used for this analysis are as follows: *P. adeliensis* DSM 15333^T^ (HE654007.1), *P. aestuarii* SC35 (EU939718.1), *P. alimentarius* JG-100 (AY513645.1), *P. allis* E2 (JX122558.1), *P. aquaticus* CMS 56 (NR_042206.1), *P. aquimaris* SW-210 (AY722804.1), *P. arcticus* 273-4 (NR_075054.1), *P. arenosus* R7^T^ (AJ609273.1), *P. celer* SW-238 (NR_043225.1), *P. ciconiae* 176/10 (KM486054.1), *P. cryohalolentis* K5 (NR_075055.1), *P. faecalis* DSM 14664 (NR_118025.1), *P. fjordensis* BSw21516B (GQ358940.1), *P. fozii* NF23 (NR_025531.1), *P. frigidicola* DSM 12411 (NR_042222.1), *P. fulvigenes* KC 40 (NR_041688.1), *P. glacialis* DD43 (AJ539102.1), *P. glaciei* BIc20019 (NR_148850.1), *P. glacincola* DSM 12194 (NR_042076.1), *P. halophilus* DD2 (AJ539103.1), *P. immobilis* ATCC 43116 (NR_118808.1), *P. jeotgali* YKJ-103 (NR_025205.1), *P. luti* NF11 (NR_025532.1), *P. lutiphocae* IMMIB L-1110 (NR_044602.1), *P. marincola* KMM 277 (NR_025458.1), *P. maritimus* Pi2-20 (NR_027225.1), *P. meningitidis* SBA4 (KR091838.1), *P. muriicola* 2pS (NR_114669.1), *P. namhaensis* SW-242 (AY722805.1), *P. nivimaris* 88/2–7 (AJ313425.1), *P. oceani* 4k5 (AB910522.1), *P. okhotskensis* MD17 (NR_024806.1), *P. pacificensis* NBRC 103191 (NR_114238.1), *P. pasteuri* CIP110853 (KY292376.1), *P. phenylpyruvicus* ATCC 23333 (NR_118815.1), *P. piechaudii* CIP110854 (KY292375.1), *P. piscatorii* T-3-2 (NR_112807.1), *P. piscidermidis* 45 (FJ613616.1), *P. pocilloporae* S6-60 (KT444699.2), *P. proteolyticus* HAMBI 2948 (LT899990.1), *P. psychrophilus* BBDP29 (DQ337513.1), *P. pulmonis* CCUG 46240 (NR_118026.1), *P. salsus* DD 48 (NR_042166.1), *P. sanguinis* 13983 (HM212668.1), *P. submarinus* KMM 225 (AJ309940.1), *P. urativorans* DSM 14009^T^ (AJ609555.1), *P. vallis* CMS 39 (AJ584832.1), *P. cibarius* JG-219 (AY639871.1), *Psychrobacter* sp. DAB_AL32B (JF714884.1) and *Psychrobacter* sp. DAB_AL43B (JF714885.1).

### Nucleotide sequence accession number

The *Psychrobacter* sp. DAB_AL32B draft genome sequence has been deposited in the GenBank (NCBI) database under accession numbers NEXU01000001-NEXU01000218 and the sequences of vectors constructed in this study under accession numbers MH539767 (pPS-BR) and MH539768 (pPS-NR).

## Results and discussion

### General features of the *Psychrobacter* sp. DAB_AL32B genome

The estimated size of the DAB_AL32B genome is 3,211,529 bp, which is similar to the size of other *Psychrobacter* spp. genomes available in the NCBI database (the average length of the *Psychrobacter* genome is 3.07 Mb). An assembly of the DAB_AL32B draft genome resulted in 37 scaffolds composed of 218 contigs. The average GC content is 41.9%, which is typical for *Psychrobacter* spp. (the average value is 43.26%). An automatic annotation of the DAB_AL32B genome performed applying the NCBI Prokaryotic Genome Annotation Pipeline resulted in 2,799 predicted genes with an average length of 973.43 bp, which covers about 80.08% of the genome. Additionally, 41 tRNA genes were identified (Table S1, Supplementary materials).

To estimate the completeness of the obtained draft genome, a BUSCO analysis, using *Gammaproteobacteria* BUSCO set (containing 452 BUSCO groups), was performed. The gene set predicted within the DAB_AL32B genome contains 94.3% of genes present in an applied BUSCO set (92.5% complete and 1.8% fragmented genes), which suggests that the genome assembly is complete or very close to completeness.

To examine if obtained draft genome is contaminated with other genomic sequences, an analysis using Taxoblast was performed (Table S2, Supplementary material). This analysis revealed that 30 out of 218 contigs were classified as possible contaminants or did not exhibit significant homology to sequences available in the GenBank database. These contigs were manually examined. Putative prophage was found within contig00038. Seven contigs (contig00009, contig00020, contig00096, contig00102, contig00104, contig00122, and contig00128) mostly exhibited homology to *Psychrobacter* genomes, with only short fragments homologous to genomes of phylogenetically closely related *Moraxella* and *Acinetobacter*. Other two contigs (contig00048 and contig00081) exhibit similarity to *Psychrobacter* genomes in majority of their fragments, while the remaining fragments are either homologous to *Acinetobacter, Moraxella*, and other unrelated bacteria, or not homologous to any sequence in the nr database. However, BLASTx search revealed significant similarity to proteins encoded within most of these fragments to *Psychrobacter* and *Acinetobacter* proteins. For three other contigs (contig00140, contig00162 and contig00205) the best BLAST hits were sequences from either *Acinetobacter* or *Moraxella*; however, they showed comparable similarity to *Psychrobacter* genomes. One contig (contig00082) was mistakenly automatically classified as a contaminant, because of a non-significant hit to zebrafish genome in low-complexity region. As a result of above analysis, these 15 contigs were recategorized as non-contaminant sequences. Seven other contigs (contig00002, contig00150, contig00177, contig00184, contig00193, contig00199, and contig00218) exhibited no significant homology to sequences available in the GenBank database (total length 2,557 bp, which comprises 0.0008% of the draft genome), therefore it is unclear if these are contaminants or not. Finally, only nine contigs (total length 4,558 bp, 0.0014% of the draft genome) (contig00134, contig00143, contig00146, contig00174, contig00181, contig00195, contig00196, contig00197, and contig00210) may be considered as potential contaminants, as they do not show similarities to neither *Psychrobacter* sequences nor closely related bacteria.

### Phylogenetic analysis *of Psychrobacter* sp. DAB_AL32B

**Fig. 1:**
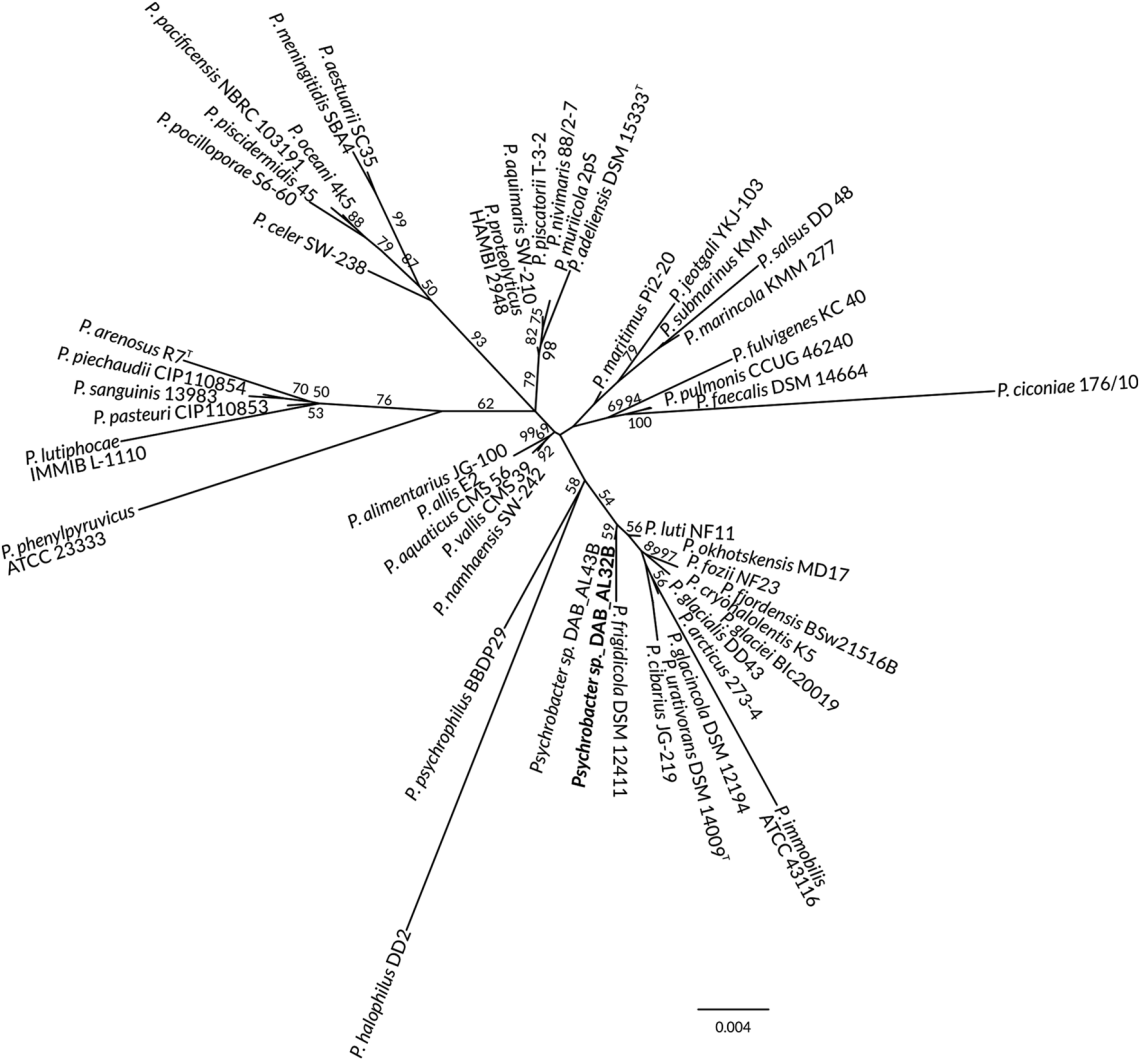
Phylogenetic tree for 16S rDNA sequences of *Psychrobacter* spp. The DAB_AL32B strain is denoted by the bold text. The statistical support for the internal nodes was determined by 1000 bootstrap replicates and values of > 50% are shown. The scale bar represents 0.004 substitutions per nucleotide position. The tree was not rooted

Previous phylogenetic analysis (Dziewit et al. 2013), based on the comparison of partial 16S rDNA sequences, showed that the DAB_AL32B strain is closely related to *Psychrobacter frigidicola* DSM 12411. As several new *Psychrobacter* species have been described since the last analysis, we performed another analysis with dataset extended on 14 new *Psychrobacter*-type strains (Fig. 1). Same as previously, the DAB_AL32B strain was clustered with *P. frigidicola* DSM 12411.

For more precise classification of DAB_AL32B at the species level, the ANI analysis, based on whole-genome comparison between various *Psychrobacter* strains, was performed. The DAB_AL32B genome was compared to each of 76 *Psychrobacter* genomes available (18th July 2018) in NCBI database (Table S3, Supplementary materials). Unfortunately, the genome of *P. frigidicola* DSM 12411 or some other representative of this species, is not publicly available. The phylogenomic analysis revealed that the DAB_AL32B strain is most related with *Psychrobacter* sp. DAB_AL43B (ANI value 95.672). However, to predict if these strains may be considered as representatives of novel *Psychrobacter* species, the calculation of the ANI value with *P. frigidicola* is needed.

### Genes involved in the stress response of the DAB_AL32B strain

Various adaptive mechanisms enable survival of psychrophilic bacteria in Arctic. Identification and analysis of genes encoding various stress response proteins of psychrophiles is important for studying their biology and adaptation, as well as for proper recognition of their biotechnological potential (D’Amico et al. 2006; Casanueva et al. 2010; De Maayer et al. 2014; Santiago et al. 2016). In its natural environment, *Psychrobacter* sp. DAB_AL32B had to cope not only with low temperatures, but also increased ultraviolet (UV) radiation, osmotic pressure and oxidative stress. The genes involved in the response to oxidative, osmotic, and cold shock encoded within the DAB_AL32B genome are listed in Table 2.

**Table 2:** Proteins with functions associated with adaptation to hostile polar environment found within the DAB_AL32B genome

Predicted protein function	Enzyme commission (EC) or transporter classification (TC) numbers	GenBank accession number
*Protection against oxidative stress*		
Catalase	EC 1.11.1.6	OXL27137, OXL24953, OXL18754
Peroxidase	EC 1.11.1.7	OXL25173
Superoxide dismutase	EC 1.15.1.1	OXL25901
Glutaredoxin	N/A^a^	OXL28806, OXL26709, OXL18108
Various enzymes involved in glutathione metabolism		
γ-Glutamyltransferase	EC 2.3.2.2	OXL26439
Hydroxyacylglutathione hydrolase	EC 3.1.2.6	OXL25200
Glutathione synthase	EC 6.3.2.3	OXL27171
Glutamate–cysteine ligase	EC 6.3.2.2	OXL20295
Lactoylglutathione lyase	EC 4.4.1.5	OXL24272, OXL19693
Gluthatione S-transferase	EC 2.5.1.18	OXL28870, OXL28681, OXL24930, OXL23123, OXL20411, OXL18525, OXL24077
Rubredoxin	N/A	OXL26516
Peroxiredoxin	N/A	OXL23338, OXL20410
Dps-like DNA-binding protein	N/A	OXL27674, OXL23702
*Protection against cold-shock stress*		
Cold-shock proteins (CspA, CspC, CspE)	N/A	OXL21528, OXL25544, OXL18230
*Protection against osmotic stress*		
Choline-glycine betaine transporter, BCCT family	N/A	OXL26189, OXL23897, OXL23830, OXL22979, OXL21502
ABC-type proline/glycine betaine transporter	TC 3.A.1.12.1	OXL22993, OXL22990
Choline dehydrogenase	EC 1.1.99.1	OXL28998, OXL23913
Betaine aldehyde dehydrogenase	EC 1.2.1.8	OXL23898
Choline sulfatase	EC 3.1.6.6	OXL27197

^a^*N/A* not assigned

Analysis of the DAB_AL32B genome showed that the most represented group of genes encoding stress response proteins is linked with oxidative stress, including protection against reactive oxygen species (ROS) formed in the cell. Increased ROS formation in cells may occur as a result of (i) depletion of the UV-protective ozone layer in the Arctic region (He and Hader 2002; Dugo et al. 2012) and (ii) permanent low temperatures, which increases the solubility of oxygen in water (Casanueva et al. 2010; Baez and Shiloach 2014). Cells exposed to elevated oxygen concentrations accumulate ROS, formed as byproducts of aerobic metabolism. Oxidants are highly reactive molecules and trigger damage in cellular components. They cause the oxidation of amino acids, which leads to protein fragmentation, the formation of aggregates and proteolysis (Cabiscol et al. 2000). DNA modifications caused by ROS include single- and double-strand breaks, bases modifications and cross-linking with proteins, leading to the mutation and rearrangement of DNA (Cabiscol et al. 2000; Jena 2012). Moreover, as a result of oxidation (induced by ROS and facilitated by Fe^2+^ ions), lipids cross-linking with proteins and disturbances to membrane structure affecting its fluidity are observed (Cabiscol et al. 2000; Repetto 2012). Bacteria may control ROS formation and protect themselves from oxidative stress applying various mechanisms, including scavenging ROS, maintaining a strong reducing environment in the cytosol and direct protection of vulnerable molecules (Cabiscol et al. 2000; Imlay 2013).

Within the DAB_AL32B genome, 11 genes encoding enzymes directly responsible for a diminishing amount of toxic ROS were found: catalases (3 genes), peroxidase (1), superoxide dismutase (1), peroxiredoxin (2), rubredoxin (1) and glutaredoxins (3) (Table 2). Additionally, 13 genes encoding enzymes involved in glutathione metabolism are present within the genome. Glutathione is a low-molecular weight thiol, protecting the cell from oxidative stress induced by peroxides (Masip et al. 2006).

Oxygen toxicity is increased by an excess of an intracellular iron in its reduced form (Fe^2+^). Hydroxyl radicals are then produced via Fenton reaction (Touati 2000). Therefore, it is essential to maintain a low and safe level of intracellular concentration of these ions. Proteins belonging to the ferritin superfamily are responsible for iron storage in its nontoxic oxidized form (Castruita et al. 2006; Arosio et al. 2009; Calhoun and Kwon 2011). Within the DAB_AL32B genome, two genes encoding Dps-like DNA-binding proteins were found (Table 2). These proteins contain both DNA-binding and ferroxidase domains and their proposed biological function is DNA protection from oxidation (via sequestration and oxidation of Fe^2+^ ions) and formation of very stable complexes with DNA (Castruita et al. 2006).

The annual temperature in the Spitsbergen ranges between − 35.9 and 13.5 °C (Nowosielski 2004; Przybylak and Arazny 2006). Therefore, bacteria of the genus *Psychrobacter* inhabiting this region have to cope with permanent cold. Low temperatures significantly influence cell functioning by altering protein conformation (and, in consequence, its activity), decreasing gene expression and membrane fluidity (Zecchinon et al. 2001; Chattopadhyay 2006; D’Amico et al. 2006). *Psychrobacter* sp. DAB_AL32B possesses genes encoding three proteins, i.e., CspA, CspC, and CspE, belonging to the family of cold-shock proteins (Table 2). Low temperatures induce stabilisation of secondary structures in RNA, which suppresses gene expression by blocking RNA polymerase and ribosomes (Phadtare and Severinov 2010). Csp proteins act as chaperones and resolve secondary structures in RNA, which prevents premature transcription termination and blocking of translation (Phadtare et al. 1999; Kaufman-Szymczyk et al. 2009; Phadtare and Severinov 2010; Song et al. 2012; Keto-Timonen et al. 2016).

To cope with the permanent cold, but also with the osmotic stress, bacteria intake compatible solutes (e.g., proline, choline and glycine betaine) from the environment or synthesise them in elevated quantities. Compatible solutes increase the stability of proteins and cell membranes without interfering with cellular function (Ziegler et al. 2010). The DAB_AL32B strain has five genes encoding osmolyte transporters belonging to the BCCT family (betaine/carnitine/choline transporter) and two genes encoding subunits of the ABC-type proline/glycine betaine transporter. In addition, four enzymes, involved in glycine betaine and choline synthesis, were found (Table 2).

### Insight into plasmidome of *Psychrobacter* sp. DAB_AL32B

As reported previously, the DAB_AL32B strain harbours small (4.6-kb), cryptic plasmid pP32BP1 (Dziewit et al. 2013). Moreover, based on the alkaline lysis results, we found that the strain most probably carries also a large, approximately 60-kb, plasmid, named pP32BP2. It was also partially confirmed by the genome drafting. Using plasmidSPAdes, three contigs, i.e.: contig00102 (GenBank: NEXU01000102), contig00103 (GenBank: NEXU01000103), and contig00104 (GenBank: NEXU01000104) were predicted as potential fragments of the pP32BP2 plasmid. Obtaining the complete genomic sequence of this plasmid is the aim of our further studies. Within the contig00104, genes encoding proteins involved in plasmid replication and partitioning were found.

**Fig. 2:**
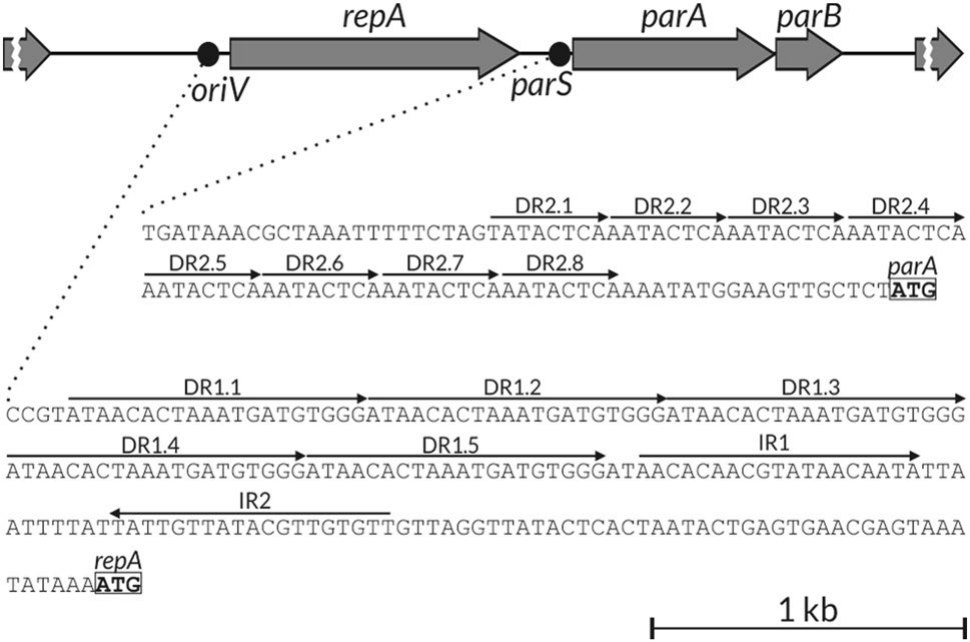
Genetic organization of the maintenance module of the pP32BP2 plasmid. Grey arrows indicate genes and their transcriptional orientation, dots indicate location of *oriV* and *parS* sites, thin black arrows (above the DNA sequence) indicate location of direct and/or inverted repeats within the *oriV* and *parS* sites. The start codons of the *parA* and *repA* genes are in frames. *DR* direct repeat, *IR* inverted repeat, *oriV* origin of plasmid replication, *parA* gene encoding partitioning protein A, *parB* gene encoding partitioning protein B, *parS* partitioning centromer-like site, *repA* gene encoding replication protein

The replication initiation protein RepA (GenBank: OXL18943) of pP32BP2 shows 76% identity with RepB protein (GenBank: AFD62164) of pP62BP1 of *Psychrobacter* sp. DAB_AL62B (Lasek et al. 2012). Analysis of the RepA sequence revealed the existence of a leucine zipper motif (LVAKSNDLIVASYELTRNEQRL, residues 5–26, leucines are underlined) within the *N*-terminal part of this protein and winged helix DNA-binding domain (residues 1–136 and 140–233). Upstream to the *repA* gene, the putative origin of replication (*oriV*) was found (position 33,081−33,234 in the contig00104). It is composed of five 20-bp-long direct repeats DR1.1-1.5 (5′-ATAACACTAAATGATGTGGG-3′) followed by a pair of 19-bp long inverted repeats, IR1 and IR2 (5′-AACACAACGTATAACAATA-3′ and 5′-TATTGTTATACGTTGTGTT-3′, respectively) (Fig. 2).

Moreover, within the predicted pP32BP2-contig, genes encoding a pair of partitioning proteins, ParA and ParB (GenBank: OXL18944 and OXL18945, respectively), and centromer-like sequence *parS*, were found. ParA contains the ParA domain (residues 4-182) and shows 93% identity to ParA-like protein (GenBank: ABE76262) encoded within the plasmid 1 of *Psychrobacter cryohalolentis* K5. Within the ParB protein, the ParG domain, specific to the ParB-like proteins of, e.g., *Salmonella enterica* plasmid TP228 and *Pseudomonas alcaligenes* plasmid pRA2, was found (Hayes 2000). The predicted ParB protein shows 90% identity to the hypothetical protein (GenBank: ALF60955) of the plasmid 5 of *Psychrobacter urativorans* R10.10B. The putative *parS* centomer-like sequence is located upstream to the *parA* gene. It consists of eight direct repeats DR2.1-2.8 (5′-(A/T)ATACTCA-3′) (Fig. 2). Similar *parS* site organization was reported for the pP62BP1 plasmid (Lasek et al. 2012, 2017).

### Construction of novel Psychrobacter-specific shuttle vectors

Identification of the replication module of a relatively large (approx. 60 kb) plasmid pP32BP2, encouraged us to use this system for the construction of novel *Psychrobacter*-specific cloning vectors. Currently, all available *Psychrobacter*-specific vectors are based on replication systems of small (not exceeding 6.4 kb) plasmids, which may exclude their application as molecular tools for cloning of large genetic modules. Since *Psychrobacter* spp. recently became a model bacterium representing cold-active microorganisms (Bakermans et al. 2007, 2009; Bergholz et al. 2009; Bakermans 2018), we recognized an urgent need for development of novel *Psychrobacter*-specific vectors with an increased carrying capacity.

**Fig. 3:**
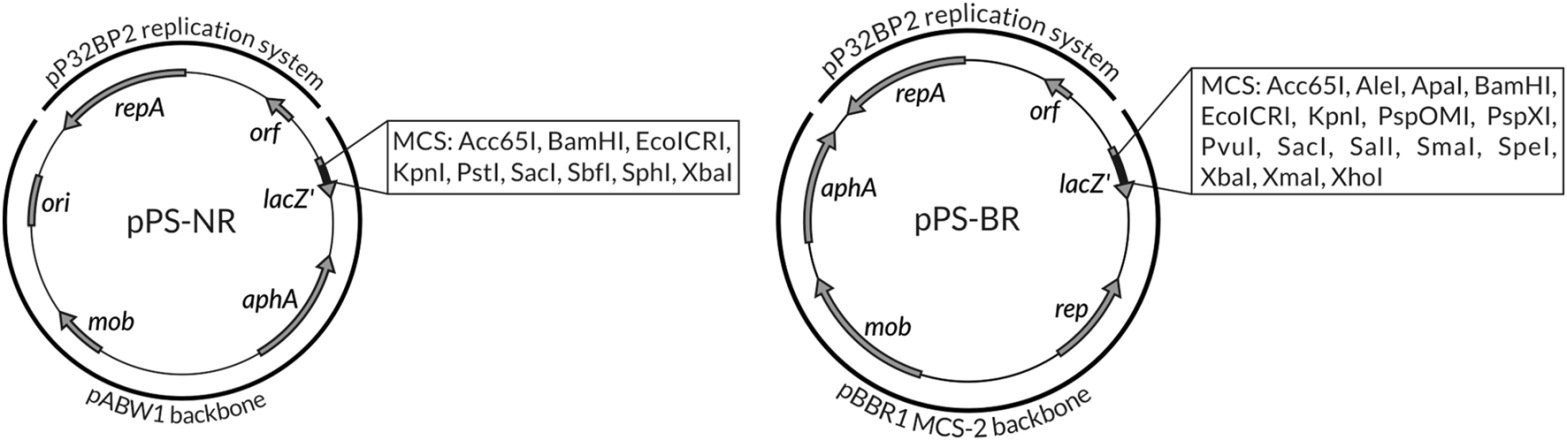
Genetic organization of the shuttle vectors pPS-NR and pPS-BR. Grey arrows indicate genes and their transcriptional orientation: *aphA* kanamycin resistance gene, *lacZ’* partial *β*-galactosidase gene, *mob* relaxase gene enabling mobilization to conjugal transfer, *orf* gene encoding conserved hypothetical protein, *rep* gene encoding replication initiation protein of the pBBR1 MCS-2 vector, *repA* gene encoding replication initiation protein of the pP32BP2 plasmid. The ColE1-type origin (*ori*) of replication of the pABW1 vector and MCSs of both plasmids are indicated

**Fig. 4:**
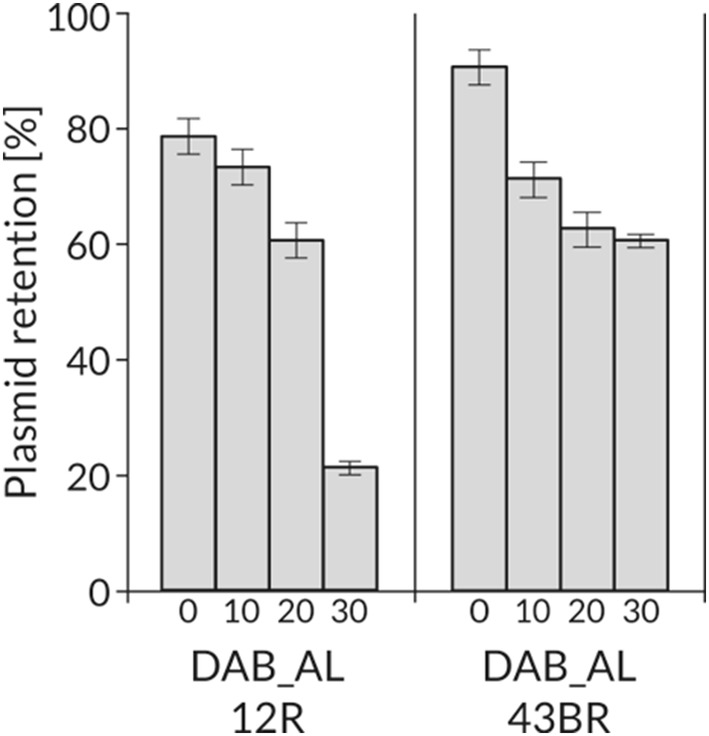
Segregational stability of shuttle vector pPS-NR tested in *Psychrobacter* sp. DAB_AL12R and DAB_AL43BR strains. The numbers below the bars indicate the number of generations grown under non-selective pressure. Error bars represent standard deviations

In this study, two novel molecular tools, suitable for genetic engineering in *Psychrobacter* spp., were constructed (Fig. 3). The PCR-amplified (1.8-kb) replication system of the pP32BP2 plasmid was cloned into the PfoI site of the pABW1 vector. This enabled construction of a narrow host range *Psychrobacter*-*E. coli* shuttle vector, pPS-NR (6,250 bp). Its functionality was confirmed by introducing it (via triparental mating) into two *Psychrobacter* spp. strains, DAB_AL12R and DAB_AL43BR. Vector stability was tested in both these strains, and after approximately 30 generations of growth without antibiotic selection pressure, plasmid pPS-NR was found in 21% and 61% of the host cells, respectively (Fig. 4).

In the course of our preliminary analyses, we surprisingly revealed that the commonly-used broad host range pBBR1 MCS-2 vector carrying the pBBR1-type replication system functional in a variety of Gram-negative bacteria, including: *Agrobacterium tumefaciens, E. coli, Bordetella pertusis, Klebsiella pneumoniae, Pseudomonas aeruginosa, Pseudomonas putida, Pseudomonas stutzeri, Rhizobium meliloti*, and *Vibrio cholerae* (Szpirer et al. 2001) is not able to replicate in tested *Psychrobacter* spp. As we are planning to conduct functional analyses of selected *Psychrobacter* sp. DAB_AL32B genes in various members of *Proteobacteria*, we constructed the second shuttle vector based on the above-mentioned broad host range plasmid pBBR1 MCS-2.

For the construction of this vector, the PCR-amplified (1.8-kb) DNA fragment containing the replication system of the pP32BP2 plasmid was cloned within PfoI site of the pBBR1 MCS-2 vector. This resulted in construction of a unique *Psychrobacter*-various *Proteobacteria* shuttle vector, pPS-BR (6929 bp). The vector was successfully introduced via triparental mating into *Psychrobacter* sp. DAB_AL12R and DAB_AL43BR. To analyse the carrying capacity of the pPS-BR vector, we cloned and successfully introduced to *Psychrobacter* spp. two relatively large restriction fragments of the DAB_AL32B genome, of a length of 5.8 kb (approx. the size of the vector) and 12.7 kb (approx. two times bigger than the vector), respectively.

Both vectors, pPS-NR and pPS-BR, carry: (i–ii) two replication systems, which enable their replication in *Psychrobacter* spp. and *E. coli* (pPS-NR) or various *Proteobacteria* (pPS-BR), (iii) the system enabling mobilization to conjugal transfer, (iv) *aphA* gene-conferring resistance to kanamycin, (v) multiple cloning site (MCS) and (vi) a selection marker (*lacZ’* gene) enabling blue-white screening of clones in *E. coli* (Fig. 3). The obtained vectors are novel and convenient tools for conducting genetic manipulations in *Psychrobacter* spp. and other *Proteobacteria* species.

## Electronic supplementary material

Below is the link to the electronic supplementary material.

Supplementary material 1 (DOCX 308 KB)

Supplementary material 2 (DOCX 50 KB)

Supplementary material 3 (DOCX 31 KB)
