# Supplementary material for: Exploring the genome of Arctic Psychrobacter sp. DAB_AL32B and construction of novel Psychrobacter-specific cloning vectors of an increased carrying capacity
Source: Arch Microbiol. 2018 Nov 17;201(5):559–69. doi: 10.1007/s00203-018-1595-y (PMC6579772; doi:10.1007/s00203-018-1595-y)
Supplement: Supplementary file 1 — Supplementary material 1 (DOCX 308 KB) [file 203_2018_1595_MOESM1_ESM.docx]

**Exploring the genome of Arctic *Psychrobacter* sp. DAB_AL32B and construction of novel *Psychrobacter*-specific cloning vectors of an increased carrying capacity**

Anna Ciok^1^, Lukasz Dziewit^1,^ *

^1^ University of Warsaw, Faculty of Biology, Institute of Microbiology, Department of Bacterial Genetics, Miecznikowa 1, 02-096 Warsaw, Poland

* Corresponding author:

Lukasz Dziewit

University of Warsaw

Faculty of Biology

Institute of Microbiology

Department of Bacterial Genetics

Miecznikowa 1, 02-096 Warsaw, Poland

tel: 48 225541406; fax: 48 225541402;

e-mail: [ldziewit@biol.uw.edu.pl](mailto:ldziewit@biol.uw.edu.pl)

**Table S1.** Open reading frames identified within the *Psychrobacter* sp. DAB_AL32B draft genome.

| Contig GenBank accession number | Gene start | Gene stop | Gene length (bp)^a^ | Predicted protein function | Protein GenBank accession number |
| --- | --- | --- | --- | --- | --- |
| NEXU01000001 | 284 | <1 | N/D | hypothetical protein | OXL28995.1 |
| NEXU01000001 | 1136 | 534 | 603 | polyisoprenoid-binding protein | OXL28996.1 |
| NEXU01000001 | 1418 | 2428 | 1011 | DNA topoisomerase I | OXL28997.1 |
| NEXU01000001 | 4169 | 2526 | 1644 | choline dehydrogenase | OXL28998.1 |
| NEXU01000001 | 4681 | 4355 | 327 | UDP-glucose 4-epimerase | OXL28999.1 |
| NEXU01000001 | 5921 | 4893 | 1029 | alcohol dehydrogenase AdhP | OXL29000.1 |
| NEXU01000001 | 7546 | 6020 | 1527 | aldehyde dehydrogenase | OXL29001.1 |
| NEXU01000001 | 7846 | 9771 | 1926 | helicase | OXL29006.1 |
| NEXU01000001 | 9895 | 11070 | 1176 | erythrose-4-phosphate dehydrogenase | OXL29002.1 |
| NEXU01000001 | 11163 | 11253 | 91 | tRNA-Ser |  |
| NEXU01000001 | 12061 | 11330 | 732 | hypothetical protein | OXL29007.1 |
| NEXU01000001 | 12706 | 12377 | 330 | CopG family transcriptional regulator | OXL29003.1 |
| NEXU01000001 | 12984 | 12909 | 76 | tRNA-Asn |  |
| NEXU01000001 | 13397 | 13885 | 489 | metal-dependent hydrolase | OXL29004.1 |
| NEXU01000001 | 13933 | >14402 | N/D | hypothetical protein | OXL29005.1 |
| NEXU01000002 | 440 | >692 | N/D | hypothetical protein | OXL28994.1 |
| NEXU01000003 | <1 | 389 | N/D | hypothetical protein | OXL28841.1 |
| NEXU01000003 | 1133 | 771 | 363 | hypothetical protein | OXL28842.1 |
| NEXU01000003 | 1414 | 1863 | 450 | hypothetical protein | OXL28843.1 |
| NEXU01000003 | 1860 | 2666 | 807 | hypothetical protein | OXL28844.1 |
| NEXU01000003 | 3811 | 3065 | 747 | HAD family hydrolase | OXL28845.1 |
| NEXU01000003 | 4953 | 3811 | 1143 | FAD-dependent oxidoreductase | OXL28846.1 |
| NEXU01000003 | 6093 | 5089 | 1005 | ABC transporter substrate-binding protein | OXL28847.1 |
| NEXU01000003 | 7860 | 6178 | 1683 | iron ABC transporter permease | OXL28848.1 |
| NEXU01000003 | 8818 | 7919 | 900 | ABC transporter | OXL28849.1 |
| NEXU01000003 | 9389 | 11149 | 1761 | ATPase | OXL28850.1 |
| NEXU01000003 | 11207 | 11908 | 702 | potassium transporter TrkA | OXL28851.1 |
| NEXU01000003 | 11927 | 12472 | 546 | hypothetical protein | OXL28852.1 |
| NEXU01000003 | 12674 | 13540 | 867 | hypothetical protein | OXL28853.1 |
| NEXU01000003 | 14806 | 13631 | 1176 | class C beta-lactamase | OXL28854.1 |
| NEXU01000003 | 15690 | 14986 | 705 | hypothetical protein | OXL28873.1 |
| NEXU01000003 | 17477 | 15975 | 1503 | tripartite tricarboxylate transporter TctA | OXL28855.1 |
| NEXU01000003 | 17938 | 17483 | 456 | hypothetical protein | OXL28856.1 |
| NEXU01000003 | 19098 | 18055 | 1044 | tricarboxylic transporter | OXL28857.1 |
| NEXU01000003 | 20690 | 19401 | 1290 | C4-dicarboxylate ABC transporter | OXL28858.1 |
| NEXU01000003 | 20992 | 21717 | 726 | GntR family transcriptional regulator | OXL28859.1 |
| NEXU01000003 | 22153 | 23148 | 996 | diaminopimelate dehydrogenase | OXL28860.1 |
| NEXU01000003 | 23648 | 23905 | 258 | twin-arginine translocase subunit TatA | OXL28861.1 |
| NEXU01000003 | 23961 | 24347 | 387 | twin-arginine translocase subunit TatB | OXL28862.1 |
| NEXU01000003 | 24344 | 25213 | 870 | twin-arginine translocase subunit TatC | OXL28863.1 |
| NEXU01000003 | 25900 | 25475 | 426 | hypothetical protein | OXL28864.1 |
| NEXU01000003 | 26427 | 28505 | 2079 | heme lyase NrfEFG subunit NrfE | OXL28865.1 |
| NEXU01000003 | 28510 | 29100 | 591 | thiol:disulfide interchange protein | OXL28866.1 |
| NEXU01000003 | 29097 | 29768 | 672 | hypothetical protein | OXL28867.1 |
| NEXU01000003 | 29765 | 31045 | 1281 | c-type cytochrome biogenesis protein CcmI | OXL28868.1 |
| NEXU01000003 | 31906 | 33132 | 1227 | nitrate reductase | OXL28874.1 |
| NEXU01000003 | 33161 | 35683 | 2523 | trimethylamine-N-oxide reductase TorA | OXL28869.1 |
| NEXU01000003 | 35942 | 36607 | 666 | molecular chaperone TorD | OXL28875.1 |
| NEXU01000003 | 37041 | 36841 | 201 | hypothetical protein |  |
| NEXU01000003 | 37195 | 37119 | 77 | tRNA-Val |  |
| NEXU01000003 | 37505 | 38482 | 978 | Gluthatione S-transferase | OXL28870.1 |
| NEXU01000003 | 39602 | 38595 | 1008 | delta-aminolevulinic acid dehydratase | OXL28871.1 |
| NEXU01000003 | 41520 | 39781 | 1740 | transporter | OXL28872.1 |
| NEXU01000004 | 394 | 23 | 372 | transporter |  |
| NEXU01000004 | 3690 | 391 | 3300 | hydrophobe/amphiphile efflux-1 family RND transporter | OXL28785.1 |
| NEXU01000004 | 5117 | 3726 | 1392 | efflux transporter periplasmic adaptor subunit | OXL28786.1 |
| NEXU01000004 | 6784 | 5735 | 1050 | geranylgeranyl diphosphate synthase | OXL28787.1 |
| NEXU01000004 | 7340 | 7651 | 312 | 50S ribosomal protein L21 | OXL28788.1 |
| NEXU01000004 | 7746 | 8003 | 258 | 50S ribosomal protein L27 | OXL28789.1 |
| NEXU01000004 | 8535 | 9236 | 702 | outer membrane lipoprotein carrier protein LolA | OXL28790.1 |
| NEXU01000004 | 10049 | 9465 | 585 | SMC-Scp complex subunit ScpB | OXL28791.1 |
| NEXU01000004 | 11021 | 10107 | 915 | segregation/condensation protein A | OXL28792.1 |
| NEXU01000004 | 12146 | 11058 | 1089 | tryptophan--tRNA ligase | OXL28793.1 |
| NEXU01000004 | 13402 | 12299 | 1104 | lipid A biosynthesis acyltransferase | OXL28794.1 |
| NEXU01000004 | 14088 | 13702 | 387 | 30S ribosomal protein S9 | OXL28795.1 |
| NEXU01000004 | 14527 | 14099 | 429 | 50S ribosomal protein L13 | OXL28796.1 |
| NEXU01000004 | 15427 | 14741 | 687 | 16S rRNA (guanine(966)-N(2))-methyltransferase RsmD | OXL28797.1 |
| NEXU01000004 | 17476 | 15464 | 2013 | penicillin-binding protein 2 | OXL28798.1 |
| NEXU01000004 | 18036 | 19370 | 1335 | hypothetical protein | OXL28799.1 |
| NEXU01000004 | 19611 | 21638 | 2028 | heme lyase NrfEFG subunit NrfE | OXL28800.1 |
| NEXU01000004 | 21640 | 22290 | 651 | thiol:disulfide interchange protein | OXL28801.1 |
| NEXU01000004 | 22287 | 22853 | 567 | cytochrome C biogenesis protein | OXL28802.1 |
| NEXU01000004 | 22850 | 24130 | 1281 | c-type cytochrome biogenesis protein CcmI | OXL28803.1 |
| NEXU01000004 | 24358 | 25014 | 657 | adenylate kinase | OXL28804.1 |
| NEXU01000004 | 25681 | 25229 | 453 | protein-export chaperone SecB | OXL28805.1 |
| NEXU01000004 | 26140 | 25877 | 264 | glutaredoxin | OXL28806.1 |
| NEXU01000004 | 26876 | 26421 | 456 | rhodanese | OXL28807.1 |
| NEXU01000004 | 28116 | 27013 | 1104 | ribosome small subunit-dependent GTPase A | OXL28808.1 |
| NEXU01000004 | 28320 | 28907 | 588 | oligoribonuclease | OXL28809.1 |
| NEXU01000004 | 29525 | 29046 | 480 | hypothetical protein | OXL28810.1 |
| NEXU01000004 | 29586 | 30023 | 438 | copper resistance protein CopD | OXL28811.1 |
| NEXU01000004 | 31207 | 30029 | 1179 | diaminopropionate ammonia-lyase | OXL28812.1 |
| NEXU01000004 | 31537 | 31238 | 300 | hypothetical protein | OXL28813.1 |
| NEXU01000005 | 299 | 1939 | 1641 | cardiolipin synthase | OXL28746.1 |
| NEXU01000005 | 2041 | 3582 | 1542 | hypothetical protein | OXL28747.1 |
| NEXU01000005 | 3705 | 5294 | 1590 | 3-deoxy-D-manno-octulosonic acid transferase | OXL28748.1 |
| NEXU01000005 | 5315 | 6091 | 777 | 16S rRNA (uracil(1498)-N(3))-methyltransferase | OXL28749.1 |
| NEXU01000005 | 6352 | 7476 | 1125 | Fe(3+) ABC transporter substrate-binding protein | OXL28750.1 |
| NEXU01000005 | 7545 | 9221 | 1677 | ABC transporter permease | OXL28751.1 |
| NEXU01000005 | 9563 | 10714 | 1152 | iron ABC transporter ATP-binding protein | OXL28752.1 |
| NEXU01000006 | 711 | <1 | N/D | SAM-dependent methyltransferase | OXL28678.1 |
| NEXU01000006 | 1228 | 4044 | 2817 | DNA gyrase subunit A | OXL28679.1 |
| NEXU01000006 | 4156 | 5226 | 1071 | protein RarD | OXL28680.1 |
| NEXU01000006 | 5517 | 6185 | 669 | glutathione S-transferase | OXL28681.1 |
| NEXU01000006 | 6288 | 6545 | 258 | hypothetical protein | OXL28682.1 |
| NEXU01000006 | 6578 | 7726 | 1149 | MBL fold metallo-hydrolase | OXL28683.1 |
| NEXU01000006 | 8755 | 7742 | 1014 | type I restriction endonuclease | OXL28684.1 |
| NEXU01000006 | 8884 | 9897 | 1014 | serine/threonine protein kinase | OXL28720.1 |
| NEXU01000006 | 10416 | 9904 | 513 | hypothetical protein | OXL28685.1 |
| NEXU01000006 | 10616 | 10532 | 85 | tRNA-Leu |  |
| NEXU01000006 | 12566 | 10839 | 1728 | nitrogen regulation protein NR(I) | OXL28686.1 |
| NEXU01000006 | 14082 | 12649 | 1434 | two-component sensor histidine kinase | OXL28687.1 |
| NEXU01000006 | 14218 | 15819 | 1602 | 30S ribosomal protein S12 methylthiotransferase RimO | OXL28688.1 |
| NEXU01000006 | 16002 | 16727 | 726 | UMP kinase | OXL28689.1 |
| NEXU01000006 | 16860 | 17414 | 555 | ribosome recycling factor | OXL28690.1 |
| NEXU01000006 | 17730 | 18488 | 759 | di-trans,poly-cis-decaprenylcistransferase | OXL28691.1 |
| NEXU01000006 | 18664 | 19473 | 810 | phosphatidate cytidylyltransferase | OXL28692.1 |
| NEXU01000006 | 19589 | 20797 | 1209 | 1-deoxy-D-xylulose-5-phosphate reductoisomerase | OXL28693.1 |
| NEXU01000006 | 20872 | 22266 | 1395 | RIP metalloprotease RseP | OXL28694.1 |
| NEXU01000006 | 22530 | 24956 | 2427 | outer membrane protein assembly factor BamA | OXL28695.1 |
| NEXU01000006 | 25107 | 26123 | 1017 | UDP-3-O-(3-hydroxymyristoyl)glucosamine N-acyltransferase | OXL28696.1 |
| NEXU01000006 | 26214 | 26723 | 510 | 3-hydroxyacyl-[acyl-carrier-protein] dehydratase FabZ | OXL28697.1 |
| NEXU01000006 | 26905 | 27684 | 780 | acyl-[acyl-carrier-protein]--UDP-N- acetylglucosamine O-acyltransferase | OXL28698.1 |
| NEXU01000006 | 27992 | 29344 | 1353 | sodium-dependent transporter | OXL28699.1 |
| NEXU01000006 | 30685 | 29534 | 1152 | tRNA guanosine(34) transglycosylase Tgt | OXL28700.1 |
| NEXU01000006 | 30894 | 31571 | 678 | heme-binding protein | OXL28701.1 |
| NEXU01000006 | 32764 | 31631 | 1134 | aspartate-semialdehyde dehydrogenase | OXL28702.1 |
| NEXU01000006 | 33197 | 34333 | 1137 | endonuclease | OXL28703.1 |
| NEXU01000006 | 36512 | 35112 | 1401 | peptidylprolyl isomerase | OXL28704.1 |
| NEXU01000006 | 39799 | 36686 | 3114 | LPS biosynthesis protein | OXL28705.1 |
| NEXU01000006 | 40174 | 41313 | 1140 | aminoglycoside phosphotransferase | OXL28706.1 |
| NEXU01000006 | 41400 | 42125 | 726 | nucleotidyl transferase | OXL28707.1 |
| NEXU01000006 | 42198 | 42554 | 357 | hypothetical protein | OXL28708.1 |
| NEXU01000006 | 44075 | 43059 | 1017 | cytochrome d ubiquinol oxidase subunit II | OXL28709.1 |
| NEXU01000006 | 45598 | 44075 | 1524 | cytochrome ubiquinol oxidase subunit I | OXL28710.1 |
| NEXU01000006 | 48584 | 46065 | 2520 | alpha/beta hydrolase | OXL28711.1 |
| NEXU01000006 | 50064 | 48979 | 1086 | hypothetical protein | OXL28712.1 |
| NEXU01000006 | 50953 | 50309 | 645 | demethoxyubiquinone hydroxylase family protein | OXL28713.1 |
| NEXU01000006 | 50966 | 51163 | 198 | hypothetical protein | OXL28714.1 |
| NEXU01000006 | 52500 | 51160 | 1341 | MFS transporter | OXL28715.1 |
| NEXU01000006 | 52973 | 56368 | 3396 | outer membrane protein assembly factor | OXL28716.1 |
| NEXU01000006 | 56492 | 61486 | 4995 | hypothetical protein | OXL28717.1 |
| NEXU01000006 | 61774 | 61610 | 165 | hemin uptake protein HemP | OXL28718.1 |
| NEXU01000006 | 61980 | >62358 | N/D | NUDIX hydrolase | OXL28719.1 |
| NEXU01000007 | 67 | 966 | 900 | NUDIX hydrolase | OXL28594.1 |
| NEXU01000007 | 1417 | 2727 | 1311 | lipocalin | OXL28572.1 |
| NEXU01000007 | 3103 | 4548 | 1446 | glyceraldehyde-3-phosphate dehydrogenase | OXL28573.1 |
| NEXU01000007 | 4810 | 6030 | 1221 | GTPase ObgE | OXL28574.1 |
| NEXU01000007 | 6102 | 7274 | 1173 | glutamate 5-kinase | OXL28575.1 |
| NEXU01000007 | 7402 | 8538 | 1137 | uroporphyrinogen decarboxylase | OXL28576.1 |
| NEXU01000007 | 8588 | 9655 | 1068 | N-acetyl-gamma-glutamyl-phosphate reductase | OXL28577.1 |
| NEXU01000007 | 9759 | 10649 | 891 | hypothetical protein | OXL28578.1 |
| NEXU01000007 | 10777 | 11184 | 408 | Clp protease ClpS | OXL28579.1 |
| NEXU01000007 | 11888 | 14437 | 2550 | ATP-dependent Clp protease ATP-binding subunit ClpA | OXL28580.1 |
| NEXU01000007 | 14616 | 15353 | 738 | uracil-DNA glycosylase | OXL28581.1 |
| NEXU01000007 | 15354 | 15947 | 594 | tRNA adenosine(34) deaminase TadA | OXL28582.1 |
| NEXU01000007 | 16108 | 16863 | 756 | cytidylate kinase | OXL28583.1 |
| NEXU01000007 | 17188 | 18879 | 1692 | 30S ribosomal protein S1 | OXL28584.1 |
| NEXU01000007 | 19177 | 19485 | 309 | integration host factor subunit beta | OXL28585.1 |
| NEXU01000007 | 19647 | 20057 | 411 | hypothetical protein | OXL28586.1 |
| NEXU01000007 | 20127 | 20825 | 699 | orotidine-5'-phosphate decarboxylase | OXL28587.1 |
| NEXU01000007 | 21063 | 21860 | 798 | metallophosphoesterase | OXL28588.1 |
| NEXU01000007 | 22038 | 22874 | 837 | histidine phosphatase family protein | OXL28589.1 |
| NEXU01000007 | 23267 | 23001 | 267 | 30S ribosomal protein S20 | OXL28590.1 |
| NEXU01000007 | 23767 | 24468 | 702 | oxidoreductase | OXL28591.1 |
| NEXU01000007 | 24580 | 25113 | 534 | ribonuclease activity regulator protein RraA | OXL28592.1 |
| NEXU01000007 | 25388 | >25665 | N/D | hypothetical protein | OXL28593.1 |
| NEXU01000008 | 22 | 3498 | 3477 | transcription-repair coupling factor |  |
| NEXU01000008 | 3704 | 4135 | 432 | HIT family protein | OXL28472.1 |
| NEXU01000008 | 4438 | 5859 | 1422 | guanylate cyclase | OXL28473.1 |
| NEXU01000008 | 6395 | 6057 | 339 | ISC system 2Fe-2S type ferredoxin | OXL28474.1 |
| NEXU01000008 | 8362 | 6485 | 1878 | Fe-S protein assembly chaperone HscA | OXL28475.1 |
| NEXU01000008 | 9336 | 8755 | 582 | Fe-S protein assembly co-chaperone HscB | OXL28476.1 |
| NEXU01000008 | 9712 | 9392 | 321 | iron-sulfur cluster assembly protein IscA | OXL28477.1 |
| NEXU01000008 | 10328 | 9936 | 393 | Fe-S cluster assembly scaffold IscU | OXL28478.1 |
| NEXU01000008 | 11743 | 10517 | 1227 | IscS subfamily cysteine desulfurase | OXL28479.1 |
| NEXU01000008 | 12252 | 11740 | 513 | Rrf2 family transcriptional regulator | OXL28480.1 |
| NEXU01000008 | 13444 | 12797 | 648 | hypothetical protein | OXL28481.1 |
| NEXU01000008 | 13965 | 14237 | 273 | DNA-binding protein HU | OXL28482.1 |
| NEXU01000008 | 14643 | 16517 | 1875 | peptidylprolyl isomerase | OXL28483.1 |
| NEXU01000008 | 18282 | 16702 | 1581 | bifunctional phosphoribosylaminoimidazolecarboxamide formyltransferase/IMP cyclohydrolase | OXL28484.1 |
| NEXU01000008 | 18828 | 18469 | 360 | Fis family transcriptional regulator | OXL28485.1 |
| NEXU01000008 | 20283 | 19006 | 1278 | thioredoxin | OXL28486.1 |
| NEXU01000008 | 21388 | 20462 | 927 | 50S ribosomal protein L11 methyltransferase | OXL28487.1 |
| NEXU01000008 | 22391 | 21603 | 789 | hypothetical protein | OXL28488.1 |
| NEXU01000008 | 24911 | 22422 | 2490 | penicillin-binding protein 1B | OXL28489.1 |
| NEXU01000008 | 25351 | 28188 | 2838 | heavy metal translocating P-type ATPase | OXL28490.1 |
| NEXU01000008 | 29021 | 28299 | 723 | hypothetical protein | OXL28491.1 |
| NEXU01000008 | 29317 | 30336 | 1020 | NAD kinase | OXL28492.1 |
| NEXU01000008 | 30392 | 30703 | 312 | hypothetical protein | OXL28493.1 |
| NEXU01000008 | 31183 | 30809 | 375 | HopJ type III effector protein | OXL28494.1 |
| NEXU01000008 | 31522 | 31277 | 246 | carbon storage regulator | OXL28495.1 |
| NEXU01000008 | 33117 | 31831 | 1287 | aspartate kinase | OXL28496.1 |
| NEXU01000008 | 36146 | 33474 | 2673 | alanine--tRNA ligase | OXL28497.1 |
| NEXU01000008 | 38081 | 36393 | 1689 | exopolyphosphatase | OXL28498.1 |
| NEXU01000008 | 38398 | 38610 | 213 | hypothetical protein | OXL28499.1 |
| NEXU01000008 | 40251 | 38824 | 1428 | murein L,D-transpeptidase | OXL28500.1 |
| NEXU01000008 | 40541 | 41716 | 1176 | 50S ribosomal protein L3 N(5)-glutamine methyltransferase | OXL28501.1 |
| NEXU01000008 | 41774 | 42877 | 1104 | chorismate synthase | OXL28502.1 |
| NEXU01000008 | 43228 | 43028 | 201 | hypothetical protein | OXL28503.1 |
| NEXU01000008 | 45188 | 43404 | 1785 | two-component system sensor histidine kinase CreC | OXL28504.1 |
| NEXU01000008 | 46092 | 45277 | 816 | DNA-binding response regulator | OXL28505.1 |
| NEXU01000008 | 47821 | 46454 | 1368 | glycerophosphodiester phosphodiesterase | OXL28506.1 |
| NEXU01000008 | 47982 | 48653 | 672 | carboxylesterase | OXL28507.1 |
| NEXU01000008 | 49569 | 50581 | 1013 | IS3 family transposase |  |
| NEXU01000009 | 573 | <1 | N/D | hypothetical protein | OXL28310.1 |
| NEXU01000009 | 1478 | 801 | 678 | hypothetical protein | OXL28311.1 |
| NEXU01000009 | 4066 | 1526 | 2541 | hypothetical protein | OXL28312.1 |
| NEXU01000009 | 4814 | 4068 | 747 | hypothetical protein | OXL28313.1 |
| NEXU01000009 | >5655 | 4848 | N/D | hypothetical protein | OXL28314.1 |
| NEXU01000010 | 1902 | <1 | N/D | hypothetical protein | OXL27997.1 |
| NEXU01000010 | 2080 | 2598 | 519 | hypothetical protein | OXL27998.1 |
| NEXU01000010 | 2618 | 3424 | 807 | hypothetical protein | OXL27999.1 |
| NEXU01000010 | 3452 | 3964 | 513 | type VI secretion system-associated protein | OXL28000.1 |
| NEXU01000010 | 3968 | 5458 | 1491 | EvpB family type VI secretion protein | OXL28001.1 |
| NEXU01000010 | 5513 | 6016 | 504 | Hcp1 family type VI secretion system effector | OXL28002.1 |
| NEXU01000010 | 6092 | 6601 | 510 | hypothetical protein | OXL28003.1 |
| NEXU01000010 | 6656 | 8479 | 1824 | hypothetical protein | OXL28004.1 |
| NEXU01000010 | 8443 | 9483 | 1041 | hypothetical protein | OXL28005.1 |
| NEXU01000010 | 9783 | 12683 | 2901 | ClpV1 family T6SS ATPase | OXL28006.1 |
| NEXU01000010 | 12683 | 13834 | 1152 | hypothetical protein | OXL28007.1 |
| NEXU01000010 | 13944 | 16880 | 2937 | hypothetical protein | OXL28008.1 |
| NEXU01000010 | 16883 | 18349 | 1467 | hypothetical protein | OXL28009.1 |
| NEXU01000010 | 18349 | 18780 | 432 | hypothetical protein | OXL28010.1 |
| NEXU01000010 | 19770 | 18927 | 844 | IS3 family transposase |  |
| NEXU01000010 | 19854 | 20604 | 751 | IS5/IS1182 family transposase |  |
| NEXU01000010 | 21010 | 20798 | 213 | IS3 family transposase |  |
| NEXU01000010 | 21336 | 21250 | 87 | tRNA-Leu |  |
| NEXU01000010 | 21548 | 21475 | 74 | tRNA-Cys |  |
| NEXU01000010 | 22821 | 21763 | 1059 | sulfurtransferase FdhD | OXL28011.1 |
| NEXU01000010 | 25224 | 22882 | 2343 | formate dehydrogenase | OXL28012.1 |
| NEXU01000010 | 26396 | 25449 | 948 | cytochrome C | OXL28013.1 |
| NEXU01000010 | 27781 | 26396 | 1386 | alcohol dehydrogenase | OXL28014.1 |
| NEXU01000010 | 27955 | 30096 | 2142 | hypothetical protein | OXL28015.1 |
| NEXU01000010 | 30100 | 30714 | 615 | hypothetical protein | OXL28016.1 |
| NEXU01000010 | 31666 | 30872 | 795 | ABC transporter ATP-binding protein | OXL28017.1 |
| NEXU01000010 | 32650 | 31751 | 900 | ABC transporter permease | OXL28018.1 |
| NEXU01000010 | 34133 | 33108 | 1026 | ABC transporter substrate-binding protein | OXL28019.1 |
| NEXU01000010 | 35313 | 34270 | 1044 | ABC transporter substrate-binding protein | OXL28020.1 |
| NEXU01000010 | 35877 | 35593 | 285 | hypothetical protein | OXL28021.1 |
| NEXU01000010 | 38023 | 35921 | 2103 | "metG" methionine--tRNA ligase | OXL28022.1 |
| NEXU01000010 | 39744 | 38329 | 1416 | citrate transporter | OXL28023.1 |
| NEXU01000010 | 40119 | 40832 | 714 | succinyl-CoA--3-ketoacid-CoA transferase | OXL28024.1 |
| NEXU01000010 | 40914 | 41543 | 630 | succinyl-CoA--3-ketoacid-CoA transferase | OXL28025.1 |
| NEXU01000010 | 41670 | 42470 | 801 | 3-hydroxybutyrate dehydrogenase | OXL28026.1 |
| NEXU01000010 | 42641 | 43996 | 1356 | magnesium transporter | OXL28027.1 |
| NEXU01000010 | 44220 | >44474 | N/D | ATP-binding protein | OXL28028.1 |
| NEXU01000011 | <1 | 666 | N/D | ATP-binding protein | OXL27982.1 |
| NEXU01000011 | 718 | 1857 | 1140 | hypothetical protein | OXL27983.1 |
| NEXU01000011 | 2015 | 2590 | 576 | dCTP deaminase | OXL27984.1 |
| NEXU01000011 | 3140 | 2706 | 435 | ClpXP protease specificity-enhancing factor | OXL27985.1 |
| NEXU01000011 | 3887 | 3273 | 615 | starvation protein A | OXL27986.1 |
| NEXU01000011 | 4796 | 4083 | 714 | cytochrome C | OXL27987.1 |
| NEXU01000011 | 6022 | 4796 | 1227 | cytochrome b | OXL27988.1 |
| NEXU01000011 | 6608 | 6024 | 585 | ubiquinol-cytochrome c reductase iron-sulfur subunit | OXL27989.1 |
| NEXU01000011 | 9222 | 7279 | 1944 | ABC transporter ATP-binding protein | OXL27990.1 |
| NEXU01000011 | 9422 | 9703 | 282 | SlyX protein | OXL27991.1 |
| NEXU01000011 | 9926 | 11161 | 1236 | hypothetical protein | OXL27992.1 |
| NEXU01000011 | 11570 | 12985 | 1416 | hypothetical protein | OXL27993.1 |
| NEXU01000011 | 13443 | 14825 | 1383 | hypothetical protein | OXL27994.1 |
| NEXU01000011 | 15113 | 18775 | 3663 | hypothetical protein | OXL27995.1 |
| NEXU01000011 | 19462 | >20296 | N/D | hypothetical protein | OXL27996.1 |
| NEXU01000012 | <1 | >1295 | N/D | hypothetical protein | OXL27836.1 |
| NEXU01000013 | <1 | 1851 | N/D | hypothetical protein | OXL27800.1 |
| NEXU01000013 | 1978 | >3517 | N/D | hypothetical protein | OXL27801.1 |
| NEXU01000014 | <1 | 215 | N/D | hypothetical protein | OXL27670.1 |
| NEXU01000014 | 2282 | 285 | 1998 | transketolase | OXL27671.1 |
| NEXU01000014 | 3847 | 2561 | 1287 | serine--tRNA ligase | OXL27672.1 |
| NEXU01000014 | 4284 | 4943 | 660 | hypothetical protein | OXL27673.1 |
| NEXU01000014 | 5296 | 5781 | 486 | Dps-like DNA binding protein | OXL27674.1 |
| NEXU01000014 | 6587 | 5880 | 708 | hypothetical protein | OXL27675.1 |
| NEXU01000014 | 7455 | 6643 | 813 | DnaA regulatory inactivator Hda | OXL27676.1 |
| NEXU01000014 | 8855 | 7749 | 1107 | AI-2E family transporter | OXL27677.1 |
| NEXU01000014 | 9228 | 10277 | 1050 | phosphoribosylformylglycinamidine cyclo-ligase | OXL27678.1 |
| NEXU01000014 | 10277 | 10978 | 702 | phosphoribosylglycinamide formyltransferase | OXL27679.1 |
| NEXU01000014 | 12523 | 11135 | 1389 | succinate-semialdehyde dehydrogenase | OXL27680.1 |
| NEXU01000014 | 13985 | 12996 | 990 | LysR family transcriptional regulator | OXL27681.1 |
| NEXU01000014 | 14689 | 16278 | 1590 | isocitrate lyase | OXL27682.1 |
| NEXU01000014 | 17664 | 16381 | 1284 | hypothetical protein | OXL27683.1 |
| NEXU01000014 | 17847 | 19463 | 1617 | inorganic triphosphatase | OXL27684.1 |
| NEXU01000014 | 20213 | 19593 | 621 | nitroreductase family protein | OXL27685.1 |
| NEXU01000014 | 21028 | 20459 | 570 | L,D-transpeptidase | OXL27686.1 |
| NEXU01000014 | 21164 | 22450 | 1287 | hypothetical protein | OXL27687.1 |
| NEXU01000014 | 23796 | 22558 | 1239 | argininosuccinate synthase | OXL27688.1 |
| NEXU01000014 | 24163 | 25233 | 1071 | dihydroorotase | OXL27689.1 |
| NEXU01000014 | 25417 | 26202 | 786 | ribonuclease T | OXL27690.1 |
| NEXU01000014 | 26773 | 26848 | 76 | tRNA-Glu |  |
| NEXU01000014 | 27917 | 27285 | 633 | hypothetical protein | OXL27691.1 |
| NEXU01000014 | 29207 | 28425 | 783 | 2-C-methyl-D-erythritol 4-phosphate cytidylyltransferase | OXL27692.1 |
| NEXU01000014 | 29672 | 29364 | 309 | cell division protein FtsB | OXL27693.1 |
| NEXU01000014 | 31100 | 29784 | 1317 | phosphopyruvate hydratase | OXL27694.1 |
| NEXU01000014 | 33813 | 31453 | 2361 | copper-translocating P-type ATPase | OXL27695.1 |
| NEXU01000014 | 34372 | 34148 | 225 | hypothetical protein | OXL27696.1 |
| NEXU01000014 | 34767 | 34513 | 255 | hypothetical protein | OXL27697.1 |
| NEXU01000014 | 35670 | 34795 | 876 | 3-deoxy-8-phosphooctulonate synthase | OXL27698.1 |
| NEXU01000014 | 37527 | 35893 | 1635 | CTP synthetase | OXL27699.1 |
| NEXU01000014 | 38714 | 37722 | 993 | hypothetical protein | OXL27700.1 |
| NEXU01000015 | 2582 | 384 | 2199 | malate synthase G | OXL27523.1 |
| NEXU01000015 | 3517 | 4152 | 636 | tRNA-(ms[2]io[6]A)-hydroxylase | OXL27544.1 |
| NEXU01000015 | 4479 | 4273 | 207 | hypothetical protein | OXL27524.1 |
| NEXU01000015 | 5106 | 4573 | 534 | hypothetical protein | OXL27545.1 |
| NEXU01000015 | 5488 | 5237 | 252 | hypothetical protein | OXL27525.1 |
| NEXU01000015 | 8286 | 6067 | 2220 | polyphosphate kinase 1 | OXL27546.1 |
| NEXU01000015 | 9171 | 8485 | 687 | monofunctional biosynthetic peptidoglycan transglycosylase | OXL27526.1 |
| NEXU01000015 | 9441 | 9755 | 315 | thioredoxin family protein | OXL27527.1 |
| NEXU01000015 | 9830 | 11038 | 1209 | alpha-hydroxy-acid oxidizing enzyme | OXL27528.1 |
| NEXU01000015 | 11137 | 12162 | 1026 | rhomboid family intramembrane serine protease | OXL27529.1 |
| NEXU01000015 | 12264 | 13076 | 813 | hypothetical protein | OXL27530.1 |
| NEXU01000015 | 13593 | 13201 | 393 | hypothetical protein | OXL27531.1 |
| NEXU01000015 | 13614 | 13964 | 351 | transposase | OXL27532.1 |
| NEXU01000015 | 14005 | 14463 | 459 | transposase | OXL27547.1 |
| NEXU01000015 | 14783 | 14550 | 234 | hypothetical protein | OXL27533.1 |
| NEXU01000015 | 16410 | 15007 | 1404 | fumarate hydratase, class II | OXL27534.1 |
| NEXU01000015 | 16608 | 17246 | 639 | 5-formyltetrahydrofolate cyclo-ligase | OXL27535.1 |
| NEXU01000015 | 17243 | 17476 | 234 | hypothetical protein | OXL27536.1 |
| NEXU01000015 | 18942 | 17581 | 1362 | sodium:proton antiporter | OXL27537.1 |
| NEXU01000015 | 19262 | 21886 | 2625 | endopeptidase La | OXL27538.1 |
| NEXU01000015 | 22014 | 23159 | 1146 | hypothetical protein | OXL27539.1 |
| NEXU01000015 | 23215 | 24366 | 1152 | hypothetical protein | OXL27540.1 |
| NEXU01000015 | 24873 | 24451 | 423 | hypothetical protein | OXL27541.1 |
| NEXU01000015 | 25170 | 25727 | 558 | superoxide dismutase | OXL27542.1 |
| NEXU01000015 | >27165 | 25830 | N/D | DNA topoisomerase I subunit omega | OXL27543.1 |
| NEXU01000016 | 1254 | <1 | N/D | DNA topoisomerase I | OXL27500.1 |
| NEXU01000016 | 2130 | 1534 | 597 | hypothetical protein | OXL27501.1 |
| NEXU01000016 | 2608 | 3081 | 474 | hypothetical protein | OXL27504.1 |
| NEXU01000016 | 3723 | 3322 | 402 | biopolymer transporter ExbD | OXL27502.1 |
| NEXU01000016 | >4269 | 3826 | N/D | biopolymer transporter ExbB | OXL27503.1 |
| NEXU01000017 | 478 | <1 | N/D | biopolymer transporter ExbB | OXL27034.1 |
| NEXU01000017 | 1516 | 563 | 954 | hypothetical protein | OXL27035.1 |
| NEXU01000017 | 1916 | 2968 | 1053 | ABC transporter substrate-binding protein | OXL27036.1 |
| NEXU01000017 | 3186 | 4124 | 939 | iron ABC transporter permease | OXL27192.1 |
| NEXU01000017 | 4229 | 5287 | 1059 | enterobactin ABC transporter permease | OXL27037.1 |
| NEXU01000017 | 5362 | 6120 | 759 | iron ABC transporter ATP-binding protein | OXL27038.1 |
| NEXU01000017 | 7503 | 6145 | 1359 | peptidase | OXL27039.1 |
| NEXU01000017 | 9758 | 7656 | 2103 | TonB-dependent receptor | OXL27040.1 |
| NEXU01000017 | 10439 | 11851 | 1413 | xanthine permease XanP | OXL27041.1 |
| NEXU01000017 | 12673 | 12008 | 666 | TetR family transcriptional regulator | OXL27042.1 |
| NEXU01000017 | 12987 | 13508 | 522 | OHCU decarboxylase | OXL27043.1 |
| NEXU01000017 | 13549 | 13917 | 369 | hydroxyisourate hydrolase | OXL27044.1 |
| NEXU01000017 | 14124 | 15458 | 1335 | hypothetical protein | OXL27045.1 |
| NEXU01000017 | 15677 | 16657 | 981 | allantoinase | OXL27046.1 |
| NEXU01000017 | 17054 | 17614 | 561 | ureidoglycolate hydrolase | OXL27047.1 |
| NEXU01000017 | 17811 | 18647 | 837 | (S)-ureidoglycine aminohydrolase | OXL27048.1 |
| NEXU01000017 | 18916 | 19194 | 279 | transcriptional regulator | OXL27049.1 |
| NEXU01000017 | 19249 | 20160 | 912 | quinone oxidoreductase | OXL27050.1 |
| NEXU01000017 | 20257 | 21675 | 1419 | anthranilate synthase component I | OXL27051.1 |
| NEXU01000017 | 21710 | 22357 | 648 | anthranilate/aminodeoxychorismate synthase component II | OXL27052.1 |
| NEXU01000017 | 23177 | 22434 | 744 | polar amino acid ABC transporter ATP-binding protein | OXL27053.1 |
| NEXU01000017 | 23987 | 23250 | 738 | cysteine ABC transporter permease | OXL27054.1 |
| NEXU01000017 | 24823 | 23987 | 837 | amino acid ABC transporter substrate-binding protein | OXL27055.1 |
| NEXU01000017 | 25907 | 25161 | 747 | GntR family transcriptional regulator | OXL27056.1 |
| NEXU01000017 | 26259 | 27977 | 1719 | urocanate hydratase | OXL27057.1 |
| NEXU01000017 | 28034 | 29596 | 1563 | histidine ammonia-lyase | OXL27058.1 |
| NEXU01000017 | 29634 | 30686 | 1053 | formimidoylglutamase | OXL27059.1 |
| NEXU01000017 | 30744 | 32087 | 1344 | imidazolonepropionase | OXL27060.1 |
| NEXU01000017 | 32430 | 32215 | 216 | hypothetical protein | OXL27061.1 |
| NEXU01000017 | 32948 | 32364 | 585 | hypothetical protein | OXL27062.1 |
| NEXU01000017 | 34582 | 33236 | 1347 | glutamate dehydrogenase | OXL27063.1 |
| NEXU01000017 | 35299 | 35661 | 363 | hypothetical protein | OXL27064.1 |
| NEXU01000017 | 35968 | 36462 | 495 | diguanylate cyclase | OXL27065.1 |
| NEXU01000017 | 36466 | 37215 | 750 | phosphate ABC transporter substrate-binding protein | OXL27066.1 |
| NEXU01000017 | 37290 | 37907 | 618 | hypothetical protein | OXL27067.1 |
| NEXU01000017 | 37961 | 38575 | 615 | hypothetical protein | OXL27068.1 |
| NEXU01000017 | 39553 | 39032 | 522 | hypothetical protein | OXL27069.1 |
| NEXU01000017 | 40398 | 39826 | 573 | GNAT family N-acetyltransferase | OXL27070.1 |
| NEXU01000017 | 41195 | 40437 | 759 | MoeA | OXL27071.1 |
| NEXU01000017 | 42466 | 41192 | 1275 | hypothetical protein | OXL27072.1 |
| NEXU01000017 | 44942 | 42522 | 2421 | xanthine dehydrogenase | OXL27073.1 |
| NEXU01000017 | 45937 | 44945 | 993 | molybdopterin dehydrogenase | OXL27074.1 |
| NEXU01000017 | 46584 | 45934 | 651 | (2Fe-2S)-binding protein | OXL27075.1 |
| NEXU01000017 | 46967 | 47620 | 654 | disulfide bond formation protein DsbA | OXL27076.1 |
| NEXU01000017 | 47648 | 47896 | 249 | hypothetical protein | OXL27077.1 |
| NEXU01000017 | 49191 | 48061 | 1131 | S-(hydroxymethyl)glutathione dehydrogenase/class III alcohol dehydrogenase | OXL27078.1 |
| NEXU01000017 | 49389 | 50303 | 915 | LysR family transcriptional regulator | OXL27079.1 |
| NEXU01000017 | 50635 | 50345 | 291 | hypothetical protein | OXL27080.1 |
| NEXU01000017 | 51208 | 50789 | 420 | hypothetical protein | OXL27081.1 |
| NEXU01000017 | 53533 | 51329 | 2205 | transglutaminase | OXL27082.1 |
| NEXU01000017 | 54579 | 53530 | 1050 | hypothetical protein | OXL27083.1 |
| NEXU01000017 | 55572 | 54613 | 960 | AAA family ATPase | OXL27084.1 |
| NEXU01000017 | 56075 | 55617 | 459 | cytochrome C | OXL27085.1 |
| NEXU01000017 | 58392 | 56299 | 2094 | diguanylate phosphodiesterase | OXL27086.1 |
| NEXU01000017 | 60345 | 58630 | 1716 | hypothetical protein | OXL27087.1 |
| NEXU01000017 | 61537 | 60530 | 1008 | EF-P beta-lysylation protein EpmB | OXL27088.1 |
| NEXU01000017 | 61758 | 62330 | 573 | elongation factor P | OXL27089.1 |
| NEXU01000017 | 64264 | 62429 | 1836 | hybrid sensor histidine kinase/response regulator | OXL27090.1 |
| NEXU01000017 | 65164 | 64274 | 891 | tRNA threonylcarbamoyladenosine dehydratase | OXL27091.1 |
| NEXU01000017 | 66266 | 65238 | 1029 | hydrolase | OXL27092.1 |
| NEXU01000017 | 69114 | 66859 | 2256 | type II secretion system protein GspD | OXL27093.1 |
| NEXU01000017 | 70156 | 69254 | 903 | general secretion pathway protein | OXL27094.1 |
| NEXU01000017 | 70981 | 70157 | 825 | general secretion pathway protein | OXL27095.1 |
| NEXU01000017 | 71561 | 71238 | 324 | DNA-binding protein | OXL27096.1 |
| NEXU01000017 | 72717 | 72049 | 669 | recombinase RecB | OXL27097.1 |
| NEXU01000017 | 74190 | 72826 | 1365 | MFS transporter | OXL27098.1 |
| NEXU01000017 | 74789 | 77695 | 2907 | excinuclease ABC subunit A | OXL27099.1 |
| NEXU01000017 | 78023 | 78949 | 927 | hypothetical protein | OXL27100.1 |
| NEXU01000017 | 79007 | 79498 | 492 | hypothetical protein | OXL27101.1 |
| NEXU01000017 | 80586 | 79744 | 843 | deoxyribonuclease HsdR | OXL27102.1 |
| NEXU01000017 | 81204 | 80923 | 282 | hypothetical protein | OXL27103.1 |
| NEXU01000017 | 81667 | 81299 | 369 | hypothetical protein | OXL27104.1 |
| NEXU01000017 | 81914 | 82129 | 216 | hypothetical protein | OXL27105.1 |
| NEXU01000017 | 82750 | 82139 | 612 | GNAT family N-acetyltransferase | OXL27106.1 |
| NEXU01000017 | 84495 | 82843 | 1653 | pyridine nucleotide-disulfide oxidoreductase | OXL27107.1 |
| NEXU01000017 | 84738 | 86048 | 1311 | tRNA pseudouridine(13) synthase TruD | OXL27108.1 |
| NEXU01000017 | 87644 | 86097 | 1548 | GGDEF domain-containing protein | OXL27109.1 |
| NEXU01000017 | 88778 | 87897 | 882 | phosphatase | OXL27110.1 |
| NEXU01000017 | 88968 | 89519 | 552 | septation protein IspZ | OXL27111.1 |
| NEXU01000017 | 89695 | 90036 | 342 | hypothetical protein | OXL27112.1 |
| NEXU01000017 | 90033 | 90497 | 465 | hypothetical protein | OXL27113.1 |
| NEXU01000017 | 90524 | 92614 | 2091 | FAD-dependent cmnm(5)s(2)U34 oxidoreductase | OXL27114.1 |
| NEXU01000017 | 93191 | 92631 | 561 | hypothetical protein | OXL27115.1 |
| NEXU01000017 | 93341 | 93973 | 633 | hypothetical protein | OXL27116.1 |
| NEXU01000017 | 94139 | 94762 | 624 | hypothetical protein | OXL27117.1 |
| NEXU01000017 | 95552 | 94881 | 672 | hypothetical protein | OXL27118.1 |
| NEXU01000017 | 96462 | 95545 | 918 | 4-hydroxybenzoate polyprenyltransferase | OXL27119.1 |
| NEXU01000017 | 96806 | 96567 | 240 | hypothetical protein | OXL27120.1 |
| NEXU01000017 | 97737 | 96847 | 891 | 16S rRNA methyltransferase | OXL27121.1 |
| NEXU01000017 | 98647 | 97805 | 843 | undecaprenyl-diphosphatase | OXL27122.1 |
| NEXU01000017 | 99448 | 98765 | 684 | tRNA (adenosine(37)-N6)-threonylcarbamoyltransferase complex dimerization subunit type 1 TsaB | OXL27193.1 |
| NEXU01000017 | 101242 | 99692 | 1551 | signal recognition particle protein | OXL27123.1 |
| NEXU01000017 | 101587 | 102393 | 807 | cytochrome C assembly protein | OXL27124.1 |
| NEXU01000017 | 103454 | 102525 | 930 | formyltetrahydrofolate deformylase | OXL27125.1 |
| NEXU01000017 | 111478 | 103652 | 7827 | hypothetical protein | OXL27126.1 |
| NEXU01000017 | 111962 | 112198 | 237 | 50S ribosomal protein L28 | OXL27127.1 |
| NEXU01000017 | 112338 | 112493 | 156 | 50S ribosomal protein L33 | OXL27128.1 |
| NEXU01000017 | 112928 | 112617 | 312 | hypothetical protein | OXL27129.1 |
| NEXU01000017 | 113442 | 113131 | 312 | hypothetical protein | OXL27130.1 |
| NEXU01000017 | 113908 | 113564 | 345 | hypothetical protein | OXL27131.1 |
| NEXU01000017 | 114686 | 113994 | 693 | ribulose-phosphate 3-epimerase | OXL27132.1 |
| NEXU01000017 | 115324 | 114845 | 480 | TIGR03643 family protein | OXL27133.1 |
| NEXU01000017 | 116290 | 115382 | 909 | S-formylglutathione hydrolase | OXL27134.1 |
| NEXU01000017 | 117874 | 116393 | 1482 | peptidase C13 family protein | OXL27135.1 |
| NEXU01000017 | 120182 | 118197 | 1986 | molecular chaperone HtpG | OXL27136.1 |
| NEXU01000017 | 122027 | 120495 | 1533 | catalase | OXL27137.1 |
| NEXU01000017 | 123200 | 122385 | 816 | thioesterase | OXL27138.1 |
| NEXU01000017 | 124219 | 123263 | 957 | 4-hydroxy-3-methylbut-2-enyl diphosphate reductase | OXL27139.1 |
| NEXU01000017 | 124575 | 125201 | 627 | guanylate kinase | OXL27140.1 |
| NEXU01000017 | 125375 | 125635 | 261 | DNA-directed RNA polymerase subunit omega | OXL27141.1 |
| NEXU01000017 | 126021 | 128210 | 2190 | guanosine-3',5'-bis(diphosphate) 3'-pyrophosphohydrolase | OXL27142.1 |
| NEXU01000017 | 128377 | 128757 | 381 | reactive intermediate/imine deaminase | OXL27143.1 |
| NEXU01000017 | 128821 | 131202 | 2382 | primosomal protein N' | OXL27144.1 |
| NEXU01000017 | 131461 | 132630 | 1170 | hypothetical protein | OXL27145.1 |
| NEXU01000017 | 133119 | 132811 | 309 | hypothetical protein | OXL27146.1 |
| NEXU01000017 | 133364 | 134068 | 705 | hypothetical protein | OXL27147.1 |
| NEXU01000017 | 134146 | 136032 | 1887 | glutathione-regulated potassium-efflux system protein KefB | OXL27148.1 |
| NEXU01000017 | 136975 | 136046 | 930 | hypothetical protein | OXL27149.1 |
| NEXU01000017 | 137172 | 137606 | 435 | hypothetical protein | OXL27150.1 |
| NEXU01000017 | 137715 | 138524 | 810 | tRNA (guanosine(46)-N7)-methyltransferase TrmB | OXL27151.1 |
| NEXU01000017 | 139564 | 140622 | 1059 | recombinase RecA | OXL27152.1 |
| NEXU01000017 | 140663 | 141778 | 1116 | inhibitor/regulator of recA, recX | OXL27153.1 |
| NEXU01000017 | 143290 | 141833 | 1458 | C4-dicarboxylate ABC transporter | OXL27154.1 |
| NEXU01000017 | 144315 | 143572 | 744 | hypothetical protein | OXL27155.1 |
| NEXU01000017 | 145013 | 144555 | 459 | hypothetical protein | OXL27156.1 |
| NEXU01000017 | 145421 | 145140 | 282 | hypothetical protein | OXL27157.1 |
| NEXU01000017 | 146473 | 145577 | 897 | dihydropteroate synthase | OXL27158.1 |
| NEXU01000017 | 148515 | 146620 | 1896 | cell division protein FtsH | OXL27159.1 |
| NEXU01000017 | 149569 | 148937 | 633 | 23S rRNA methyltransferase | OXL27160.1 |
| NEXU01000017 | 149924 | 150232 | 309 | ribosome assembly protein YhbY | OXL27161.1 |
| NEXU01000017 | 150435 | 151247 | 813 | hypothetical protein | OXL27162.1 |
| NEXU01000017 | 152102 | 151374 | 729 | CHAP domain-containing protein | OXL27163.1 |
| NEXU01000017 | 152301 | 152816 | 516 | hypothetical protein | OXL27164.1 |
| NEXU01000017 | 154077 | 153121 | 957 | UDP-3-O-[3-hydroxymyristoyl] N-acetylglucosamine deacetylase | OXL27165.1 |
| NEXU01000017 | 155582 | 154386 | 1197 | cell division protein FtsZ | OXL27166.1 |
| NEXU01000017 | 157168 | 155828 | 1341 | cell division protein FtsA | OXL27167.1 |
| NEXU01000017 | 158084 | 157287 | 798 | cell division protein FtsQ | OXL27168.1 |
| NEXU01000017 | 159524 | 158586 | 939 | D-alanine--D-alanine ligase | OXL27194.1 |
| NEXU01000017 | 161227 | 159788 | 1440 | UDP-N-acetylmuramate--L-alanine ligase | OXL27169.1 |
| NEXU01000017 | 162385 | 161300 | 1086 | undecaprenyldiphospho-muramoylpentapeptide beta-N-acetylglucosaminyltransferase | OXL27170.1 |
| NEXU01000017 | 163695 | 162724 | 972 | glutathione synthase | OXL27171.1 |
| NEXU01000017 | 164652 | 163933 | 720 | orotate phosphoribosyltransferase | OXL27172.1 |
| NEXU01000017 | 165765 | 164722 | 1044 | hypothetical protein | OXL27173.1 |
| NEXU01000017 | 166765 | 165926 | 840 | NAD(P)-dependent oxidoreductase | OXL27174.1 |
| NEXU01000017 | 169014 | 166858 | 2157 | hypothetical protein |  |
| NEXU01000017 | 169198 | 169788 | 591 | N-acetylmuramoyl-L-alanine amidase | OXL27175.1 |
| NEXU01000017 | 169935 | 171485 | 1551 | murein biosynthesis integral membrane protein MurJ | OXL27176.1 |
| NEXU01000017 | 171597 | 172232 | 636 | hypothetical protein | OXL27195.1 |
| NEXU01000017 | 173055 | 172399 | 657 | N-acetyltransferase | OXL27177.1 |
| NEXU01000017 | 173323 | 174366 | 1044 | riboflavin biosynthesis protein RibF | OXL27178.1 |
| NEXU01000017 | 174695 | 174456 | 240 | hypothetical protein | OXL27179.1 |
| NEXU01000017 | 176011 | 175028 | 984 | malate dehydrogenase | OXL27180.1 |
| NEXU01000017 | 177036 | 176377 | 660 | hypothetical protein | OXL27196.1 |
| NEXU01000017 | 179645 | 177432 | 2214 | lytic transglycosylase | OXL27181.1 |
| NEXU01000017 | 180052 | 181542 | 1491 | tRNA (N6-isopentenyl adenosine(37)-C2)-methylthiotransferase MiaB | OXL27182.1 |
| NEXU01000017 | 183618 | 181786 | 1833 | choline-sulfatase | OXL27197.1 |
| NEXU01000017 | 184636 | 183881 | 756 | threonine transporter RhtB | OXL27183.1 |
| NEXU01000017 | 185981 | 184743 | 1239 | acetylornithine deacetylase | OXL27184.1 |
| NEXU01000017 | 186514 | 186017 | 498 | tRNA-specific adenosine deaminase | OXL27185.1 |
| NEXU01000017 | 187297 | 186644 | 654 | hypothetical protein | OXL27186.1 |
| NEXU01000017 | 187517 | 187957 | 441 | hypothetical protein | OXL27187.1 |
| NEXU01000017 | 188073 | 188534 | 462 | cell division protein ZapA | OXL27188.1 |
| NEXU01000017 | 188946 | 188635 | 312 | hypothetical protein | OXL27189.1 |
| NEXU01000017 | 191172 | 189079 | 2094 | glycine--tRNA ligase subunit beta | OXL27190.1 |
| NEXU01000017 | 192183 | 191182 | 1002 | glycine--tRNA ligase subunit alpha | OXL27198.1 |
| NEXU01000017 | 192834 | 192622 | 213 | stress-responsive transcriptional regulator | OXL27191.1 |
| NEXU01000018 | 505 | 2319 | 1815 | trifunctional thioredoxin/methionine sulfoxide reductase A/B protein | OXL26819.1 |
| NEXU01000018 | 2610 | 3851 | 1242 | nicotinate phosphoribosyltransferase | OXL26820.1 |
| NEXU01000018 | 4108 | 3863 | 246 | exodeoxyribonuclease VII | OXL26821.1 |
| NEXU01000018 | 5736 | 4177 | 1560 | exodeoxyribonuclease VII large subunit | OXL26822.1 |
| NEXU01000018 | 6317 | 5949 | 369 | hypothetical protein | OXL26823.1 |
| NEXU01000018 | 6733 | 7392 | 660 | alpha/beta hydrolase | OXL26824.1 |
| NEXU01000018 | 7643 | 8749 | 1107 | cell division protein ZapE | OXL26825.1 |
| NEXU01000018 | 8918 | 9574 | 657 | nitroreductase | OXL26826.1 |
| NEXU01000018 | 9777 | 11087 | 1311 | glycerol-3-phosphate dehydrogenase | OXL26827.1 |
| NEXU01000018 | 11099 | 11557 | 459 | phosphohistidine phosphatase SixA | OXL26828.1 |
| NEXU01000018 | 11780 | 13087 | 1308 | hypothetical protein | OXL26829.1 |
| NEXU01000018 | 13182 | 13739 | 558 | hypothetical protein | OXL26830.1 |
| NEXU01000018 | 14018 | 14470 | 453 | hypothetical protein | OXL26870.1 |
| NEXU01000018 | 14670 | 15704 | 1035 | dihydroorotate dehydrogenase (quinone) | OXL26831.1 |
| NEXU01000018 | 15868 | 16365 | 498 | colicin V production protein | OXL26832.1 |
| NEXU01000018 | 16607 | 18142 | 1536 | amidophosphoribosyltransferase | OXL26833.1 |
| NEXU01000018 | 18599 | 18240 | 360 | branched-chain amino acid transport | OXL26834.1 |
| NEXU01000018 | 19320 | 18589 | 732 | branched-chain amino acid ABC transporter | OXL26835.1 |
| NEXU01000018 | 20813 | 19395 | 1419 | MATE family efflux transporter | OXL26836.1 |
| NEXU01000018 | 22274 | 21030 | 1245 | protein translocase subunit SecF | OXL26837.1 |
| NEXU01000018 | 24161 | 22284 | 1878 | protein translocase subunit SecD | OXL26838.1 |
| NEXU01000018 | 24715 | 24437 | 279 | preprotein translocase subunit YajC | OXL26839.1 |
| NEXU01000018 | 25393 | 26673 | 1281 | hypothetical protein | OXL26840.1 |
| NEXU01000018 | 27022 | 27516 | 495 | hypothetical protein | OXL26841.1 |
| NEXU01000018 | 27778 | 30855 | 3078 | lytic transglycosylase | OXL26842.1 |
| NEXU01000018 | 32201 | 30993 | 1209 | lytic transglycosylase | OXL26843.1 |
| NEXU01000018 | 33987 | 33154 | 834 | Nif3-like dinuclear metal center hexameric protein | OXL26844.1 |
| NEXU01000018 | 34277 | 35605 | 1329 | serine protease | OXL26845.1 |
| NEXU01000018 | 36120 | 35854 | 267 | (2Fe-2S)-binding protein | OXL26871.1 |
| NEXU01000018 | 37330 | 36197 | 1134 | ribonucleotide-diphosphate reductase subunit beta | OXL26846.1 |
| NEXU01000018 | 37794 | 37438 | 357 | hypothetical protein | OXL26872.1 |
| NEXU01000018 | 38122 | 37835 | 288 | addiction module toxin RelE | OXL26847.1 |
| NEXU01000018 | 38363 | 38112 | 252 | antitoxin | OXL26848.1 |
| NEXU01000018 | 39840 | 38500 | 1341 | hypothetical protein | OXL26849.1 |
| NEXU01000018 | 40487 | 39837 | 651 | hypothetical protein | OXL26850.1 |
| NEXU01000018 | 42874 | 40598 | 2277 | ribonucleoside-diphosphate reductase subunit alpha | OXL26851.1 |
| NEXU01000018 | 43829 | 43404 | 426 | DUF493 domain-containing protein | OXL26852.1 |
| NEXU01000018 | 45921 | 44008 | 1914 | RNA polymerase sigma factor RpoD | OXL26853.1 |
| NEXU01000018 | 48597 | 46450 | 2148 | DNA primase | OXL26854.1 |
| NEXU01000018 | 49856 | 48771 | 1086 | outer membrane protein assembly factor BamD | OXL26855.1 |
| NEXU01000018 | 50648 | 51895 | 1248 | pseudouridine synthase | OXL26873.1 |
| NEXU01000018 | 51957 | 52925 | 969 | multicopper polyphenol oxidase | OXL26856.1 |
| NEXU01000018 | 53099 | 53728 | 630 | flavodoxin | OXL26857.1 |
| NEXU01000018 | 54619 | 56073 | 1455 | cytochrome C oxidase Cbb3 | OXL26858.1 |
| NEXU01000018 | 56289 | 57890 | 1602 | flavin monoamine oxidase | OXL26859.1 |
| NEXU01000018 | 57937 | 58581 | 645 | cytochrome C oxidase Cbb3 | OXL26860.1 |
| NEXU01000018 | 60073 | 58781 | 1293 | translocation protein TolB | OXL26861.1 |
| NEXU01000018 | 61221 | 60211 | 1011 | energy transducer TonB | OXL26862.1 |
| NEXU01000018 | 61800 | 61342 | 459 | protein TolR | OXL26863.1 |
| NEXU01000018 | 62528 | 61797 | 732 | protein TolQ | OXL26864.1 |
| NEXU01000018 | 63041 | 64336 | 1296 | guanine permease | OXL26865.1 |
| NEXU01000018 | 65074 | 68331 | 3258 | bifunctional proline dehydrogenase/L-glutamate gamma-semialdehyde dehydrogenase | OXL26866.1 |
| NEXU01000018 | 68343 | 69035 | 693 | 1-pyrroline-5-carboxylate dehydrogenase | OXL26867.1 |
| NEXU01000018 | 69801 | 71495 | 1695 | molybdopterin dehydrogenase | OXL26868.1 |
| NEXU01000018 | 71744 | >73854 | N/D | xanthine dehydrogenase molybdopterin binding subunit | OXL26869.1 |
| NEXU01000019 | 38 | 808 | 771 | hypothetical protein |  |
| NEXU01000019 | 825 | 1838 | 1014 | xanthine dehydrogenase accessory protein XdhC | OXL26712.1 |
| NEXU01000019 | 2061 | 3503 | 1443 | guanine deaminase | OXL26701.1 |
| NEXU01000019 | 4874 | 3702 | 1173 | ribosomal oxygenase | OXL26702.1 |
| NEXU01000019 | 5024 | 5209 | 186 | hypothetical protein | OXL26703.1 |
| NEXU01000019 | 5849 | 5211 | 639 | hypothetical protein | OXL26704.1 |
| NEXU01000019 | 7298 | 5895 | 1404 | adenylosuccinate lyase | OXL26705.1 |
| NEXU01000019 | 7577 | 7362 | 216 | hypothetical protein | OXL26706.1 |
| NEXU01000019 | 8827 | 7601 | 1227 | tRNA 2-thiouridine(34) synthase MnmA | OXL26707.1 |
| NEXU01000019 | 9089 | 9589 | 501 | 2-C-methyl-D-erythritol 2,4-cyclodiphosphate synthase | OXL26708.1 |
| NEXU01000019 | 9780 | 10130 | 351 | glutaredoxin | OXL26709.1 |
| NEXU01000019 | 10317 | 10847 | 531 | hypothetical protein | OXL26710.1 |
| NEXU01000019 | 11088 | 12983 | 1896 | tRNA uridine-5-carboxymethylaminomethyl(34) synthesis enzyme MnmG | OXL26711.1 |
| NEXU01000019 | 13183 | 13470 | 288 | malonate transporter |  |
| NEXU01000020 | 27 | 950 | 924 | malonate transporter | OXL26649.1 |
| NEXU01000020 | 1036 | 2064 | 1029 | ornithine cyclodeaminase | OXL26600.1 |
| NEXU01000020 | 3435 | 2434 | 1002 | alkane 1-monooxygenase | OXL26601.1 |
| NEXU01000020 | 3841 | 4047 | 207 | molybdenum-pterin-binding protein | OXL26602.1 |
| NEXU01000020 | 5108 | 4179 | 930 | CysB family transcriptional regulator | OXL26603.1 |
| NEXU01000020 | 5591 | 7282 | 1692 | MFS transporter | OXL26604.1 |
| NEXU01000020 | 7331 | 8206 | 876 | protein-(glutamine-N5) methyltransferase, release factor-specific | OXL26605.1 |
| NEXU01000020 | 8284 | 9081 | 798 | molybdopterin biosynthesis protein | OXL26606.1 |
| NEXU01000020 | 9157 | 10512 | 1356 | kinase | OXL26607.1 |
| NEXU01000020 | 10619 | 11878 | 1260 | MFS transporter | OXL26608.1 |
| NEXU01000020 | 12363 | 11875 | 489 | AsnC family transcriptional regulator | OXL26609.1 |
| NEXU01000020 | 12607 | 14400 | 1794 | 2-isopropylmalate synthase | OXL26610.1 |
| NEXU01000020 | 14646 | 15260 | 615 | hypothetical protein | OXL26611.1 |
| NEXU01000020 | 16733 | 15372 | 1362 | acetyl-CoA carboxylase biotin carboxylase subunit | OXL26612.1 |
| NEXU01000020 | 17315 | 16887 | 429 | acetyl-CoA carboxylase, biotin carboxyl carrier protein | OXL26613.1 |
| NEXU01000020 | 18946 | 17579 | 1368 | MATE family efflux transporter | OXL26614.1 |
| NEXU01000020 | 19786 | 19292 | 495 | EVE domain-containing protein | OXL26615.1 |
| NEXU01000020 | 20016 | 21323 | 1308 | YggW family oxidoreductase | OXL26616.1 |
| NEXU01000020 | 21601 | 21461 | 141 | entericidin | OXL26617.1 |
| NEXU01000020 | 21859 | 22422 | 564 | tRNA (uridine(34)/cytosine(34)/5-carboxymethylaminomethyluridine(34)-2'-O)- methyltransferase TrmL | OXL26618.1 |
| NEXU01000020 | 22879 | 22523 | 357 | sulfite reductase | OXL26619.1 |
| NEXU01000020 | 23214 | 22876 | 339 | hypothetical protein | OXL26620.1 |
| NEXU01000020 | 23630 | 23307 | 324 | hypothetical protein | OXL26621.1 |
| NEXU01000020 | 24139 | 23699 | 441 | multidrug transporter | OXL26622.1 |
| NEXU01000020 | 25434 | 24388 | 1047 | NADP-dependent oxidoreductase | OXL26623.1 |
| NEXU01000020 | 25798 | 28647 | 2850 | bifunctional glutamine synthetase adenylyltransferase/deadenyltransferase | OXL26624.1 |
| NEXU01000020 | 28892 | 29821 | 930 | branched-chain amino acid aminotransferase | OXL26625.1 |
| NEXU01000020 | 30851 | 29919 | 933 | hypothetical protein | OXL26626.1 |
| NEXU01000020 | 31297 | 30848 | 450 | hypothetical protein | OXL26627.1 |
| NEXU01000020 | 32366 | 31290 | 1077 | glycosyltransferase | OXL26628.1 |
| NEXU01000020 | 33982 | 32429 | 1554 | hypothetical protein | OXL26629.1 |
| NEXU01000020 | 34867 | 34295 | 573 | dTDP-4-dehydrorhamnose 3,5-epimerase | OXL26630.1 |
| NEXU01000020 | 35785 | 34895 | 891 | glucose-1-phosphate thymidylyltransferase | OXL26631.1 |
| NEXU01000020 | 36687 | 35782 | 906 | dTDP-4-dehydrorhamnose reductase | OXL26632.1 |
| NEXU01000020 | 37787 | 36723 | 1065 | dTDP-glucose 4,6-dehydratase | OXL26633.1 |
| NEXU01000020 | 37909 | 38778 | 870 | glycosyltransferase, group 2 family protein | OXL26634.1 |
| NEXU01000020 | 39623 | 38766 | 858 | hypothetical protein | OXL26635.1 |
| NEXU01000020 | 40668 | 39685 | 984 | hypothetical protein | OXL26636.1 |
| NEXU01000020 | 41777 | 40671 | 1107 | aminotransferase | OXL26637.1 |
| NEXU01000020 | 42235 | 41774 | 462 | dTDP-6-deoxy-3,4-keto-hexulose isomerase | OXL26638.1 |
| NEXU01000020 | 42633 | 42232 | 402 | dTDP-6-deoxy-3,4-keto-hexulose isomerase | OXL26639.1 |
| NEXU01000020 | 43564 | 42644 | 921 | hypothetical protein | OXL26640.1 |
| NEXU01000020 | 44445 | 43594 | 852 | hypothetical protein | OXL26641.1 |
| NEXU01000020 | 45200 | 44451 | 750 | hypothetical protein | OXL26642.1 |
| NEXU01000020 | 45478 | 46599 | 1122 | glycosyl transferase | OXL26643.1 |
| NEXU01000020 | 47434 | 46592 | 843 | hypothetical protein | OXL26650.1 |
| NEXU01000020 | 48303 | 47503 | 801 | glycosyltransferase | OXL26644.1 |
| NEXU01000020 | 49350 | 48424 | 927 | lipid A biosynthesis acyltransferase | OXL26645.1 |
| NEXU01000020 | 51244 | 49418 | 1827 | aspartate--tRNA ligase | OXL26651.1 |
| NEXU01000020 | 51611 | 52585 | 975 | protease SohB | OXL26646.1 |
| NEXU01000020 | 52841 | 54091 | 1251 | acyl-CoA dehydrogenase | OXL26647.1 |
| NEXU01000020 | 54280 | >55035 | N/D | phosphotransferase family protein | OXL26648.1 |
| NEXU01000021 | 5 | 576 | 572 | phosphotransferase family protein |  |
| NEXU01000021 | 657 | 1415 | 759 | histidine phosphatase family protein | OXL26503.1 |
| NEXU01000021 | 1594 | 2463 | 870 | short chain dehydrogenase | OXL26504.1 |
| NEXU01000021 | 4281 | 2581 | 1701 | long-chain fatty acid--CoA ligase | OXL26505.1 |
| NEXU01000021 | 6646 | 4949 | 1698 | long-chain fatty acid--CoA ligase | OXL26506.1 |
| NEXU01000021 | 7688 | 7107 | 582 | thioesterase | OXL26507.1 |
| NEXU01000021 | 8044 | 8868 | 825 | exodeoxyribonuclease III | OXL26508.1 |
| NEXU01000021 | 8940 | 10163 | 1224 | bifunctional ornithine acetyltransferase/N-acetylglutamate synthase | OXL26509.1 |
| NEXU01000021 | 10581 | 10979 | 399 | hypothetical protein | OXL26510.1 |
| NEXU01000021 | 12542 | 11109 | 1434 | L-serine ammonia-lyase | OXL26511.1 |
| NEXU01000021 | 13276 | 14697 | 1422 | pyrimidine utilization transport protein G | OXL26512.1 |
| NEXU01000021 | 15750 | 14818 | 933 | EamA family transporter | OXL26513.1 |
| NEXU01000021 | 16210 | 17268 | 1059 | hypothetical protein | OXL26514.1 |
| NEXU01000021 | 18456 | 17416 | 1041 | alpha/beta hydrolase | OXL26515.1 |
| NEXU01000021 | 18649 | 18485 | 165 | rubredoxin | OXL26516.1 |
| NEXU01000021 | 18990 | 19559 | 570 | adenine phosphoribosyltransferase | OXL26517.1 |
| NEXU01000021 | 20129 | 19653 | 477 | hypothetical protein | OXL26518.1 |
| NEXU01000021 | 21251 | 20310 | 942 | transaldolase | OXL26519.1 |
| NEXU01000021 | 21912 | 23093 | 1182 | chorismate mutase | OXL26520.1 |
| NEXU01000021 | 23179 | 24321 | 1143 | histidinol-phosphate transaminase | OXL26521.1 |
| NEXU01000021 | 24405 | 26768 | 2364 | bifunctional prephenate dehydrogenase/3-phosphoshikimate 1-carboxyvinyltransferase | OXL26522.1 |
| NEXU01000021 | 27035 | 27994 | 960 | protein RarD | OXL26523.1 |
| NEXU01000021 | 28040 | 28435 | 396 | dihydroneopterin aldolase | OXL26524.1 |
| NEXU01000021 | 28425 | 28958 | 534 | hypothetical protein | OXL26525.1 |
| NEXU01000021 | 29024 | 29563 | 540 | hypothetical protein | OXL26526.1 |
| NEXU01000021 | 29669 | 30034 | 366 | MarR family transcriptional regulator | OXL26527.1 |
| NEXU01000021 | 31101 | 30163 | 939 | serine O-acetyltransferase | OXL26528.1 |
| NEXU01000021 | 31696 | 31253 | 444 | hypothetical protein | OXL26529.1 |
| NEXU01000021 | 32791 | 31880 | 912 | rRNA methyltransferase | OXL26530.1 |
| NEXU01000021 | 33171 | 32788 | 384 | histidine kinase | OXL26531.1 |
| NEXU01000021 | >36454 | 33264 | N/D | DNA polymerase III subunit alpha | OXL26532.1 |
| NEXU01000022 | 683 | 6 | 678 | DNA polymerase III subunit alpha |  |
| NEXU01000022 | 2907 | 991 | 1917 | gamma-glutamyltransferase | OXL26439.1 |
| NEXU01000022 | 3135 | 3569 | 435 | hypothetical protein | OXL26433.1 |
| NEXU01000022 | 3783 | 5849 | 2067 | hypothetical protein | OXL26434.1 |
| NEXU01000022 | 5937 | 7709 | 1773 | hypothetical protein | OXL26435.1 |
| NEXU01000022 | 8401 | 7871 | 531 | hypothetical protein | OXL26436.1 |
| NEXU01000022 | 8574 | 9260 | 687 | DNA-binding response regulator | OXL26437.1 |
| NEXU01000022 | 9278 | 10924 | 1647 | two-component sensor histidine kinase | OXL26440.1 |
| NEXU01000022 | 11233 | >12930 | N/D | hypothetical protein | OXL26438.1 |
| NEXU01000023 | 2 | >594 | N/D | hypothetical protein | OXL26414.1 |
| NEXU01000024 | <1 | 2497 | N/D | hypothetical protein | OXL26176.1 |
| NEXU01000024 | 2998 | 4524 | 1527 | propionyl-CoA--succinate CoA transferase | OXL26177.1 |
| NEXU01000024 | 5417 | 4641 | 777 | hypothetical protein | OXL26178.1 |
| NEXU01000024 | 6360 | 5518 | 843 | hypothetical protein | OXL26179.1 |
| NEXU01000024 | 8006 | 6573 | 1434 | nitrite reductase, copper-containing | OXL26196.1 |
| NEXU01000024 | 9339 | 8635 | 705 | hypothetical protein | OXL26180.1 |
| NEXU01000024 | 11011 | 9548 | 1464 | hypothetical protein | OXL26181.1 |
| NEXU01000024 | 12858 | 11167 | 1692 | chromosome condensation regulator RCC1 | OXL26182.1 |
| NEXU01000024 | 13339 | 14028 | 690 | glutathione S-transferase | OXL26183.1 |
| NEXU01000024 | 14069 | 14635 | 567 | tellurite resistance methyltransferase TehB | OXL26184.1 |
| NEXU01000024 | 15720 | 14803 | 918 | LysR family transcriptional regulator | OXL26185.1 |
| NEXU01000024 | 15895 | 16284 | 390 | reactive intermediate/imine deaminase | OXL26186.1 |
| NEXU01000024 | 16381 | 17844 | 1464 | aldehyde dehydrogenase family protein | OXL26187.1 |
| NEXU01000024 | 17954 | 18940 | 987 | peptidase M19 | OXL26188.1 |
| NEXU01000024 | 19056 | 20738 | 1683 | Choline-glycine betaine transporter, BCCT family | OXL26189.1 |
| NEXU01000024 | 20842 | 22569 | 1728 | hypothetical protein | OXL26190.1 |
| NEXU01000024 | 22583 | 23308 | 726 | hypothetical protein | OXL26191.1 |
| NEXU01000024 | 23458 | 24384 | 927 | hypothetical protein | OXL26192.1 |
| NEXU01000024 | 25709 | 24957 | 753 | DUF305 domain-containing protein | OXL26193.1 |
| NEXU01000024 | 26338 | 25853 | 486 | DUF305 domain-containing protein | OXL26194.1 |
| NEXU01000024 | 27001 | 26384 | 618 | ATP-binding protein | OXL26195.1 |
| NEXU01000024 | 29647 | 27173 | 2475 | copper-translocating P-type ATPase | OXL26197.1 |
| NEXU01000024 | 29977 | 30523 | 547 | DNA-binding response regulator |  |
| NEXU01000025 | 41 | 1390 | 1350 | two-component sensor histidine kinase | OXL26126.1 |
| NEXU01000025 | 1756 | 3120 | 1365 | hypothetical protein | OXL26115.1 |
| NEXU01000025 | 3615 | 4373 | 759 | tRNA pseudouridine(65) synthase TruC | OXL26127.1 |
| NEXU01000025 | 5847 | 4813 | 1035 | AraC family transcriptional regulator | OXL26116.1 |
| NEXU01000025 | 6034 | 7467 | 1434 | coniferyl-aldehyde dehydrogenase | OXL26117.1 |
| NEXU01000025 | 7617 | 8927 | 1311 | alcohol dehydrogenase | OXL26118.1 |
| NEXU01000025 | 10113 | 9037 | 1077 | AraC family transcriptional regulator | OXL26119.1 |
| NEXU01000025 | 10269 | 10589 | 321 | 2Fe-2S ferredoxin | OXL26120.1 |
| NEXU01000025 | 10717 | 12177 | 1461 | cytochrome P450 | OXL26121.1 |
| NEXU01000025 | 12815 | 12405 | 411 | Cd(II)/Pb(II)-responsive transcriptional regulator | OXL26122.1 |
| NEXU01000025 | 12985 | 15150 | 2166 | copper-translocating P-type ATPase | OXL26123.1 |
| NEXU01000025 | 16745 | 15402 | 1344 | ornithine monooxygenase | OXL26124.1 |
| NEXU01000025 | 17193 | 17387 | 195 | hypothetical protein | OXL26125.1 |
| NEXU01000026 | 33 | 377 | 345 | hypothetical protein |  |
| NEXU01000027 | 613 | 92 | 522 | peptide-methionine (S)-S-oxide reductase | OXL25887.1 |
| NEXU01000027 | 829 | 1629 | 801 | carboxy-S-adenosyl-L-methionine synthase CmoA | OXL25888.1 |
| NEXU01000027 | 1714 | 2763 | 1050 | tRNA 5-methoxyuridine(34)/uridine 5-oxyacetic acid(34) synthase CmoB | OXL25889.1 |
| NEXU01000027 | 2807 | 3991 | 1185 | hypothetical protein | OXL25890.1 |
| NEXU01000027 | 4234 | 4016 | 219 | hypothetical protein | OXL25891.1 |
| NEXU01000027 | 4674 | 6503 | 1830 | arginine--tRNA ligase | OXL25892.1 |
| NEXU01000027 | 6573 | 7397 | 825 | SPOR domain-containing protein | OXL25893.1 |
| NEXU01000027 | 7503 | 8597 | 1095 | toxin regulator PfoR | OXL25894.1 |
| NEXU01000027 | 9751 | 8765 | 987 | cytochrome C | OXL25895.1 |
| NEXU01000027 | 11231 | 10212 | 1020 | short-chain dehydrogenase | OXL25896.1 |
| NEXU01000027 | 12788 | 11475 | 1314 | ATP-dependent protease ATP-binding subunit ClpX | OXL25897.1 |
| NEXU01000027 | 13694 | 12999 | 696 | ATP-dependent Clp endopeptidase, proteolytic subunit ClpP | OXL25898.1 |
| NEXU01000027 | 15494 | 14151 | 1344 | trigger factor | OXL25899.1 |
| NEXU01000027 | 16515 | 15862 | 654 | non-canonical purine NTP pyrophosphatase, RdgB/HAM1 family | OXL25900.1 |
| NEXU01000027 | 17063 | 17692 | 630 | superoxide dismutase [Fe] | OXL25901.1 |
| NEXU01000027 | 18515 | 17775 | 741 | hypothetical protein | OXL25902.1 |
| NEXU01000027 | 19403 | 18570 | 834 | hypothetical protein | OXL25928.1 |
| NEXU01000027 | 19997 | 19500 | 498 | diacylglycerol kinase | OXL25903.1 |
| NEXU01000027 | 21079 | 20051 | 1029 | farnesyl-diphosphate synthase | OXL25904.1 |
| NEXU01000027 | 21817 | 23976 | 2160 | fatty acid oxidation complex subunit alpha FadB | OXL25905.1 |
| NEXU01000027 | 24120 | 25292 | 1173 | acetyl-CoA C-acyltransferase FadA | OXL25906.1 |
| NEXU01000027 | 25497 | 27209 | 1713 | hypothetical protein | OXL25907.1 |
| NEXU01000027 | 27412 | 27987 | 576 | hydrolase | OXL25908.1 |
| NEXU01000027 | 28173 | 28784 | 612 | phosphohydrolase | OXL25909.1 |
| NEXU01000027 | 28901 | 29506 | 606 | DUF1287 domain-containing protein | OXL25910.1 |
| NEXU01000027 | 30266 | 29529 | 738 | phosphate transport system regulatory protein PhoU | OXL25911.1 |
| NEXU01000027 | 31202 | 30423 | 780 | phosphate ABC transporter ATP-binding protein | OXL25929.1 |
| NEXU01000027 | 32348 | 31434 | 915 | phosphate ABC transporter, permease protein PstA | OXL25912.1 |
| NEXU01000027 | 33385 | 32435 | 951 | phosphate ABC transporter permease subunit PstC | OXL25913.1 |
| NEXU01000027 | 34723 | 33566 | 1158 | phosphate ABC transporter substrate-binding protein PstS | OXL25914.1 |
| NEXU01000027 | 35852 | 35019 | 834 | phosphatidylserine decarboxylase | OXL25915.1 |
| NEXU01000027 | 36037 | 36492 | 456 | D-tyrosyl-tRNA(Tyr) deacylase | OXL25916.1 |
| NEXU01000027 | 36641 | 37345 | 705 | phosphate regulon transcriptional regulatory protein PhoB | OXL25917.1 |
| NEXU01000027 | 37441 | 38538 | 1098 | phosphate regulon sensor histidine kinase PhoR | OXL25918.1 |
| NEXU01000027 | 39285 | 38686 | 600 | peptidase M23 | OXL25919.1 |
| NEXU01000027 | 39507 | 40202 | 696 | exonuclease | OXL25920.1 |
| NEXU01000027 | 41133 | 40237 | 897 | alpha/beta hydrolase | OXL25921.1 |
| NEXU01000027 | 41477 | 42154 | 678 | Rossman fold protein, TIGR00730 family | OXL25922.1 |
| NEXU01000027 | 43896 | 42319 | 1578 | FAD-dependent oxidoreductase | OXL25923.1 |
| NEXU01000027 | 44430 | 44867 | 438 | hypothetical protein | OXL25924.1 |
| NEXU01000027 | 45671 | 45048 | 624 | polyisoprenoid-binding protein | OXL25925.1 |
| NEXU01000027 | 46165 | 46446 | 282 | 50S ribosomal protein L31 type B | OXL25926.1 |
| NEXU01000027 | >47206 | 46586 | N/D | G-D-S-L lipolytic protein | OXL25927.1 |
| NEXU01000028 | 659 | 22 | 638 | G-D-S-L lipolytic protein |  |
| NEXU01000028 | 2050 | 653 | 1398 | nicotinate phosphoribosyltransferase | OXL25864.1 |
| NEXU01000028 | 3291 | 2194 | 1098 | ADP-ribose pyrophosphatase | OXL25865.1 |
| NEXU01000028 | 4470 | 3460 | 1011 | alpha/beta hydrolase | OXL25866.1 |
| NEXU01000028 | 5001 | 4654 | 348 | alkylhydroperoxidase | OXL25867.1 |
| NEXU01000028 | 5737 | 6420 | 684 | Fe/S biogenesis protein NfuA | OXL25868.1 |
| NEXU01000028 | 6726 | 8036 | 1311 | O-acetylhomoserine aminocarboxypropyltransferase | OXL25869.1 |
| NEXU01000028 | 9191 | 8172 | 1020 | adenosine kinase | OXL25870.1 |
| NEXU01000028 | 10217 | 9246 | 972 | 16S rRNA (cytidine(1402)-2'-O)-methyltransferase | OXL25871.1 |
| NEXU01000028 | 10384 | 10947 | 564 | YraN family protein | OXL25872.1 |
| NEXU01000028 | 11180 | 12016 | 837 | transporter | OXL25873.1 |
| NEXU01000028 | 12317 | 13267 | 951 | esterase | OXL25874.1 |
| NEXU01000028 | 13396 | 13647 | 252 | hypothetical protein | OXL25875.1 |
| NEXU01000028 | 13760 | 15028 | 1269 | UDP-N-acetylglucosamine 1-carboxyvinyltransferase | OXL25876.1 |
| NEXU01000028 | 15172 | 15867 | 696 | ATP phosphoribosyltransferase | OXL25877.1 |
| NEXU01000028 | 16133 | 17428 | 1296 | histidinol dehydrogenase | OXL25878.1 |
| NEXU01000028 | 17519 | 18652 | 1134 | histidinol-phosphate transaminase | OXL25879.1 |
| NEXU01000028 | 20514 | 18793 | 1722 | aminodeoxychorismate synthase component I | OXL25880.1 |
| NEXU01000028 | 21753 | 20518 | 1236 | beta-ketoacyl-[acyl-carrier-protein] synthase I | OXL25881.1 |
| NEXU01000029 | <1 | 193 | N/D | hypothetical protein | OXL25801.1 |
| NEXU01000029 | 229 | 1524 | 1296 | hypothetical protein | OXL25802.1 |
| NEXU01000029 | 1975 | 2349 | 375 | 30S ribosomal protein S12 | OXL25803.1 |
| NEXU01000029 | 2558 | 3031 | 474 | 30S ribosomal protein S7 | OXL25804.1 |
| NEXU01000029 | 3459 | 5585 | 2127 | elongation factor G | OXL25805.1 |
| NEXU01000029 | 5808 | >6566 | N/D | elongation factor Tu | OXL25806.1 |
| NEXU01000030 | 244 | 319 | 76 | tRNA-Trp |  |
| NEXU01000030 | 371 | 856 | 486 | preprotein translocase subunit SecE | OXL25757.1 |
| NEXU01000030 | 942 | 1472 | 531 | transcription termination/antitermination protein NusG | OXL25758.1 |
| NEXU01000030 | 1568 | 1999 | 432 | 50S ribosomal protein L11 | OXL25759.1 |
| NEXU01000030 | 1999 | 2700 | 702 | 50S ribosomal protein L1 | OXL25760.1 |
| NEXU01000030 | 3132 | 3659 | 528 | 50S ribosomal protein L10 | OXL25761.1 |
| NEXU01000030 | 3786 | 4157 | 372 | 50S ribosomal protein L7/L12 | OXL25762.1 |
| NEXU01000030 | 4945 | 9063 | 4119 | DNA-directed RNA polymerase subunit beta | OXL25763.1 |
| NEXU01000030 | 9215 | 13435 | 4221 | DNA-directed RNA polymerase subunit beta' | OXL25764.1 |
| NEXU01000030 | 13642 | 14169 | 528 | hypothetical protein | OXL25765.1 |
| NEXU01000030 | 16954 | 14252 | 2703 | penicillin-binding protein 1A | OXL25766.1 |
| NEXU01000030 | 17148 | 18203 | 1056 | pilus assembly protein PilM | OXL25767.1 |
| NEXU01000030 | 18203 | 18856 | 654 | pilus assembly protein PilS | OXL25768.1 |
| NEXU01000030 | 18853 | 19548 | 696 | hypothetical protein | OXL25769.1 |
| NEXU01000030 | 19548 | 20081 | 534 | pilus assembly protein PilQ | OXL25770.1 |
| NEXU01000030 | 20109 | 22475 | 2367 | pilus assembly protein PilQ | OXL25771.1 |
| NEXU01000030 | 22651 | 23211 | 561 | shikimate kinase I | OXL25772.1 |
| NEXU01000030 | 23293 | 24426 | 1134 | 3-dehydroquinate synthase | OXL25773.1 |
| NEXU01000030 | 24543 | 25670 | 1128 | hypothetical protein | OXL25774.1 |
| NEXU01000030 | 26364 | 30827 | 4464 | glutamate synthase large subunit | OXL25775.1 |
| NEXU01000030 | 30999 | 32420 | 1422 | glutamate synthase small subunit | OXL25776.1 |
| NEXU01000030 | 32619 | 33854 | 1236 | lantibiotic ABC transporter permease | OXL25777.1 |
| NEXU01000030 | 35091 | 33967 | 1125 | spermidine/putrescine ABC transporter substrate-binding protein PotD | OXL25778.1 |
| NEXU01000030 | 35376 | 36173 | 798 | 3-oxoacyl-ACP reductase | OXL25779.1 |
| NEXU01000030 | 36226 | 36492 | 267 | hypothetical protein | OXL25780.1 |
| NEXU01000030 | 36549 | 37343 | 795 | hypothetical protein | OXL25781.1 |
| NEXU01000030 | 38210 | 37416 | 795 | HAD family hydrolase | OXL25782.1 |
| NEXU01000030 | 38993 | 38352 | 642 | hypothetical protein | OXL25783.1 |
| NEXU01000031 | 1880 | <1 | N/D | DNA topoisomerase IV subunit A | OXL25652.1 |
| NEXU01000031 | 2594 | 4324 | 1731 | long-chain fatty acid--CoA ligase | OXL25653.1 |
| NEXU01000031 | 4799 | 6583 | 1785 | long-chain fatty acid--CoA ligase | OXL25654.1 |
| NEXU01000031 | 6814 | 7530 | 717 | ribonuclease PH | OXL25655.1 |
| NEXU01000031 | 7605 | 8096 | 492 | hypothetical protein | OXL25656.1 |
| NEXU01000031 | 8335 | 9096 | 762 | hypothetical protein | OXL25657.1 |
| NEXU01000031 | 9797 | 9363 | 435 | TIGR00701 family protein | OXL25658.1 |
| NEXU01000031 | 11061 | 9874 | 1188 | biotin synthase BioB | OXL25659.1 |
| NEXU01000031 | 11792 | 11286 | 507 | hypothetical protein | OXL25660.1 |
| NEXU01000031 | 12360 | 11971 | 390 | hypothetical protein | OXL25661.1 |
| NEXU01000031 | 13614 | 12574 | 1041 | DUF368 domain-containing protein | OXL25662.1 |
| NEXU01000031 | 14955 | 13768 | 1188 | phosphoserine phosphatase SerB | OXL25663.1 |
| NEXU01000031 | 15392 | 16918 | 1527 | hypothetical protein | OXL25664.1 |
| NEXU01000031 | 17113 | 17349 | 237 | hypothetical protein | OXL25665.1 |
| NEXU01000031 | 17663 | 18220 | 558 | YgfB and YecA protein | OXL25666.1 |
| NEXU01000031 | 18281 | 18745 | 465 | hypothetical protein | OXL25671.1 |
| NEXU01000031 | 18844 | 19314 | 471 | metal-binding protein | OXL25667.1 |
| NEXU01000031 | 19426 | 19620 | 195 | hypothetical protein | OXL25668.1 |
| NEXU01000031 | 20787 | 19669 | 1119 | formate transporter | OXL25669.1 |
| NEXU01000031 | 20984 | 21523 | 540 | hypothetical protein | OXL25670.1 |
| NEXU01000032 | 43 | 3015 | 2973 | valine--tRNA ligase | OXL25539.1 |
| NEXU01000032 | 3293 | 4747 | 1455 | sodium:proton antiporter | OXL25540.1 |
| NEXU01000032 | 4805 | 5203 | 399 | hypothetical protein | OXL25541.1 |
| NEXU01000032 | 5607 | 6083 | 477 | hypothetical protein | OXL25542.1 |
| NEXU01000032 | 6452 | 6874 | 423 | hypothetical protein | OXL25543.1 |
| NEXU01000032 | 7119 | 7328 | 210 | cold-shock protein CspC | OXL25544.1 |
| NEXU01000032 | 8636 | 7554 | 1083 | hypothetical protein | OXL25545.1 |
| NEXU01000032 | 8892 | 9839 | 948 | LysR family transcriptional regulator | OXL25546.1 |
| NEXU01000032 | 10401 | 9940 | 462 | universal stress protein | OXL25547.1 |
| NEXU01000032 | 10791 | 13856 | 3066 | peptidase M16 | OXL25548.1 |
| NEXU01000032 | 15432 | 14113 | 1320 | 6-aminohexanoate hydrolase | OXL25549.1 |
| NEXU01000032 | 16183 | 15620 | 564 | NUDIX hydrolase | OXL25550.1 |
| NEXU01000032 | 17015 | 16531 | 485 | HPP family protein |  |
| NEXU01000032 | 17241 | 17978 | 738 | coenzyme A pyrophosphatase | OXL25551.1 |
| NEXU01000033 | 1545 | 160 | 1386 | hypothetical protein | OXL25506.1 |
| NEXU01000033 | 2225 | 1629 | 597 | hypothetical protein | OXL25507.1 |
| NEXU01000033 | 3481 | 2279 | 1203 | Na+/H+ antiporter NhaA | OXL25508.1 |
| NEXU01000033 | 3973 | 4728 | 756 | hypothetical protein | OXL25509.1 |
| NEXU01000033 | 6206 | 4833 | 1374 | dicarboxylate/amino acid:cation symporter | OXL25510.1 |
| NEXU01000033 | 9534 | 6607 | 2928 | acyl-CoA dehydrogenase | OXL25511.1 |
| NEXU01000033 | 10599 | 9916 | 684 | hypothetical protein | OXL25512.1 |
| NEXU01000033 | 10728 | 12113 | 1386 | phosphopantothenoylcysteine decarboxylase | OXL25513.1 |
| NEXU01000033 | 12252 | 13034 | 783 | hypothetical protein | OXL25525.1 |
| NEXU01000033 | 13331 | 13125 | 207 | hypothetical protein | OXL25514.1 |
| NEXU01000033 | 14314 | 13397 | 918 | histone deacetylase | OXL25515.1 |
| NEXU01000033 | 15258 | 14437 | 822 | hypothetical protein | OXL25516.1 |
| NEXU01000033 | 16390 | 15338 | 1053 | hypothetical protein | OXL25526.1 |
| NEXU01000033 | 16509 | 17138 | 630 | heme ABC exporter ATP-binding protein CcmA | OXL25517.1 |
| NEXU01000033 | 17265 | 17930 | 666 | ABC transporter permease | OXL25527.1 |
| NEXU01000033 | 18130 | 18915 | 786 | heme ABC transporter permease | OXL25518.1 |
| NEXU01000033 | 18951 | 19175 | 225 | heme exporter protein CcmD | OXL25519.1 |
| NEXU01000033 | 19415 | 19915 | 501 | cytochrome c biogenesis protein CcmE | OXL25520.1 |
| NEXU01000033 | 20887 | 20039 | 849 | aldo/keto reductase | OXL25521.1 |
| NEXU01000033 | 22159 | 21200 | 960 | hypothetical protein | OXL25522.1 |
| NEXU01000033 | 23089 | 22403 | 687 | methionine ABC transporter ATP-binding protein | OXL25523.1 |
| NEXU01000033 | 23879 | 23109 | 771 | ABC transporter | OXL25524.1 |
| NEXU01000034 | 231 | 5 | 227 | ABC transporter |  |
| NEXU01000034 | 2646 | 541 | 2106 | HAMP domain-containing histidine kinase | OXL25469.1 |
| NEXU01000034 | 3525 | 2803 | 723 | DNA-binding response regulator | OXL25470.1 |
| NEXU01000035 | 1592 | 402 | 1191 | hemolysin D | OXL25463.1 |
| NEXU01000035 | 2401 | 2778 | 378 | response regulator | OXL25456.1 |
| NEXU01000035 | 2879 | 3241 | 363 | two-component system response regulator | OXL25457.1 |
| NEXU01000035 | 3245 | 3775 | 531 | chemotaxis protein CheW | OXL25458.1 |
| NEXU01000035 | 3969 | 5390 | 1422 | methyl-accepting chemotaxis protein | OXL25459.1 |
| NEXU01000035 | 5492 | 6472 | 981 | chemotaxis protein CheR | OXL25460.1 |
| NEXU01000035 | 6503 | 13465 | 6963 | hybrid sensor histidine kinase/response regulator | OXL25461.1 |
| NEXU01000035 | 13458 | >14259 | N/D | chemotaxis protein CheB | OXL25462.1 |
| NEXU01000036 | 2 | 412 | 411 | chemotaxis protein CheB |  |
| NEXU01000036 | 409 | 912 | 504 | hypothetical protein | OXL25400.1 |
| NEXU01000036 | 1041 | 1568 | 528 | cytochrome B | OXL25401.1 |
| NEXU01000036 | 1971 | 1681 | 291 | cell division topological specificity factor MinE | OXL25402.1 |
| NEXU01000036 | 2786 | 1974 | 813 | septum site-determining protein MinD | OXL25403.1 |
| NEXU01000036 | 4061 | 3165 | 897 | septum site-determining protein MinC | OXL25404.1 |
| NEXU01000036 | 5259 | 4141 | 1119 | hypothetical protein | OXL25405.1 |
| NEXU01000036 | 6409 | 5441 | 969 | acyltransferase | OXL25406.1 |
| NEXU01000036 | 6899 | 7552 | 654 | hypothetical protein | OXL25407.1 |
| NEXU01000036 | 7853 | 9307 | 1455 | imelysin | OXL25408.1 |
| NEXU01000036 | 9581 | 11245 | 1665 | thiol oxidoreductase | OXL25409.1 |
| NEXU01000036 | 11352 | 12512 | 1161 | peptidase M75 | OXL25410.1 |
| NEXU01000036 | 12516 | 14132 | 1617 | hypothetical protein | OXL25411.1 |
| NEXU01000036 | 14135 | 14812 | 678 | zinc metalloprotease | OXL25412.1 |
| NEXU01000036 | 14928 | 15533 | 606 | SAM-dependent methyltransferase | OXL25413.1 |
| NEXU01000036 | 16188 | 15511 | 678 | hypothetical protein | OXL25414.1 |
| NEXU01000036 | 17187 | 16279 | 909 | LysR family transcriptional regulator | OXL25415.1 |
| NEXU01000036 | 17469 | 18035 | 567 | peroxiredoxin | OXL25416.1 |
| NEXU01000036 | 18447 | 18199 | 249 | hypothetical protein | OXL25417.1 |
| NEXU01000036 | 18952 | 20529 | 1578 | alkyl hydroperoxide reductase subunit F | OXL25418.1 |
| NEXU01000036 | 22323 | 20803 | 1521 | hypothetical protein | OXL25419.1 |
| NEXU01000036 | 23787 | 22930 | 858 | bile acid:sodium symporter | OXL25420.1 |
| NEXU01000036 | 24359 | 24027 | 333 | TIGR01244 family protein | OXL25421.1 |
| NEXU01000036 | 24817 | 24491 | 327 | hypothetical protein | OXL25422.1 |
| NEXU01000036 | 26727 | 25018 | 1710 | sulfate transporter | OXL25423.1 |
| NEXU01000036 | 27075 | 26785 | 291 | transcriptional regulator | OXL25425.1 |
| NEXU01000036 | 27441 | >27802 | N/D | MBL fold metallo-hydrolase | OXL25424.1 |
| NEXU01000037 | <1 | 274 | N/D | MBL fold metallo-hydrolase | OXL25368.1 |
| NEXU01000037 | 424 | 606 | 183 | hypothetical protein | OXL25369.1 |
| NEXU01000037 | 713 | 1153 | 441 | thiol reductase thioredoxin | OXL25370.1 |
| NEXU01000037 | 1689 | 1288 | 402 | hypothetical protein | OXL25371.1 |
| NEXU01000037 | 2199 | 1840 | 360 | hypothetical protein | OXL25372.1 |
| NEXU01000037 | 2635 | 2309 | 327 | BolA family transcriptional regulator | OXL25373.1 |
| NEXU01000037 | 3739 | 2927 | 813 | peptidase M48 | OXL25374.1 |
| NEXU01000037 | 4250 | 4324 | 75 | tRNA-Gly |  |
| NEXU01000037 | 4528 | 5319 | 792 | 7-alpha-hydroxysteroid dehydrogenase | OXL25375.1 |
| NEXU01000037 | 5907 | 5455 | 453 | hypothetical protein | OXL25376.1 |
| NEXU01000037 | 6835 | 6077 | 759 | nicotinamide mononucleotide transporter | OXL25377.1 |
| NEXU01000037 | 8021 | 6963 | 1059 | trifunctional nicotinamide-nucleotide adenylyltransferase/ribosylnicotinamide kinase/transcriptional regulator NadR | OXL25378.1 |
| NEXU01000038 | 2195 | 246 | 1950 | ligand-gated channel | OXL25251.1 |
| NEXU01000038 | 2607 | 4565 | 1959 | mechanosensitive ion channel protein MscS | OXL25252.1 |
| NEXU01000038 | 5255 | 4578 | 678 | 3-methyladenine DNA glycosylase | OXL25253.1 |
| NEXU01000038 | 5578 | 6912 | 1335 | hypothetical protein | OXL25254.1 |
| NEXU01000038 | 7072 | 7935 | 864 | hypothetical protein | OXL25255.1 |
| NEXU01000038 | 8053 | 8307 | 255 | hypothetical protein | OXL25256.1 |
| NEXU01000038 | 8311 | 8595 | 285 | hypothetical protein | OXL25257.1 |
| NEXU01000038 | 8598 | 9125 | 528 | hypothetical protein | OXL25258.1 |
| NEXU01000038 | 9112 | 9387 | 276 | hypothetical protein | OXL25259.1 |
| NEXU01000038 | 9380 | 9658 | 279 | hypothetical protein | OXL25260.1 |
| NEXU01000038 | 9645 | 10220 | 576 | hypothetical protein | OXL25261.1 |
| NEXU01000038 | 10213 | 10836 | 624 | hypothetical protein | OXL25262.1 |
| NEXU01000038 | 10841 | 11074 | 234 | hypothetical protein | OXL25263.1 |
| NEXU01000038 | 11201 | 11641 | 441 | hypothetical protein | OXL25264.1 |
| NEXU01000038 | 11607 | 12446 | 840 | hypothetical protein | OXL25265.1 |
| NEXU01000038 | 12427 | 12978 | 552 | hypothetical protein | OXL25266.1 |
| NEXU01000038 | 12975 | 14333 | 1359 | hypothetical protein | OXL25267.1 |
| NEXU01000038 | 14372 | 14839 | 468 | hypothetical protein | OXL25268.1 |
| NEXU01000038 | 14924 | 15325 | 402 | hypothetical protein | OXL25269.1 |
| NEXU01000038 | 15387 | 15692 | 306 | HNH endonuclease | OXL25270.1 |
| NEXU01000038 | 15804 | 16223 | 420 | hypothetical protein | OXL25271.1 |
| NEXU01000038 | 16233 | 16469 | 237 | hypothetical protein | OXL25272.1 |
| NEXU01000038 | 16530 | 18248 | 1719 | terminase | OXL25273.1 |
| NEXU01000038 | 18317 | 18529 | 213 | hypothetical protein | OXL25274.1 |
| NEXU01000038 | 18696 | 18950 | 255 | hypothetical protein | OXL25275.1 |
| NEXU01000038 | 19025 | 19672 | 648 | hypothetical protein | OXL25276.1 |
| NEXU01000038 | 19669 | 21060 | 1392 | phage major capsid protein | OXL25277.1 |
| NEXU01000038 | 21372 | 22676 | 1305 | phage portal protein | OXL25278.1 |
| NEXU01000038 | 22657 | 22947 | 291 | phage head-tail adapter protein | OXL25279.1 |
| NEXU01000038 | 23008 | 23223 | 216 | hypothetical protein | OXL25280.1 |
| NEXU01000038 | 23229 | 23579 | 351 | head-tail adaptor protein | OXL25281.1 |
| NEXU01000038 | 23582 | 24085 | 504 | hypothetical protein | OXL25282.1 |
| NEXU01000038 | 24082 | 24444 | 363 | hypothetical protein | OXL25283.1 |
| NEXU01000038 | 24474 | 24959 | 486 | hypothetical protein | OXL25284.1 |
| NEXU01000038 | 25021 | 25374 | 354 | hypothetical protein | OXL25285.1 |
| NEXU01000038 | 25458 | 25661 | 204 | hypothetical protein | OXL25300.1 |
| NEXU01000038 | 25719 | 26075 | 357 | hypothetical protein | OXL25286.1 |
| NEXU01000038 | 26146 | 29289 | 3144 | hypothetical protein | OXL25287.1 |
| NEXU01000038 | 29331 | 29567 | 237 | hypothetical protein | OXL25288.1 |
| NEXU01000038 | 29626 | 29964 | 339 | hypothetical protein | OXL25289.1 |
| NEXU01000038 | 30755 | 30081 | 675 | hypothetical protein | OXL25290.1 |
| NEXU01000038 | 33812 | 33081 | 732 | DNA mismatch repair protein MutS | OXL25291.1 |
| NEXU01000038 | 34841 | 33954 | 888 | indole-3-glycerol-phosphate synthase | OXL25292.1 |
| NEXU01000038 | 36072 | 34936 | 1137 | anthranilate phosphoribosyltransferase | OXL25293.1 |
| NEXU01000038 | 36822 | 36199 | 624 | anthranilate/aminodeoxychorismate synthase component II | OXL25294.1 |
| NEXU01000038 | 37669 | 39078 | 1410 | type I glutamate--ammonia ligase | OXL25295.1 |
| NEXU01000038 | 39299 | 40093 | 795 | DUF4124 domain-containing protein | OXL25296.1 |
| NEXU01000038 | 40956 | 40240 | 717 | hypothetical protein | OXL25297.1 |
| NEXU01000038 | 41609 | 42205 | 597 | hypothetical protein | OXL25298.1 |
| NEXU01000038 | 42264 | 44672 | 2409 | peptidase M48 | OXL25299.1 |
| NEXU01000039 | 104 | 868 | 765 | hypothetical protein | OXL25223.1 |
| NEXU01000039 | 1026 | >1892 | N/D | hypothetical protein | OXL25224.1 |
| NEXU01000040 | 15 | 296 | 282 | hypothetical protein |  |
| NEXU01000040 | 497 | 1618 | 1122 | phosphoserine transaminase | OXL25203.1 |
| NEXU01000040 | 1908 | 2778 | 871 | ABC transporter substrate-binding protein |  |
| NEXU01000041 | 16 | 1308 | 1293 | ABC transporter substrate-binding protein |  |
| NEXU01000041 | 1383 | 2162 | 780 | hydroxyacylglutathione hydrolase | OXL25200.1 |
| NEXU01000041 | 2242 | >2487 | N/D | peptide ABC transporter permease | OXL25201.1 |
| NEXU01000042 | 37 | 730 | 694 | peptide ABC transporter permease |  |
| NEXU01000042 | 730 | 1812 | 1083 | ABC transporter permease | OXL25155.1 |
| NEXU01000042 | 1814 | 3484 | 1671 | microcin ABC transporter ATP-binding protein | OXL25156.1 |
| NEXU01000042 | 3686 | 4996 | 1311 | alcohol dehydrogenase | OXL25157.1 |
| NEXU01000042 | 5174 | 6421 | 1248 | methionine sulfoxide reductase | OXL25158.1 |
| NEXU01000042 | 7161 | 6550 | 612 | GTP cyclohydrolase I FolE | OXL25159.1 |
| NEXU01000042 | 7507 | 8313 | 807 | enoyl-[acyl-carrier-protein] reductase | OXL25160.1 |
| NEXU01000042 | 8433 | 9131 | 699 | DUF1275 family protein | OXL25161.1 |
| NEXU01000042 | 9729 | 11150 | 1422 | anion:sodium symporter | OXL25162.1 |
| NEXU01000042 | 11348 | 11145 | 204 | hypothetical protein | OXL25163.1 |
| NEXU01000042 | 11347 | 12384 | 1038 | alpha/beta hydrolase | OXL25164.1 |
| NEXU01000042 | 12432 | 13028 | 597 | hypothetical protein | OXL25165.1 |
| NEXU01000042 | 13368 | 17531 | 4164 | ATP-dependent RNA helicase HrpA | OXL25171.1 |
| NEXU01000042 | 17757 | 18245 | 489 | glcG protein | OXL25166.1 |
| NEXU01000042 | 19894 | 18482 | 1413 | aspartate ammonia-lyase | OXL25167.1 |
| NEXU01000042 | 20582 | 20247 | 336 | hypothetical protein | OXL25168.1 |
| NEXU01000042 | 20865 | 21467 | 603 | SAM-dependent methyltransferase | OXL25169.1 |
| NEXU01000042 | 22268 | 21480 | 789 | hypothetical protein | OXL25170.1 |
| NEXU01000042 | 22744 | 23307 | 564 | hypothetical protein | OXL25172.1 |
| NEXU01000042 | 23710 | 25065 | 1356 | peroxidase | OXL25173.1 |
| NEXU01000043 | <1 | 270 | N/D | peroxidase | OXL24952.1 |
| NEXU01000043 | 315 | 1463 | 1149 | catalase | OXL24953.1 |
| NEXU01000043 | 2581 | 1667 | 915 | EamA family transporter | OXL24954.1 |
| NEXU01000043 | 2759 | 3442 | 684 | hypothetical protein | OXL24955.1 |
| NEXU01000043 | 4086 | 3490 | 597 | nicotinate-nucleotide adenylyltransferase | OXL24956.1 |
| NEXU01000043 | 4789 | 4037 | 753 | nicotinamide mononucleotide transporter | OXL24957.1 |
| NEXU01000043 | 5076 | 6221 | 1146 | beta-ketoacyl-ACP synthase III | OXL24958.1 |
| NEXU01000043 | 6350 | 6847 | 498 | hypothetical protein | OXL24959.1 |
| NEXU01000043 | 7031 | 8119 | 1089 | peptide chain release factor 1 | OXL24960.1 |
| NEXU01000043 | 8159 | 8632 | 474 | hypothetical protein | OXL24961.1 |
| NEXU01000043 | 8766 | 10310 | 1545 | lysine--tRNA ligase | OXL24962.1 |
| NEXU01000043 | 10436 | 11722 | 1287 | hypothetical protein | OXL24963.1 |
| NEXU01000043 | 11860 | 13860 | 2001 | DNA polymerase III subunit gamma/tau | OXL24964.1 |
| NEXU01000044 | 18 | 896 | 879 | LysR family transcriptional regulator | OXL24945.1 |
| NEXU01000044 | 2202 | 1021 | 1182 | aspartate aminotransferase family protein | OXL24941.1 |
| NEXU01000044 | 2441 | 2244 | 198 | DNA gyrase inhibitor YacG | OXL24942.1 |
| NEXU01000044 | 2691 | 3365 | 675 | SAM-dependent methyltransferase | OXL24943.1 |
| NEXU01000044 | >3867 | 3523 | N/D | ABC transporter | OXL24944.1 |
| NEXU01000045 | 1020 | <1 | N/D | ABC transporter | OXL24909.1 |
| NEXU01000045 | 2872 | 1523 | 1350 | phosphoribosylamine--glycine ligase | OXL24934.1 |
| NEXU01000045 | 3314 | 8158 | 4845 | glutamate dehydrogenase | OXL24910.1 |
| NEXU01000045 | 9524 | 8253 | 1272 | hypothetical protein | OXL24911.1 |
| NEXU01000045 | 13447 | 9539 | 3909 | exonuclease SbcD | OXL24912.1 |
| NEXU01000045 | 15113 | 13536 | 1578 | exonuclease sbcCD subunit D | OXL24913.1 |
| NEXU01000045 | 15387 | 16052 | 666 | hypothetical protein | OXL24914.1 |
| NEXU01000045 | 17861 | 16122 | 1740 | uroporphyrinogen-III C-methyltransferase | OXL24915.1 |
| NEXU01000045 | 18886 | 18248 | 639 | phosphoadenylylsulfate reductase | OXL24916.1 |
| NEXU01000045 | 19565 | 18969 | 597 | hypothetical protein | OXL24917.1 |
| NEXU01000045 | 21243 | 19558 | 1686 | sulfite reductase | OXL24918.1 |
| NEXU01000045 | 21549 | 22802 | 1254 | sulfate adenylyltransferase | OXL24919.1 |
| NEXU01000045 | 23427 | 22888 | 540 | hypothetical protein | OXL24920.1 |
| NEXU01000045 | 23863 | 25008 | 1146 | sulfate ABC transporter substrate-binding protein | OXL24921.1 |
| NEXU01000045 | 25254 | 26348 | 1095 | sulfate ABC transporter substrate-binding protein | OXL24922.1 |
| NEXU01000045 | 26590 | 27474 | 885 | sulfate ABC transporter permease subunit CysT | OXL24923.1 |
| NEXU01000045 | 27476 | 28423 | 948 | sulfate ABC transporter permease subunit CysW | OXL24924.1 |
| NEXU01000045 | 28514 | 29236 | 723 | sulfate ABC transporter ATP-binding protein | OXL24925.1 |
| NEXU01000045 | 29690 | 29358 | 333 | transcriptional regulator | OXL24926.1 |
| NEXU01000045 | 29978 | 30169 | 192 | (2Fe-2S)-binding protein | OXL24927.1 |
| NEXU01000045 | 30603 | 31091 | 489 | bacterioferritin | OXL24928.1 |
| NEXU01000045 | 31155 | 31637 | 483 | bacterioferritin | OXL24929.1 |
| NEXU01000045 | 33183 | 31807 | 1377 | MFS transporter | OXL24935.1 |
| NEXU01000045 | 33515 | 34360 | 846 | Gluthatione S-transferase | OXL24930.1 |
| NEXU01000045 | 34667 | 35887 | 1221 | hypothetical protein | OXL24931.1 |
| NEXU01000045 | 36448 | 38121 | 1674 | DEAD/DEAH box helicase | OXL24932.1 |
| NEXU01000045 | 38418 | 39662 | 1245 | hypothetical protein | OXL24933.1 |
| NEXU01000045 | 39961 | 41418 | 1458 | excinuclease ABC subunit A |  |
| NEXU01000046 | 10 | 1368 | 1359 | hypothetical protein |  |
| NEXU01000046 | 1454 | 2455 | 1002 | hypothetical protein | OXL24793.1 |
| NEXU01000046 | 2487 | 3125 | 639 | arylesterase | OXL24794.1 |
| NEXU01000046 | 4366 | 3284 | 1083 | hypothetical protein | OXL24795.1 |
| NEXU01000046 | 4471 | 5214 | 744 | Mlc titration factor A | OXL24796.1 |
| NEXU01000046 | 5273 | 5725 | 453 | hypothetical protein | OXL24797.1 |
| NEXU01000046 | 6547 | 5882 | 666 | hypothetical protein | OXL24798.1 |
| NEXU01000046 | 6719 | 7198 | 480 | hypothetical protein | OXL24824.1 |
| NEXU01000046 | 8066 | 7641 | 426 | hypothetical protein | OXL24799.1 |
| NEXU01000046 | 8963 | 8490 | 474 | hypothetical protein | OXL24800.1 |
| NEXU01000046 | 10061 | 9168 | 894 | hypothetical protein | OXL24801.1 |
| NEXU01000046 | 10290 | 11336 | 1047 | LysR family transcriptional regulator | OXL24802.1 |
| NEXU01000046 | 11644 | 12930 | 1287 | hypothetical protein | OXL24803.1 |
| NEXU01000046 | 13320 | 14210 | 891 | methylisocitrate lyase | OXL24804.1 |
| NEXU01000046 | 14288 | 15415 | 1128 | 2-methylcitrate synthase | OXL24805.1 |
| NEXU01000046 | 15565 | 16143 | 579 | hypothetical protein | OXL24806.1 |
| NEXU01000046 | 16245 | 18902 | 2658 | Fe/S-dependent 2-methylisocitrate dehydratase AcnD | OXL24807.1 |
| NEXU01000046 | 19308 | 19018 | 291 | addiction module toxin RelE | OXL24808.1 |
| NEXU01000046 | 19547 | 19326 | 222 | hypothetical protein | OXL24809.1 |
| NEXU01000046 | 19642 | 20913 | 1272 | 2-methylaconitate cis-trans isomerase PrpF | OXL24810.1 |
| NEXU01000046 | 21374 | 20910 | 465 | hypothetical protein | OXL24811.1 |
| NEXU01000046 | 21491 | 23344 | 1854 | ABC transporter | OXL24812.1 |
| NEXU01000046 | 23439 | 24029 | 591 | GNAT family N-acetyltransferase | OXL24813.1 |
| NEXU01000046 | 24152 | 25654 | 1503 | 2-methylcitrate dehydratase | OXL24814.1 |
| NEXU01000046 | 26454 | 25702 | 753 | short chain dehydrogenase | OXL24815.1 |
| NEXU01000046 | 28181 | 26694 | 1488 | uracil permease | OXL24816.1 |
| NEXU01000046 | 28694 | 30103 | 1410 | hypothetical protein | OXL24817.1 |
| NEXU01000046 | 30190 | 31884 | 1695 | GTP-binding protein | OXL24818.1 |
| NEXU01000046 | 31965 | 32633 | 669 | hypothetical protein | OXL24819.1 |
| NEXU01000046 | 32736 | 33299 | 564 | hypoxanthine-guanine phosphoribosyltransferase | OXL24820.1 |
| NEXU01000046 | 33906 | 33388 | 519 | mep operon protein MepB | OXL24821.1 |
| NEXU01000046 | 36183 | 33934 | 2250 | exodeoxyribonuclease V subunit alpha | OXL24822.1 |
| NEXU01000046 | >40267 | 36224 | N/D | hypothetical protein | OXL24823.1 |
| NEXU01000047 | 442 | 44 | 399 | hypothetical protein |  |
| NEXU01000047 | 4895 | 519 | 4377 | exodeoxyribonuclease V subunit gamma | OXL24787.1 |
| NEXU01000047 | 5414 | 5947 | 534 | hypothetical protein | OXL24788.1 |
| NEXU01000047 | 5989 | 6828 | 840 | hypothetical protein | OXL24789.1 |
| NEXU01000047 | 6901 | 6976 | 76 | tRNA-Arg |  |
| NEXU01000047 | 7189 | >7455 | N/D | hypothetical protein | OXL24790.1 |
| NEXU01000048 | <1 | 1184 | N/D | hypothetical protein | OXL24647.1 |
| NEXU01000048 | 1349 | 2155 | 807 | hypothetical protein | OXL24648.1 |
| NEXU01000048 | 2212 | 2592 | 381 | hypothetical protein | OXL24649.1 |
| NEXU01000048 | 2589 | 4493 | 1905 | hypothetical protein | OXL24650.1 |
| NEXU01000048 | 5147 | 4710 | 438 | hypothetical protein | OXL24651.1 |
| NEXU01000048 | 8058 | 5398 | 2661 | TIGR02687 family protein | OXL24679.1 |
| NEXU01000048 | 11738 | 8058 | 3681 | SAM-dependent methyltransferase | OXL24652.1 |
| NEXU01000048 | 13522 | 11741 | 1782 | hypothetical protein | OXL24653.1 |
| NEXU01000048 | 17214 | 13543 | 3672 | DNA repair protein | OXL24654.1 |
| NEXU01000048 | 17834 | 17232 | 603 | cytoplasmic protein | OXL24655.1 |
| NEXU01000048 | 18430 | 17831 | 600 | hypothetical protein | OXL24656.1 |
| NEXU01000048 | 19922 | 18819 | 1104 | hypothetical protein | OXL24657.1 |
| NEXU01000048 | 21980 | 19950 | 2031 | hypothetical protein | OXL24658.1 |
| NEXU01000048 | 24285 | 21973 | 2313 | hypothetical protein | OXL24659.1 |
| NEXU01000048 | 24841 | 24272 | 570 | hypothetical protein | OXL24660.1 |
| NEXU01000048 | 25305 | 25087 | 219 | XRE family transcriptional regulator | OXL24661.1 |
| NEXU01000048 | 25556 | 25876 | 321 | hypothetical protein | OXL24662.1 |
| NEXU01000048 | 26451 | 27050 | 600 | hypothetical protein | OXL24663.1 |
| NEXU01000048 | 27114 | 27557 | 444 | hypothetical protein | OXL24664.1 |
| NEXU01000048 | 28052 | 28960 | 909 | radical SAM protein | OXL24665.1 |
| NEXU01000048 | 30191 | 28986 | 1206 | hypothetical protein | OXL24666.1 |
| NEXU01000048 | 30913 | 30449 | 465 | hypothetical protein | OXL24667.1 |
| NEXU01000048 | 31356 | 30940 | 417 | hypothetical protein | OXL24668.1 |
| NEXU01000048 | 32636 | 31437 | 1200 | hypothetical protein | OXL24669.1 |
| NEXU01000048 | 32802 | 32647 | 156 | hypothetical protein | OXL24680.1 |
| NEXU01000048 | 33542 | 32820 | 723 | hypothetical protein | OXL24670.1 |
| NEXU01000048 | 34382 | 33669 | 714 | hypothetical protein | OXL24671.1 |
| NEXU01000048 | 35431 | 34958 | 474 | hypothetical protein | OXL24672.1 |
| NEXU01000048 | 36195 | 35515 | 681 | hypothetical protein | OXL24673.1 |
| NEXU01000048 | 36997 | 36233 | 765 | hypothetical protein | OXL24674.1 |
| NEXU01000048 | 37602 | 37213 | 390 | hypothetical protein | OXL24675.1 |
| NEXU01000048 | 38058 | 38774 | 717 | DNA polymerase III subunit epsilon | OXL24676.1 |
| NEXU01000048 | 39670 | 39056 | 615 | IS3 family transposase |  |
| NEXU01000048 | 39910 | 40206 | 297 | hypothetical protein | OXL24677.1 |
| NEXU01000048 | 46784 | 40401 | 6384 | hypothetical protein | OXL24678.1 |
| NEXU01000048 | 47187 | 47023 | 165 | IS1595 family transposase |  |
| NEXU01000048 | 47805 | >47938 | N/D | IS5/IS1182 family transposase | OXL24681.1 |
| NEXU01000049 | 1186 | 71 | 1116 | hypothetical protein | OXL24595.1 |
| NEXU01000049 | 2422 | 1298 | 1125 | hypothetical protein | OXL24596.1 |
| NEXU01000049 | 2779 | 3882 | 1104 | alkene reductase | OXL24597.1 |
| NEXU01000049 | 4977 | 4012 | 966 | short-chain dehydrogenase | OXL24598.1 |
| NEXU01000049 | 6513 | 5212 | 1302 | aminoacetone oxidase family FAD-binding enzyme | OXL24599.1 |
| NEXU01000049 | 7379 | 7143 | 237 | hypothetical protein | OXL24600.1 |
| NEXU01000049 | 7797 | 9758 | 1962 | acetate--CoA ligase | OXL24601.1 |
| NEXU01000049 | 10140 | 11360 | 1221 | NADP transhydrogenase subunit alpha | OXL24602.1 |
| NEXU01000049 | 11521 | 12855 | 1335 | hypothetical protein | OXL24603.1 |
| NEXU01000049 | 12969 | 14489 | 1521 | hypothetical protein | OXL24604.1 |
| NEXU01000049 | 14764 | 16239 | 1476 | hypothetical protein | OXL24605.1 |
| NEXU01000049 | 16422 | 17459 | 1038 | hypothetical protein | OXL24606.1 |
| NEXU01000049 | 17692 | 17952 | 261 | hypothetical protein | OXL24607.1 |
| NEXU01000049 | 17955 | 18479 | 525 | hypothetical protein | OXL24608.1 |
| NEXU01000049 | 18476 | 19066 | 591 | hypothetical protein | OXL24609.1 |
| NEXU01000049 | 19059 | 19652 | 594 | hypothetical protein | OXL24610.1 |
| NEXU01000049 | 19759 | 21882 | 2124 | hypothetical protein | OXL24611.1 |
| NEXU01000049 | 22197 | 22415 | 219 | hypothetical protein | OXL24612.1 |
| NEXU01000049 | 22616 | 23092 | 477 | hypothetical protein | OXL24613.1 |
| NEXU01000049 | 23079 | 23372 | 294 | hypothetical protein | OXL24614.1 |
| NEXU01000049 | 24277 | 24480 | 204 | hypothetical protein | OXL24615.1 |
| NEXU01000049 | 25930 | 25142 | 789 | hypothetical protein | OXL24616.1 |
| NEXU01000049 | 26366 | 25932 | 435 | hypothetical protein | OXL24617.1 |
| NEXU01000049 | 26848 | 26447 | 402 | hypothetical protein | OXL24642.1 |
| NEXU01000049 | 27684 | 26833 | 852 | hypothetical protein | OXL24618.1 |
| NEXU01000049 | 28496 | 29167 | 672 | CDP-diacylglycerol--glycerol-3-phosphate 3-phosphatidyltransferase | OXL24619.1 |
| NEXU01000049 | 29439 | 30263 | 825 | hypothetical protein | OXL24620.1 |
| NEXU01000049 | 30921 | 30427 | 495 | general stress protein | OXL24621.1 |
| NEXU01000049 | 31785 | 31291 | 495 | general stress protein | OXL24622.1 |
| NEXU01000049 | 32248 | 36261 | 4014 | phosphoribosylformylglycinamidine synthase | OXL24623.1 |
| NEXU01000049 | 36520 | 37638 | 1119 | hypothetical protein | OXL24624.1 |
| NEXU01000049 | 37635 | 39389 | 1755 | hypothetical protein | OXL24625.1 |
| NEXU01000049 | 40779 | 39754 | 1026 | L-threonine 3-dehydrogenase | OXL24626.1 |
| NEXU01000049 | 42052 | 40862 | 1191 | glycine C-acetyltransferase | OXL24627.1 |
| NEXU01000049 | 42841 | 42644 | 198 | hypothetical protein | OXL24628.1 |
| NEXU01000049 | 43611 | 42865 | 747 | GMP synthase | OXL24629.1 |
| NEXU01000049 | 44576 | 43698 | 879 | N-acetylmuramoyl-L-alanine amidase | OXL24630.1 |
| NEXU01000049 | 45448 | 44612 | 837 | hypothetical protein | OXL24631.1 |
| NEXU01000049 | 45748 | 45473 | 276 | hypothetical protein | OXL24632.1 |
| NEXU01000049 | 46536 | 45745 | 792 | MOSC domain-containing protein | OXL24633.1 |
| NEXU01000049 | 47208 | 46795 | 414 | ribosome silencing factor | OXL24643.1 |
| NEXU01000049 | 48276 | 47350 | 927 | nicotinate-nicotinamide nucleotide adenylyltransferase | OXL24634.1 |
| NEXU01000049 | 50788 | 48632 | 2157 | phosphate acetyltransferase | OXL24635.1 |
| NEXU01000049 | 52115 | 50883 | 1233 | acetate kinase | OXL24636.1 |
| NEXU01000049 | 52819 | 53145 | 327 | hypothetical protein | OXL24637.1 |
| NEXU01000049 | 53241 | 54593 | 1353 | diaminopimelate decarboxylase | OXL24638.1 |
| NEXU01000049 | 55030 | 55917 | 888 | diaminopimelate epimerase | OXL24639.1 |
| NEXU01000049 | 55976 | 57013 | 1038 | recombinase XerC | OXL24644.1 |
| NEXU01000049 | 57990 | 57091 | 900 | carbon-nitrogen hydrolase | OXL24640.1 |
| NEXU01000049 | 58316 | 60001 | 1686 | RNA polymerase sigma-54 factor | OXL24645.1 |
| NEXU01000049 | 60393 | 60773 | 381 | ribosomal subunit interface protein | OXL24641.1 |
| NEXU01000050 | 301 | 101 | 201 | thiamine biosynthesis protein ThiS | OXL24257.1 |
| NEXU01000050 | 775 | 401 | 375 | hypothetical protein | OXL24258.1 |
| NEXU01000050 | 1852 | 827 | 1026 | RNA polymerase factor sigma-32 | OXL24259.1 |
| NEXU01000050 | 2407 | 1931 | 477 | hypothetical protein | OXL24260.1 |
| NEXU01000050 | 2818 | 4479 | 1662 | peptidase M48, Ste24p | OXL24261.1 |
| NEXU01000050 | 5049 | 4600 | 450 | glutamyl-tRNA amidotransferase | OXL24262.1 |
| NEXU01000050 | 5490 | 5275 | 216 | 30S ribosomal protein S21 | OXL24263.1 |
| NEXU01000050 | 5931 | 6332 | 402 | hypothetical protein | OXL24277.1 |
| NEXU01000050 | 6450 | 6974 | 525 | cytochrome C | OXL24264.1 |
| NEXU01000050 | 7137 | 8186 | 1050 | tRNA (adenosine(37)-N6)-threonylcarbamoyltransferase complex transferase subunit TsaD | OXL24265.1 |
| NEXU01000050 | 8251 | 8622 | 372 | chromosome condensation protein CrcB | OXL24266.1 |
| NEXU01000050 | 9540 | 8650 | 891 | LysR family transcriptional regulator | OXL24267.1 |
| NEXU01000050 | 10499 | 9657 | 843 | spermidine/putrescine ABC transporter permease PotC | OXL24268.1 |
| NEXU01000050 | 11377 | 10499 | 879 | spermidine/putrescine ABC transporter permease PotB | OXL24269.1 |
| NEXU01000050 | 12651 | 11377 | 1275 | putrescine/spermidine ABC transporter ATP-binding protein | OXL24270.1 |
| NEXU01000050 | 13127 | 13765 | 639 | carbonic anhydrase | OXL24271.1 |
| NEXU01000050 | 13879 | 14448 | 570 | lactoylglutathione lyase | OXL24272.1 |
| NEXU01000050 | 15019 | 14546 | 474 | cytochrome C | OXL24273.1 |
| NEXU01000050 | 16511 | 15501 | 1011 | coproporphyrinogen III oxidase | OXL24274.1 |
| NEXU01000050 | 17222 | 16530 | 693 | GTP cyclohydrolase II | OXL24275.1 |
| NEXU01000050 | 17712 | >18104 | N/D | 1-deoxy-D-xylulose-5-phosphate synthase | OXL24276.1 |
| NEXU01000051 | <1 | 1565 | N/D | 1-deoxy-D-xylulose-5-phosphate synthase | OXL24210.1 |
| NEXU01000051 | 1979 | 2806 | 828 | inositol monophosphatase | OXL24211.1 |
| NEXU01000051 | 4282 | 2855 | 1428 | wax ester/triacylglycerol synthase family O-acyltransferase | OXL24212.1 |
| NEXU01000051 | 5419 | 4595 | 825 | thiazole synthase | OXL24213.1 |
| NEXU01000051 | 5728 | 7296 | 1569 | type II secretion system protein GspE | OXL24214.1 |
| NEXU01000051 | 7826 | 7332 | 495 | reactive intermediate/imine deaminase | OXL24215.1 |
| NEXU01000051 | 8694 | 7954 | 741 | DUF4442 domain-containing protein | OXL24216.1 |
| NEXU01000051 | 8997 | 10484 | 1488 | endonuclease | OXL24217.1 |
| NEXU01000051 | 10512 | 12029 | 1518 | hypothetical protein | OXL24224.1 |
| NEXU01000051 | 12276 | 12352 | 77 | tRNA-Pro |  |
| NEXU01000051 | 12448 | 12524 | 77 | tRNA-Arg |  |
| NEXU01000051 | 12565 | 12640 | 76 | tRNA-His |  |
| NEXU01000051 | 13070 | 14005 | 936 | AraC family transcriptional regulator | OXL24218.1 |
| NEXU01000051 | 14314 | 15423 | 1110 | hypothetical protein | OXL24219.1 |
| NEXU01000051 | 15505 | 16707 | 1203 | type II secretion system protein GspF | OXL24220.1 |
| NEXU01000051 | 17189 | 17704 | 516 | type II secretion system protein GspG | OXL24221.1 |
| NEXU01000051 | 18058 | 18627 | 570 | thiol:disulfide interchange protein | OXL24222.1 |
| NEXU01000051 | 18676 | 20877 | 2202 | cytochrome C biogenesis protein | OXL24223.1 |
| NEXU01000052 | 47 | 1441 | 1395 | phospholipase | OXL24101.1 |
| NEXU01000052 | 1469 | 2317 | 849 | competence protein F | OXL24028.1 |
| NEXU01000052 | 2663 | 3118 | 456 | pilus assembly protein | OXL24029.1 |
| NEXU01000052 | 3109 | 3705 | 597 | type IV pilus modification protein PilV | OXL24030.1 |
| NEXU01000052 | 3707 | 4819 | 1113 | hypothetical protein | OXL24031.1 |
| NEXU01000052 | 4819 | 5631 | 813 | hypothetical protein | OXL24032.1 |
| NEXU01000052 | 5678 | 9817 | 4140 | hypothetical protein | OXL24033.1 |
| NEXU01000052 | 9819 | 10325 | 507 | hypothetical protein | OXL24034.1 |
| NEXU01000052 | 10412 | 10861 | 450 | hypothetical protein | OXL24035.1 |
| NEXU01000052 | 11162 | 11515 | 354 | hypothetical protein | OXL24036.1 |
| NEXU01000052 | 11603 | 13456 | 1854 | hypothetical protein | OXL24037.1 |
| NEXU01000052 | 13594 | 13953 | 360 | hypothetical protein | OXL24038.1 |
| NEXU01000052 | 14390 | 14067 | 324 | ferredoxin | OXL24039.1 |
| NEXU01000052 | 17581 | 14483 | 3099 | DNA mismatch repair protein MutS | OXL24040.1 |
| NEXU01000052 | 17939 | 20728 | 2790 | preprotein translocase subunit SecA | OXL24041.1 |
| NEXU01000052 | 20904 | 21440 | 537 | hypothetical protein | OXL24042.1 |
| NEXU01000052 | 22250 | 21588 | 663 | hypothetical protein | OXL24043.1 |
| NEXU01000052 | 23375 | 22527 | 849 | photosystem reaction center subunit H | OXL24044.1 |
| NEXU01000052 | 24979 | 23798 | 1182 | acetyl-CoA acetyltransferase | OXL24045.1 |
| NEXU01000052 | 26450 | 25107 | 1344 | homoserine dehydrogenase | OXL24046.1 |
| NEXU01000052 | 27537 | 26683 | 855 | thiol:disulfide interchange protein | OXL24047.1 |
| NEXU01000052 | 27987 | 28916 | 930 | hypothetical protein | OXL24048.1 |
| NEXU01000052 | 33383 | 29019 | 4365 | ribonuclease | OXL24049.1 |
| NEXU01000052 | 34613 | 35629 | 1017 | pseudouridine synthase | OXL24050.1 |
| NEXU01000052 | 35762 | 36472 | 711 | phosphoglycolate phosphatase | OXL24051.1 |
| NEXU01000052 | 37335 | 36562 | 774 | YihA family ribosome biogenesis GTP-binding protein | OXL24052.1 |
| NEXU01000052 | 37722 | 38057 | 336 | cytochrome C | OXL24053.1 |
| NEXU01000052 | 38382 | 39050 | 669 | cytochrome C | OXL24054.1 |
| NEXU01000052 | 39302 | 40444 | 1143 | hypothetical protein | OXL24055.1 |
| NEXU01000052 | 40432 | 40830 | 399 | RnfH family protein | OXL24056.1 |
| NEXU01000052 | 41272 | 40856 | 417 | hypothetical protein | OXL24102.1 |
| NEXU01000052 | 41598 | 42032 | 435 | ferric iron uptake transcriptional regulator | OXL24057.1 |
| NEXU01000052 | 43331 | 42210 | 1122 | type IV pili twitching motility protein PilT | OXL24058.1 |
| NEXU01000052 | 44717 | 43662 | 1056 | twitching motility protein PilT | OXL24059.1 |
| NEXU01000052 | 45072 | 45836 | 765 | YggS family pyridoxal phosphate enzyme | OXL24060.1 |
| NEXU01000052 | 47280 | 46003 | 1278 | hypothetical protein | OXL24061.1 |
| NEXU01000052 | 49226 | 47523 | 1704 | NAD+ synthase | OXL24062.1 |
| NEXU01000052 | 49995 | 51137 | 1143 | AI-2E family transporter | OXL24063.1 |
| NEXU01000052 | 51293 | 52408 | 1116 | erythronate-4-phosphate dehydrogenase | OXL24103.1 |
| NEXU01000052 | 52469 | 53497 | 1029 | EF-P lysine aminoacylase GenX | OXL24064.1 |
| NEXU01000052 | 54227 | 53589 | 639 | epimerase | OXL24065.1 |
| NEXU01000052 | 54877 | 54428 | 450 | DUF188 domain-containing protein | OXL24066.1 |
| NEXU01000052 | 56211 | 54877 | 1335 | 5-(carboxyamino)imidazole ribonucleotide synthase | OXL24067.1 |
| NEXU01000052 | 56982 | 56422 | 561 | 5-(carboxyamino)imidazole ribonucleotide mutase | OXL24068.1 |
| NEXU01000052 | 58047 | 57103 | 945 | phosphoesterase | OXL24069.1 |
| NEXU01000052 | 58776 | 58168 | 609 | rhomboid family intramembrane serine protease | OXL24070.1 |
| NEXU01000052 | 59256 | 60806 | 1551 | hypothetical protein | OXL24071.1 |
| NEXU01000052 | 61292 | 62968 | 1677 | cell envelope biogenesis protein OmpA | OXL24072.1 |
| NEXU01000052 | 64194 | 63103 | 1092 | beta-N-acetylhexosaminidase | OXL24073.1 |
| NEXU01000052 | 64668 | 66878 | 2211 | tail-specific protease | OXL24074.1 |
| NEXU01000052 | 67860 | 67375 | 486 | peptidylprolyl isomerase | OXL24075.1 |
| NEXU01000052 | 68189 | 70084 | 1896 | peptidase M61 | OXL24076.1 |
| NEXU01000052 | 70599 | 70234 | 366 | Gluthatione S-transferase | OXL24077.1 |
| NEXU01000052 | 71559 | 70768 | 792 | DUF305 domain-containing protein | OXL24078.1 |
| NEXU01000052 | 72389 | 71763 | 627 | pseudouridine synthase | OXL24079.1 |
| NEXU01000052 | 72606 | 73865 | 1260 | NADP-dependent isocitrate dehydrogenase | OXL24080.1 |
| NEXU01000052 | 76463 | 74244 | 2220 | isocitrate dehydrogenase (NADP(+)) | OXL24081.1 |
| NEXU01000052 | 77114 | 78469 | 1356 | GNAT family N-acetyltransferase | OXL24082.1 |
| NEXU01000052 | 78715 | 79221 | 507 | chorismate--pyruvate lyase | OXL24083.1 |
| NEXU01000052 | 79624 | 80880 | 1257 | serine hydroxymethyltransferase | OXL24084.1 |
| NEXU01000052 | 81035 | 81814 | 780 | hypothetical protein | OXL24085.1 |
| NEXU01000052 | 81874 | 82227 | 354 | transposase | OXL24086.1 |
| NEXU01000052 | 82268 | 82720 | 453 | transposase | OXL24104.1 |
| NEXU01000052 | 82696 | 83085 | 390 | hypothetical protein | OXL24087.1 |
| NEXU01000052 | 84436 | 83165 | 1272 | sodium/glutamate symporter | OXL24088.1 |
| NEXU01000052 | 84877 | 84539 | 339 | hypothetical protein | OXL24089.1 |
| NEXU01000052 | 85649 | 84879 | 771 | DNA-binding protein | OXL24090.1 |
| NEXU01000052 | 87112 | 85811 | 1302 | glutamate-5-semialdehyde dehydrogenase | OXL24091.1 |
| NEXU01000052 | 87633 | 87971 | 339 | hypothetical protein | OXL24092.1 |
| NEXU01000052 | 88169 | 88966 | 798 | histidine/lysine/arginine/ornithine ABC transporter ATP-binding protein | OXL24093.1 |
| NEXU01000052 | 89304 | 90101 | 798 | ABC transporter substrate-binding protein | OXL24094.1 |
| NEXU01000052 | 90243 | 91178 | 936 | ABC transporter substrate-binding protein | OXL24095.1 |
| NEXU01000052 | 91269 | 92255 | 987 | ABC transporter substrate-binding protein | OXL24096.1 |
| NEXU01000052 | 92671 | 93405 | 735 | ABC transporter permease | OXL24097.1 |
| NEXU01000052 | 93420 | 94145 | 726 | ABC transporter permease | OXL24098.1 |
| NEXU01000052 | 94227 | 94943 | 717 | isomerase/hydrolase | OXL24099.1 |
| NEXU01000052 | 95208 | 97421 | 2214 | phosphatase | OXL24100.1 |
| NEXU01000052 | 97651 | >98787 | N/D | hypothetical protein | OXL24105.1 |
| NEXU01000053 | 1757 | 129 | 1629 | sodium transporter | OXL23931.1 |
| NEXU01000053 | 2946 | 2275 | 672 | hypothetical protein | OXL23932.1 |
| NEXU01000054 | 1957 | 638 | 1320 | short-chain fatty acid transporter | OXL23892.1 |
| NEXU01000054 | 2250 | 4184 | 1935 | autotransporter domain-containing protein | OXL23893.1 |
| NEXU01000054 | 4986 | 4348 | 639 | hypothetical protein | OXL23894.1 |
| NEXU01000054 | 5855 | 5271 | 585 | hypothetical protein | OXL23895.1 |
| NEXU01000054 | 6786 | 5881 | 906 | hypothetical protein | OXL23896.1 |
| NEXU01000054 | 9582 | 7537 | 2046 | Choline-glycine betaine transporter, BCCT family | OXL23897.1 |
| NEXU01000054 | 11578 | 9878 | 1701 | choline dehydrogenase | OXL23913.1 |
| NEXU01000054 | 13199 | 11697 | 1503 | betaine-aldehyde dehydrogenase | OXL23898.1 |
| NEXU01000054 | 13973 | 13404 | 570 | TetR family transcriptional regulator | OXL23899.1 |
| NEXU01000054 | 14338 | 13994 | 345 | QacE family quaternary ammonium compound efflux SMR transporter | OXL23900.1 |
| NEXU01000054 | 15345 | 14668 | 678 | anti-sigma factor | OXL23901.1 |
| NEXU01000054 | 15724 | 16923 | 1200 | sugar transporter | OXL23902.1 |
| NEXU01000054 | 17458 | 17045 | 414 | aminoacyl-tRNA hydrolase | OXL23903.1 |
| NEXU01000054 | 18365 | 17520 | 846 | CoA ester lyase | OXL23904.1 |
| NEXU01000054 | 19241 | 18480 | 762 | short-chain dehydrogenase | OXL23905.1 |
| NEXU01000054 | 20200 | 19418 | 783 | oxidoreductase | OXL23906.1 |
| NEXU01000054 | 21211 | 20204 | 1008 | NADP-dependent oxidoreductase | OXL23907.1 |
| NEXU01000054 | 23085 | 21268 | 1818 | acyl-CoA dehydrogenase | OXL23908.1 |
| NEXU01000054 | 23295 | 24341 | 1047 | AraC family transcriptional regulator | OXL23909.1 |
| NEXU01000054 | 25684 | 24371 | 1314 | LD-carboxypeptidase | OXL23914.1 |
| NEXU01000054 | 25979 | 26389 | 411 | hypothetical protein | OXL23910.1 |
| NEXU01000054 | 26782 | 26477 | 306 | hypothetical protein | OXL23911.1 |
| NEXU01000054 | >27230 | 26921 | N/D | hypothetical protein | OXL23912.1 |
| NEXU01000055 | 643 | >1215 | N/D | ribonuclease HI | OXL23859.1 |
| NEXU01000056 | 36 | 1197 | 1162 | DNA polymerase III subunit epsilon |  |
| NEXU01000056 | 1346 | 3028 | 1683 | hypothetical protein | OXL23823.1 |
| NEXU01000056 | 3097 | 4134 | 1038 | NADH pyrophosphatase | OXL23824.1 |
| NEXU01000056 | 4227 | 5138 | 912 | iron-sulfur protein | OXL23825.1 |
| NEXU01000056 | 5231 | 5926 | 696 | endonuclease III | OXL23826.1 |
| NEXU01000056 | 5975 | 6562 | 588 | dimethyladenosine transferase | OXL23827.1 |
| NEXU01000056 | 6707 | 7873 | 1167 | methionine adenosyltransferase | OXL23828.1 |
| NEXU01000056 | 8069 | 8365 | 297 | hypothetical protein | OXL23833.1 |
| NEXU01000056 | 8607 | 8972 | 366 | hypothetical protein | OXL23829.1 |
| NEXU01000056 | 10740 | 9085 | 1656 | Choline-glycine betaine transporter, BCCT family | OXL23830.1 |
| NEXU01000056 | 10901 | 11137 | 237 | hypothetical protein | OXL23831.1 |
| NEXU01000056 | 12379 | 11126 | 1254 | glucose dehydrogenase | OXL23832.1 |
| NEXU01000056 | 13383 | 12552 | 832 | ATP-dependent chaperone ClpB |  |
| NEXU01000057 | 2095 | 1 | 2095 | ATP-dependent chaperone ClpB |  |
| NEXU01000057 | 2948 | 2367 | 582 | hypothetical protein | OXL23738.1 |
| NEXU01000057 | 3860 | 3081 | 780 | 3-hydroxyacyl-CoA dehydrogenase | OXL23739.1 |
| NEXU01000057 | 5618 | 3924 | 1695 | AMP-binding protein | OXL23740.1 |
| NEXU01000057 | 6949 | 5816 | 1134 | DNA topoisomerase I | OXL23741.1 |
| NEXU01000057 | 7122 | 7598 | 477 | GNAT family N-acetyltransferase | OXL23742.1 |
| NEXU01000057 | 8004 | 7720 | 285 | hypothetical protein | OXL23743.1 |
| NEXU01000057 | 9126 | 8047 | 1080 | peptide chain release factor 2 | OXL23744.1 |
| NEXU01000057 | 9951 | 9244 | 708 | hydrolase | OXL23745.1 |
| NEXU01000057 | 10532 | 9996 | 537 | gamma carbonic anhydrase family protein | OXL23746.1 |
| NEXU01000057 | 10708 | 11277 | 570 | phosphatase PAP2 family protein | OXL23747.1 |
| NEXU01000057 | 11641 | 12882 | 1242 | carbamoyl-phosphate synthase small subunit | OXL23748.1 |
| NEXU01000057 | 13040 | 16294 | 3255 | carbamoyl phosphate synthase large subunit | OXL23749.1 |
| NEXU01000057 | 16644 | 17120 | 477 | transcription elongation factor GreA | OXL23750.1 |
| NEXU01000057 | 17476 | 18408 | 933 | hypothetical protein | OXL23751.1 |
| NEXU01000057 | 18708 | 19211 | 504 | hypothetical protein | OXL23752.1 |
| NEXU01000057 | 19349 | 20617 | 1269 | putative DNA modification/repair radical SAM protein | OXL23753.1 |
| NEXU01000057 | 20674 | 21558 | 885 | hypothetical protein | OXL23754.1 |
| NEXU01000057 | 21721 | 22896 | 1176 | glycosyl transferase family 1 |  |
| NEXU01000057 | 24773 | 23271 | 1503 | succinate-semialdehyde dehydrogenase (NADP(+)) | OXL23781.1 |
| NEXU01000057 | 26390 | 25188 | 1203 | acyl-CoA dehydrogenase | OXL23755.1 |
| NEXU01000057 | 26907 | 26515 | 393 | hypothetical protein | OXL23756.1 |
| NEXU01000057 | 27095 | 27697 | 603 | hypothetical protein | OXL23757.1 |
| NEXU01000057 | 29475 | 27748 | 1728 | transcriptional regulator | OXL23758.1 |
| NEXU01000057 | 30267 | 29587 | 681 | hypothetical protein | OXL23759.1 |
| NEXU01000057 | 30560 | 31792 | 1233 | RNA-splicing ligase RtcB | OXL23760.1 |
| NEXU01000057 | 31859 | 32560 | 702 | hypothetical protein | OXL23761.1 |
| NEXU01000057 | 32607 | 33065 | 459 | septicolysin | OXL23762.1 |
| NEXU01000057 | 33664 | 34767 | 1104 | RNA 3'-phosphate cyclase | OXL23763.1 |
| NEXU01000057 | 34983 | 36785 | 1803 | FMN-binding glutamate synthase family protein | OXL23782.1 |
| NEXU01000057 | 36845 | 37666 | 822 | hypothetical protein | OXL23764.1 |
| NEXU01000057 | 38967 | 37744 | 1224 | hypothetical protein | OXL23765.1 |
| NEXU01000057 | 39956 | 39093 | 864 | ATP-binding protein | OXL23766.1 |
| NEXU01000057 | 40862 | 40257 | 606 | hypothetical protein | OXL23767.1 |
| NEXU01000057 | 41420 | 41100 | 321 | hypothetical protein | OXL23768.1 |
| NEXU01000057 | 42161 | 43363 | 1203 | glycine cleavage system protein T | OXL23769.1 |
| NEXU01000057 | 43433 | 43813 | 381 | glycine cleavage system protein H | OXL23770.1 |
| NEXU01000057 | 44088 | 44900 | 813 | nitroreductase family protein | OXL23771.1 |
| NEXU01000057 | 44923 | 45294 | 372 | hypothetical protein | OXL23772.1 |
| NEXU01000057 | 45393 | 48293 | 2901 | glycine dehydrogenase (aminomethyl-transferring) | OXL23773.1 |
| NEXU01000057 | 48505 | 49179 | 675 | alpha/beta hydrolase | OXL23774.1 |
| NEXU01000057 | 50506 | 49196 | 1311 | ribonuclease D | OXL23775.1 |
| NEXU01000057 | 51293 | 50700 | 594 | recombination protein RecR | OXL23776.1 |
| NEXU01000057 | 51795 | 51466 | 330 | YbaB/EbfC family nucleoid-associated protein | OXL23777.1 |
| NEXU01000057 | 53154 | 51880 | 1275 | O-succinylhomoserine sulfhydrylase | OXL23778.1 |
| NEXU01000057 | 53552 | 55225 | 1674 | phospholipase | OXL23779.1 |
| NEXU01000057 | 55485 | >55855 | N/D | MFS transporter | OXL23780.1 |
| NEXU01000058 | 5 | 1141 | 1137 | MFS transporter | OXL23716.1 |
| NEXU01000058 | 1371 | 2342 | 972 | tRNA dihydrouridine(20/20a) synthase DusA | OXL23711.1 |
| NEXU01000058 | 3197 | 2400 | 798 | CPBP family intramembrane metalloprotease | OXL23712.1 |
| NEXU01000058 | 4096 | 3209 | 888 | alpha/beta hydrolase | OXL23713.1 |
| NEXU01000058 | 5287 | 4232 | 1056 | signal peptide peptidase SppA | OXL23714.1 |
| NEXU01000058 | 8526 | 5503 | 3024 | competence protein ComEC | OXL23717.1 |
| NEXU01000058 | 8913 | 9443 | 531 | hypothetical protein | OXL23718.1 |
| NEXU01000058 | 9800 | 9489 | 312 | hypothetical protein | OXL23715.1 |
| NEXU01000059 | 285 | 64 | 222 | hypothetical protein |  |
| NEXU01000059 | 1140 | 460 | 681 | lipoprotein-releasing system ATP-binding protein LolD | OXL23662.1 |
| NEXU01000059 | 2420 | 1185 | 1236 | lipoprotein-releasing system transmembrane subunit LolC | OXL23663.1 |
| NEXU01000059 | 2870 | 3853 | 984 | hypothetical protein | OXL23664.1 |
| NEXU01000059 | 4373 | 3984 | 390 | SCP-2 sterol transfer family protein | OXL23665.1 |
| NEXU01000059 | 5225 | 6706 | 1482 | cytochrome-c oxidase, cbb3-type subunit I | OXL23666.1 |
| NEXU01000059 | 6716 | 7354 | 639 | cytochrome-c oxidase, cbb3-type subunit II | OXL23667.1 |
| NEXU01000059 | 7354 | 7536 | 183 | CcoQ/FixQ family Cbb3-type cytochrome c oxidase assembly chaperone | OXL23668.1 |
| NEXU01000059 | 7533 | 8681 | 1149 | cytochrome-c oxidase, cbb3-type subunit III | OXL23669.1 |
| NEXU01000059 | 9152 | 10561 | 1410 | cytochrome c oxidase accessory protein CcoG | OXL23670.1 |
| NEXU01000059 | 10774 | 11355 | 582 | hypothetical protein | OXL23671.1 |
| NEXU01000059 | 12638 | 11460 | 1179 | tRNA 2-thiocytidine(32) synthetase TtcA | OXL23672.1 |
| NEXU01000059 | 13041 | 13820 | 780 | 7-cyano-7-deazaguanine synthase QueC | OXL23673.1 |
| NEXU01000059 | 14088 | 14417 | 330 | hypothetical protein | OXL23674.1 |
| NEXU01000059 | 14579 | 15076 | 498 | peroxiredoxin | OXL23675.1 |
| NEXU01000059 | 15238 | 16740 | 1503 | dihydrolipoyl dehydrogenase | OXL23676.1 |
| NEXU01000059 | 16903 | 18099 | 1197 | mechanosensitive ion channel protein MscS | OXL23677.1 |
| NEXU01000059 | 18314 | 20647 | 2334 | 23S rRNA (guanine(2445)-N(2))/(guanine(2069)-N(7))-methyltransferase | OXL23678.1 |
| NEXU01000059 | 20877 | 21452 | 576 | chemical-damaging agent resistance protein C | OXL23679.1 |
| NEXU01000059 | 21715 | 22779 | 1065 | hypothetical protein | OXL23680.1 |
| NEXU01000059 | 23428 | 24003 | 576 | chemical-damaging agent resistance protein C | OXL23681.1 |
| NEXU01000059 | 24189 | 24941 | 753 | TIGR00266 family protein | OXL23682.1 |
| NEXU01000059 | 24976 | 25584 | 609 | Tellurium resistance protein terZ | OXL23683.1 |
| NEXU01000059 | 26955 | 25708 | 1248 | tellurium resistance protein TerA | OXL23684.1 |
| NEXU01000059 | 27194 | 28423 | 1230 | carboxylate--amine ligase | OXL23685.1 |
| NEXU01000059 | 28698 | 29948 | 1251 | serine protease | OXL23686.1 |
| NEXU01000059 | 30042 | 31325 | 1284 | hypothetical protein | OXL23687.1 |
| NEXU01000059 | 31374 | 32183 | 810 | hypothetical protein | OXL23688.1 |
| NEXU01000059 | 32274 | 33455 | 1182 | hypothetical protein | OXL23689.1 |
| NEXU01000059 | 33540 | 34604 | 1065 | citrate lyase subunit beta-like protein | OXL23690.1 |
| NEXU01000059 | 35535 | 34723 | 813 | alpha/beta hydrolase | OXL23691.1 |
| NEXU01000059 | 35895 | 36692 | 798 | enoyl-CoA hydratase | OXL23692.1 |
| NEXU01000059 | 37258 | 36815 | 444 | hypothetical protein | OXL23693.1 |
| NEXU01000059 | 40068 | 37465 | 2604 | bifunctional aconitate hydratase 2/2-methylisocitrate dehydratase | OXL23694.1 |
| NEXU01000059 | 40560 | 41009 | 450 | 4-carboxymuconolactone decarboxylase | OXL23695.1 |
| NEXU01000059 | 41172 | 42218 | 1047 | lipoyl synthase | OXL23696.1 |
| NEXU01000059 | 42368 | 42754 | 387 | hypothetical protein | OXL23697.1 |
| NEXU01000059 | 43252 | 42884 | 369 | hypothetical protein | OXL23698.1 |
| NEXU01000059 | 44360 | 43572 | 789 | hypothetical protein | OXL23699.1 |
| NEXU01000059 | 46548 | 44437 | 2112 | excinuclease ABC subunit B | OXL23700.1 |
| NEXU01000059 | 46733 | 47152 | 420 | aldehyde-activating protein | OXL23701.1 |
| NEXU01000059 | 47494 | 47991 | 498 | Dps-like DNA binding protein | OXL23702.1 |
| NEXU01000059 | 48858 | 48145 | 714 | hypothetical protein | OXL23703.1 |
| NEXU01000059 | 50022 | 48925 | 1098 | cation transporter | OXL23704.1 |
| NEXU01000059 | 50727 | 50179 | 549 | acyl-CoA thioesterase | OXL23705.1 |
| NEXU01000059 | 51234 | 50824 | 411 | hypothetical protein | OXL23706.1 |
| NEXU01000059 | 52707 | 51310 | 1398 | SAM-dependent methyltransferase | OXL23707.1 |
| NEXU01000059 | 53913 | 52813 | 1101 | DUF1365 domain-containing protein | OXL23708.1 |
| NEXU01000059 | >55474 | 53937 | N/D | FAD-dependent oxidoreductase | OXL23709.1 |
| NEXU01000060 | 550 | 8 | 543 | FAD-dependent oxidoreductase |  |
| NEXU01000060 | 1356 | 616 | 741 | short-chain dehydrogenase | OXL23567.1 |
| NEXU01000060 | 1760 | 1835 | 76 | tRNA-Asn |  |
| NEXU01000060 | 2803 | 1991 | 813 | hypothetical protein | OXL23568.1 |
| NEXU01000060 | 4334 | 2988 | 1347 | type VI secretion system-associated protein | OXL23569.1 |
| NEXU01000060 | 5066 | 4344 | 723 | hypothetical protein | OXL23570.1 |
| NEXU01000060 | 5895 | 5107 | 789 | hypothetical protein | OXL23579.1 |
| NEXU01000060 | 6606 | 8153 | 1548 | hypothetical protein | OXL23571.1 |
| NEXU01000060 | 8256 | 12305 | 4050 | hypothetical protein | OXL23572.1 |
| NEXU01000060 | 12302 | 13282 | 981 | type VI secretion-associated protein | OXL23573.1 |
| NEXU01000060 | 13417 | 14253 | 837 | hypothetical protein | OXL23574.1 |
| NEXU01000060 | 14332 | 17022 | 2691 | hypothetical protein | OXL23575.1 |
| NEXU01000060 | 17015 | 17914 | 900 | hypothetical protein | OXL23576.1 |
| NEXU01000060 | 17911 | 18588 | 678 | hypothetical protein | OXL23577.1 |
| NEXU01000060 | 18608 | 18997 | 390 | hypothetical protein | OXL23578.1 |
| NEXU01000061 | 48 | 872 | 825 | lysophospholipase | OXL23565.1 |
| NEXU01000061 | 1021 | 1842 | 822 | 2,3,4,5-tetrahydropyridine-2,6-dicarboxylate N-succinyltransferase | OXL23558.1 |
| NEXU01000061 | 2094 | 2816 | 723 | 7-carboxy-7-deazaguanine synthase QueE | OXL23559.1 |
| NEXU01000061 | 2903 | 4063 | 1161 | hypothetical protein | OXL23560.1 |
| NEXU01000061 | 4079 | 4714 | 636 | hypothetical protein | OXL23561.1 |
| NEXU01000061 | 4992 | 5609 | 618 | hypothetical protein | OXL23566.1 |
| NEXU01000061 | 7552 | 5714 | 1839 | single-stranded-DNA-specific exonuclease RecJ | OXL23562.1 |
| NEXU01000061 | 7879 | 8394 | 516 | hypothetical protein | OXL23563.1 |
| NEXU01000061 | 9599 | 8541 | 1059 | hypothetical protein | OXL23564.1 |
| NEXU01000062 | 265 | <1 | N/D | hypothetical protein | OXL23462.1 |
| NEXU01000062 | 1113 | 340 | 774 | ABC transporter permease | OXL23468.1 |
| NEXU01000062 | 2132 | 1170 | 963 | ABC transporter | OXL23463.1 |
| NEXU01000062 | 3167 | 2526 | 642 | cytochrome c5 family protein | OXL23464.1 |
| NEXU01000062 | 3558 | 3634 | 77 | tRNA-Arg |  |
| NEXU01000062 | 4284 | 3919 | 366 | hypothetical protein | OXL23465.1 |
| NEXU01000062 | 4320 | 5120 | 801 | NAD-dependent protein deacylase | OXL23466.1 |
| NEXU01000062 | >5708 | 5277 | N/D | adenosine deaminase | OXL23467.1 |
| NEXU01000063 | 373 | <1 | N/D | adenosine deaminase | OXL23363.1 |
| NEXU01000063 | 1002 | 478 | 525 | hypothetical protein | OXL23364.1 |
| NEXU01000063 | 2229 | 1054 | 1176 | methanol dehydrogenase | OXL23365.1 |
| NEXU01000063 | 3182 | 2553 | 630 | hypothetical protein | OXL23366.1 |
| NEXU01000063 | 4117 | 5664 | 1548 | sodium:alanine symporter | OXL23367.1 |
| NEXU01000063 | 6651 | 6043 | 609 | beta-Ig-H3/fasciclin | OXL23368.1 |
| NEXU01000063 | 7070 | 6828 | 243 | hypothetical protein | OXL23369.1 |
| NEXU01000063 | 7631 | 7077 | 555 | beta-Ig-H3/fasciclin | OXL23370.1 |
| NEXU01000063 | 8394 | 12209 | 3816 | methionine synthase | OXL23396.1 |
| NEXU01000063 | 12409 | 13338 | 930 | sodium transporter | OXL23371.1 |
| NEXU01000063 | 15165 | 13594 | 1572 | hypothetical protein | OXL23372.1 |
| NEXU01000063 | 15348 | 15196 | 153 | hypothetical protein | OXL23373.1 |
| NEXU01000063 | 15564 | 15394 | 171 | hypothetical protein | OXL23374.1 |
| NEXU01000063 | 21712 | 15620 | 6093 | DNA repair protein | OXL23375.1 |
| NEXU01000063 | 24046 | 22088 | 1959 | hypothetical protein | OXL23397.1 |
| NEXU01000063 | 25009 | 24347 | 663 | hypothetical protein | OXL23376.1 |
| NEXU01000063 | 26320 | 25307 | 1014 | Xaa-Pro aminopeptidase | OXL23398.1 |
| NEXU01000063 | 26981 | 26493 | 489 | META domain-containing protein | OXL23377.1 |
| NEXU01000063 | 28407 | 27298 | 1110 | lipase chaperone | OXL23378.1 |
| NEXU01000063 | 29640 | 28552 | 1089 | alpha/beta hydrolase | OXL23379.1 |
| NEXU01000063 | 30052 | 30846 | 795 | type I methionyl aminopeptidase | OXL23380.1 |
| NEXU01000063 | 31017 | 33812 | 2796 | [protein-PII] uridylyltransferase | OXL23381.1 |
| NEXU01000063 | 34615 | 33947 | 669 | hypothetical protein | OXL23382.1 |
| NEXU01000063 | 34879 | 36111 | 1233 | succinyldiaminopimelate transaminase | OXL23383.1 |
| NEXU01000063 | 36333 | 38285 | 1953 | nucleotidyltransferase | OXL23384.1 |
| NEXU01000063 | 38318 | 38923 | 606 | DNA polymerase III subunit epsilon | OXL23385.1 |
| NEXU01000063 | 39059 | 41170 | 2112 | D-alanyl-D-alanine carboxypeptidase | OXL23399.1 |
| NEXU01000063 | 41279 | 41794 | 516 | hypothetical protein | OXL23386.1 |
| NEXU01000063 | 41932 | 42480 | 549 | hypothetical protein | OXL23387.1 |
| NEXU01000063 | 43156 | 42710 | 447 | signal peptidase | OXL23388.1 |
| NEXU01000063 | 43460 | 43948 | 489 | 23S rRNA (pseudouridine(1915)-N(3))-methyltransferase RlmH | OXL23389.1 |
| NEXU01000063 | 44034 | 44948 | 915 | dioxygenase | OXL23390.1 |
| NEXU01000063 | 46508 | 45015 | 1494 | amidase | OXL23391.1 |
| NEXU01000063 | 50153 | 46770 | 3384 | cell division protein FtsK | OXL23392.1 |
| NEXU01000063 | 50866 | 51888 | 1023 | thioredoxin-disulfide reductase | OXL23393.1 |
| NEXU01000063 | 51919 | 52737 | 819 | leucyl/phenylalanyl-tRNA--protein transferase | OXL23400.1 |
| NEXU01000063 | 52832 | 53266 | 435 | MerR family transcriptional regulator | OXL23394.1 |
| NEXU01000063 | 53809 | 53333 | 477 | ribosomal-protein-alanine N-acetyltransferase | OXL23395.1 |
| NEXU01000064 | 759 | <1 | N/D | elongation factor Tu | OXL23333.1 |
| NEXU01000064 | 1339 | 1265 | 75 | tRNA-Thr |  |
| NEXU01000064 | 1518 | 1445 | 74 | tRNA-Gly |  |
| NEXU01000064 | 1687 | 1604 | 84 | tRNA-Tyr |  |
| NEXU01000064 | 1828 | 1753 | 76 | tRNA-Thr |  |
| NEXU01000064 | 3367 | 2372 | 996 | peptide-binding protein | OXL23334.1 |
| NEXU01000064 | 4304 | 3450 | 855 | DUF3298 domain-containing protein | OXL23335.1 |
| NEXU01000064 | 6109 | 4514 | 1596 | peptide chain release factor 3 | OXL23336.1 |
| NEXU01000064 | 7283 | 6360 | 924 | hypothetical protein | OXL23337.1 |
| NEXU01000064 | 7570 | 7983 | 414 | peroxiredoxin | OXL23338.1 |
| NEXU01000064 | 8197 | 11175 | 2979 | DNA polymerase I | OXL23356.1 |
| NEXU01000064 | 11364 | 11891 | 528 | hypothetical protein | OXL23339.1 |
| NEXU01000064 | 12172 | 12960 | 789 | short-chain dehydrogenase | OXL23340.1 |
| NEXU01000064 | 13247 | 13585 | 339 | hypothetical protein | OXL23341.1 |
| NEXU01000064 | 14723 | 13704 | 1020 | ferrochelatase | OXL23342.1 |
| NEXU01000064 | 15104 | 16489 | 1386 | homoserine O-acetyltransferase | OXL23343.1 |
| NEXU01000064 | 16486 | 17103 | 618 | methionine biosynthesis protein MetW | OXL23344.1 |
| NEXU01000064 | 17492 | 18073 | 582 | hypothetical protein | OXL23345.1 |
| NEXU01000064 | 18325 | 19383 | 1059 | hypothetical protein | OXL23346.1 |
| NEXU01000064 | 20003 | 19470 | 534 | DNA repair protein | OXL23347.1 |
| NEXU01000064 | 20677 | 20294 | 384 | hydroxyisourate hydrolase | OXL23357.1 |
| NEXU01000064 | 22248 | 21022 | 1227 | D-3-phosphoglycerate dehydrogenase | OXL23348.1 |
| NEXU01000064 | 22636 | 24078 | 1443 | FAD-binding oxidoreductase | OXL23349.1 |
| NEXU01000064 | 24865 | 24239 | 627 | blue light sensor protein | OXL23350.1 |
| NEXU01000064 | 25396 | 27033 | 1638 | hydrolase | OXL23351.1 |
| NEXU01000064 | 27166 | 27717 | 552 | protein disulfide oxidoreductase | OXL23352.1 |
| NEXU01000064 | 28920 | 27805 | 1116 | alanine dehydrogenase | OXL23353.1 |
| NEXU01000064 | 29590 | 32448 | 2859 | isoleucine--tRNA ligase | OXL23354.1 |
| NEXU01000064 | 32441 | >32813 | N/D | signal peptidase II | OXL23355.1 |
| NEXU01000065 | 124 | 627 | 504 | signal peptidase II |  |
| NEXU01000065 | 1394 | 735 | 660 | DNA-binding protein | OXL23274.1 |
| NEXU01000065 | 1542 | 2039 | 498 | peptidylprolyl isomerase | OXL23275.1 |
| NEXU01000065 | 2082 | 3122 | 1041 | aldo/keto reductase | OXL23276.1 |
| NEXU01000065 | 4069 | 3269 | 801 | 1-acyl-sn-glycerol-3-phosphate acyltransferase | OXL23277.1 |
| NEXU01000065 | 4437 | 5924 | 1488 | GTPase HflX | OXL23278.1 |
| NEXU01000065 | 6384 | 6818 | 435 | CidA/LrgA family protein | OXL23304.1 |
| NEXU01000065 | 6841 | 7530 | 690 | rhamnonate dehydratase | OXL23279.1 |
| NEXU01000065 | 8121 | 8945 | 825 | lytic transglycosylase | OXL23280.1 |
| NEXU01000065 | 10019 | 9135 | 885 | translation elongation factor Ts | OXL23281.1 |
| NEXU01000065 | 11043 | 10246 | 798 | 30S ribosomal protein S2 | OXL23282.1 |
| NEXU01000065 | 11864 | 11415 | 450 | chemotaxis protein CheX | OXL23283.1 |
| NEXU01000065 | 12363 | 11914 | 450 | chemotaxis protein CheX | OXL23284.1 |
| NEXU01000065 | 12772 | 12410 | 363 | response regulator | OXL23285.1 |
| NEXU01000065 | 14994 | 12793 | 2202 | chemotaxis protein CheA | OXL23305.1 |
| NEXU01000065 | 16954 | 15695 | 1260 | Bcr/CflA family drug resistance efflux transporter | OXL23286.1 |
| NEXU01000065 | 18029 | 17217 | 813 | enoyl-CoA hydratase | OXL23287.1 |
| NEXU01000065 | 21669 | 18163 | 3507 | hybrid sensor histidine kinase/response regulator | OXL23288.1 |
| NEXU01000065 | 22146 | 23114 | 969 | cysteine synthase B | OXL23289.1 |
| NEXU01000065 | 23139 | 23963 | 825 | 3'-5' exonuclease | OXL23290.1 |
| NEXU01000065 | 24043 | 25584 | 1542 | 23S rRNA (uracil(1939)-C(5))-methyltransferase | OXL23291.1 |
| NEXU01000065 | 25979 | 28531 | 2553 | GTP pyrophosphokinase | OXL23292.1 |
| NEXU01000065 | 28598 | 29581 | 984 | DNA-formamidopyrimidine glycosylase | OXL23293.1 |
| NEXU01000065 | 29803 | 30243 | 441 | hypothetical protein | OXL23294.1 |
| NEXU01000065 | 30848 | 30405 | 444 | large-conductance mechanosensitive channel protein | OXL23295.1 |
| NEXU01000065 | 32997 | 31150 | 1848 | translational GTPase TypA | OXL23296.1 |
| NEXU01000065 | 34522 | 33353 | 1170 | hypothetical protein | OXL23297.1 |
| NEXU01000065 | 35127 | 35714 | 588 | general secretion pathway protein GspH | OXL23298.1 |
| NEXU01000065 | 36534 | 35734 | 801 | pyridoxine 5'-phosphate synthase | OXL23299.1 |
| NEXU01000065 | 37403 | 36600 | 804 | DNA recombination protein RecO | OXL23300.1 |
| NEXU01000065 | 38424 | 37465 | 960 | GTPase Era | OXL23306.1 |
| NEXU01000065 | 39668 | 38871 | 798 | ribonuclease III | OXL23301.1 |
| NEXU01000065 | 40235 | 39840 | 396 | DUF4845 domain-containing protein | OXL23302.1 |
| NEXU01000065 | 41298 | 40396 | 903 | signal peptidase I | OXL23303.1 |
| NEXU01000065 | >42096 | 41379 | N/D | elongation factor 4 | OXL23307.1 |
| NEXU01000066 | 968 | <1 | N/D | elongation factor 4 | OXL23154.1 |
| NEXU01000066 | 1462 | 1386 | 77 | tRNA-Met |  |
| NEXU01000066 | 2781 | 2062 | 720 | dethiobiotin synthase | OXL23155.1 |
| NEXU01000066 | 4115 | 2778 | 1338 | adenosylmethionine--8-amino-7-oxononanoate transaminase | OXL23156.1 |
| NEXU01000066 | 4367 | 4119 | 249 | ferredoxin | OXL23157.1 |
| NEXU01000066 | 4958 | 4449 | 510 | pantetheine-phosphate adenylyltransferase | OXL23158.1 |
| NEXU01000066 | 5211 | 5684 | 474 | SsrA-binding protein | OXL23159.1 |
| NEXU01000066 | 6829 | 5807 | 1023 | protein RarD | OXL23160.1 |
| NEXU01000066 | 7073 | 7540 | 468 | AsnC family transcriptional regulator | OXL23161.1 |
| NEXU01000066 | 7899 | 8657 | 759 | hypothetical protein | OXL23162.1 |
| NEXU01000066 | 8889 | 11330 | 2442 | YgiQ family radical SAM protein | OXL23163.1 |
| NEXU01000066 | 11691 | 12101 | 411 | hypothetical protein | OXL23164.1 |
| NEXU01000066 | 12362 | 12892 | 531 | hypothetical protein | OXL23165.1 |
| NEXU01000066 | 14462 | 13041 | 1422 | hypothetical protein | OXL23166.1 |
| NEXU01000066 | 16588 | 14486 | 2103 | DNA ligase (NAD(+)) LigA | OXL23167.1 |
| NEXU01000066 | 17943 | 16918 | 1026 | cell division protein | OXL23168.1 |
| NEXU01000066 | 22094 | 18150 | 3945 | chromosome segregation protein SMC | OXL23169.1 |
| NEXU01000066 | 23102 | 22167 | 936 | hypothetical protein | OXL23170.1 |
| NEXU01000066 | 23961 | 23155 | 807 | hypothetical protein | OXL23171.1 |
| NEXU01000066 | 24163 | 25152 | 990 | biotin biosynthesis protein BioC | OXL23172.1 |
| NEXU01000066 | 25190 | 25918 | 729 | pantothenate kinase | OXL23173.1 |
| NEXU01000066 | 27193 | 26006 | 1188 | acyl-CoA dehydrogenase | OXL23174.1 |
| NEXU01000066 | 28338 | 27418 | 921 | hydroxymethylglutaryl-CoA lyase | OXL23175.1 |
| NEXU01000066 | 30572 | 28419 | 2154 | 3-methylcrotonyl-CoA carboxylase | OXL23176.1 |
| NEXU01000066 | 31656 | 30775 | 882 | enoyl-CoA hydratase | OXL23177.1 |
| NEXU01000066 | 33359 | 31749 | 1611 | methylcrotonoyl-CoA carboxylase | OXL23178.1 |
| NEXU01000066 | 35226 | 33469 | 1758 | AMP-binding protein | OXL23179.1 |
| NEXU01000066 | 36826 | 35582 | 1245 | pyridine nucleotide-disulfide oxidoreductase | OXL23180.1 |
| NEXU01000066 | 37830 | 37012 | 819 | amino acid ABC transporter ATP-binding protein | OXL23181.1 |
| NEXU01000066 | 38584 | 37913 | 672 | amino acid ABC transporter permease | OXL23182.1 |
| NEXU01000066 | 39327 | 38581 | 747 | amino acid ABC transporter permease | OXL23183.1 |
| NEXU01000066 | 40431 | 39436 | 996 | amino acid ABC transporter substrate-binding protein | OXL23184.1 |
| NEXU01000066 | 40811 | 41791 | 981 | heat-shock protein Hsp33 | OXL23185.1 |
| NEXU01000067 | 1527 | 28 | 1500 | sodium/proline symporter | OXL23110.1 |
| NEXU01000067 | 2909 | 2037 | 873 | LysR family transcriptional regulator | OXL23111.1 |
| NEXU01000067 | 3209 | 4627 | 1419 | 3-isopropylmalate dehydratase large subunit | OXL23112.1 |
| NEXU01000067 | 4707 | 5357 | 651 | 3-isopropylmalate dehydratase small subunit | OXL23113.1 |
| NEXU01000067 | 5557 | 6306 | 750 | hypothetical protein | OXL23114.1 |
| NEXU01000067 | 6516 | 7619 | 1104 | 3-isopropylmalate dehydrogenase | OXL23115.1 |
| NEXU01000067 | 8136 | 9263 | 1128 | murein L,D-transpeptidase | OXL23116.1 |
| NEXU01000067 | 9760 | 9539 | 222 | translation initiation factor IF-1 | OXL23117.1 |
| NEXU01000067 | 10684 | 9884 | 801 | tRNA pseudouridine(38-40) synthase TruA | OXL23146.1 |
| NEXU01000067 | 11924 | 10839 | 1086 | L-asparaginase 1 | OXL23147.1 |
| NEXU01000067 | 13648 | 12086 | 1563 | pilus assembly protein FimV | OXL23118.1 |
| NEXU01000067 | 15520 | 13700 | 1821 | peptigoglycan-binding protein LysM | OXL23119.1 |
| NEXU01000067 | 16858 | 15638 | 1221 | tRNA preQ1(34) S-adenosylmethionine ribosyltransferase-isomerase QueA | OXL23120.1 |
| NEXU01000067 | 18119 | 17034 | 1086 | DNA polymerase IV | OXL23121.1 |
| NEXU01000067 | 18673 | 18918 | 246 | hypothetical protein | OXL23122.1 |
| NEXU01000067 | 19649 | 19002 | 648 | Gluthatione S-transferase | OXL23123.1 |
| NEXU01000067 | 19786 | 20670 | 885 | IclR family transcriptional regulator | OXL23124.1 |
| NEXU01000067 | 21305 | 20730 | 576 | glyoxalase | OXL23125.1 |
| NEXU01000067 | 22741 | 21440 | 1302 | fumarylacetoacetase | OXL23126.1 |
| NEXU01000067 | 24204 | 23008 | 1197 | aromatic amino acid aminotransferase | OXL23127.1 |
| NEXU01000067 | 25482 | 24541 | 942 | LysR family transcriptional regulator | OXL23148.1 |
| NEXU01000067 | 25637 | 26737 | 1101 | 4-hydroxyphenylpyruvate dioxygenase | OXL23128.1 |
| NEXU01000067 | 26950 | 27786 | 837 | phenylalanine 4-monooxygenase | OXL23129.1 |
| NEXU01000067 | 28039 | 28380 | 342 | 4a-hydroxytetrahydrobiopterin dehydratase | OXL23130.1 |
| NEXU01000067 | 28472 | 29896 | 1425 | Na+/H+ antiporter NhaC | OXL23131.1 |
| NEXU01000067 | 30225 | 30929 | 705 | hypothetical protein | OXL23132.1 |
| NEXU01000067 | 31258 | 31172 | 87 | tRNA-Leu |  |
| NEXU01000067 | 32169 | 31396 | 774 | ferredoxin--NADP(+) reductase | OXL23133.1 |
| NEXU01000067 | 32596 | 33528 | 933 | tRNA (N6-threonylcarbamoyladenosine(37)-N6)-methyltransferase TrmO | OXL23134.1 |
| NEXU01000067 | 33587 | 35374 | 1788 | type II secretion system protein E | OXL23135.1 |
| NEXU01000067 | 35524 | 36312 | 789 | TatD family deoxyribonuclease | OXL23136.1 |
| NEXU01000067 | 36546 | 37229 | 684 | TetR family transcriptional regulator | OXL23137.1 |
| NEXU01000067 | 37407 | 38090 | 684 | prepilin-type cleavage/methylation domain-containing protein | OXL23138.1 |
| NEXU01000067 | 38094 | 38573 | 480 | type II secretion system protein GspI | OXL23139.1 |
| NEXU01000067 | 38570 | 39454 | 885 | type II secretion system protein GspJ | OXL23140.1 |
| NEXU01000067 | 39488 | 40588 | 1101 | general secretion pathway protein GspK | OXL23141.1 |
| NEXU01000067 | 41343 | 40663 | 681 | TetR family transcriptional regulator | OXL23142.1 |
| NEXU01000067 | 42847 | 41426 | 1422 | deoxyguanosinetriphosphate triphosphohydrolase | OXL23143.1 |
| NEXU01000067 | 44171 | 43080 | 1092 | redox-regulated ATPase YchF | OXL23144.1 |
| NEXU01000067 | 44526 | >45526 | N/D | DNA helicase UvrD | OXL23145.1 |
| NEXU01000068 | <1 | 702 | N/D | DNA helicase UvrD | OXL23018.1 |
| NEXU01000068 | 703 | 1704 | 1002 | hypothetical protein | OXL23019.1 |
| NEXU01000068 | 6464 | 1821 | 4644 | hypothetical protein | OXL23020.1 |
| NEXU01000068 | 6900 | 8147 | 1248 | amino acid dehydrogenase | OXL23021.1 |
| NEXU01000068 | 8168 | 8506 | 339 | hypothetical protein | OXL23022.1 |
| NEXU01000068 | 8756 | 9187 | 432 | Replicative DNA helicase | OXL23023.1 |
| NEXU01000068 | 10153 | 9317 | 837 | enoyl-CoA hydratase | OXL23024.1 |
| NEXU01000068 | 11467 | 10331 | 1137 | glycerate kinase | OXL23025.1 |
| NEXU01000068 | 11733 | 11539 | 195 | hypothetical protein | OXL23026.1 |
| NEXU01000068 | 11808 | 12143 | 336 | hypothetical protein | OXL23027.1 |
| NEXU01000068 | 12380 | 12156 | 225 | hypothetical protein | OXL23028.1 |
| NEXU01000068 | 13218 | 12703 | 516 | 50S ribosomal protein L9 | OXL23029.1 |
| NEXU01000068 | 13471 | 13244 | 228 | 30S ribosomal protein S18 | OXL23030.1 |
| NEXU01000068 | 13889 | 13488 | 402 | 30S ribosomal protein S6 | OXL23031.1 |
| NEXU01000068 | 15174 | 14176 | 999 | hypothetical protein | OXL23032.1 |
| NEXU01000068 | 17715 | 15331 | 2385 | phosphoenolpyruvate synthase | OXL23033.1 |
| NEXU01000068 | 18303 | 19199 | 897 | phosphoenolpyruvate synthase regulatory protein | OXL23034.1 |
| NEXU01000068 | 19384 | 21024 | 1641 | NAD(P)(+) transhydrogenase | OXL23035.1 |
| NEXU01000068 | 22294 | 21101 | 1194 | ABC transporter | OXL23036.1 |
| NEXU01000068 | 23589 | 22294 | 1296 | beta-carotene 15,15'-monooxygenase | OXL23037.1 |
| NEXU01000068 | 24801 | 23593 | 1209 | secretion protein HlyD | OXL23038.1 |
| NEXU01000068 | 26610 | 24925 | 1686 | transporter | OXL23039.1 |
| NEXU01000069 | 193 | 1794 | 1602 | glycerol kinase | OXL23001.1 |
| NEXU01000069 | 2075 | 2752 | 678 | hypothetical protein | OXL23002.1 |
| NEXU01000069 | 3059 | 4084 | 1026 | endonuclease | OXL23003.1 |
| NEXU01000069 | 4299 | 4991 | 693 | TetR family transcriptional regulator | OXL23004.1 |
| NEXU01000069 | 5884 | 5111 | 774 | hypothetical protein | OXL23005.1 |
| NEXU01000069 | 6409 | 5945 | 465 | hypothetical protein | OXL23006.1 |
| NEXU01000070 | 646 | 3468 | 2823 | pyruvate dehydrogenase (acetyl-transferring), homodimeric type | OXL22996.1 |
| NEXU01000070 | 3491 | 5233 | 1743 | pyruvate dehydrogenase complex dihydrolipoyllysine-residue acetyltransferase | OXL22997.1 |
| NEXU01000070 | 6061 | 5402 | 660 | hypothetical protein | OXL22998.1 |
| NEXU01000070 | 6355 | 6582 | 228 | hypothetical protein | OXL22999.1 |
| NEXU01000070 | 6888 | 7868 | 981 | Ion transport protein | OXL23000.1 |
| NEXU01000071 | 659 | <1 | N/D | hypothetical protein | OXL22958.1 |
| NEXU01000071 | 1877 | 756 | 1122 | WYL domain-containing protein | OXL22959.1 |
| NEXU01000071 | 4803 | 1996 | 2808 | peptidase S16 | OXL22960.1 |
| NEXU01000071 | 5166 | 5885 | 720 | 16S rRNA (guanine(527)-N(7))-methyltransferase RsmG | OXL22961.1 |
| NEXU01000071 | 6105 | 6884 | 780 | cobalamin biosynthesis protein CobQ | OXL22962.1 |
| NEXU01000071 | 7053 | 8189 | 1137 | chromosome partitioning protein ParB | OXL22963.1 |
| NEXU01000071 | 8269 | 9000 | 732 | biopolymer transporter ExbB | OXL22964.1 |
| NEXU01000071 | 9069 | 9500 | 432 | biopolymer transporter ExbD | OXL22965.1 |
| NEXU01000071 | 9650 | 11419 | 1770 | lipid A export permease/ATP-binding protein MsbA | OXL22966.1 |
| NEXU01000071 | 11503 | 12642 | 1140 | tetraacyldisaccharide 4'-kinase | OXL22967.1 |
| NEXU01000071 | 12686 | 13492 | 807 | 3-deoxy-D-manno-octulosonate cytidylyltransferase | OXL22968.1 |
| NEXU01000071 | 13623 | 14741 | 1119 | DNA polymerase III subunit delta' | OXL22991.1 |
| NEXU01000071 | 14869 | 15216 | 348 | pilus assembly protein PilZ | OXL22969.1 |
| NEXU01000071 | 16287 | 15370 | 918 | polyphosphate kinase 2 | OXL22992.1 |
| NEXU01000071 | 18245 | 16647 | 1599 | acetyl-CoA acetyltransferase | OXL22970.1 |
| NEXU01000071 | 18841 | 20241 | 1401 | 3-oxoacyl-ACP reductase | OXL22971.1 |
| NEXU01000071 | 20574 | 21488 | 915 | acyl dehydratase | OXL22972.1 |
| NEXU01000071 | 21540 | 22091 | 552 | serine/threonine protein phosphatase | OXL22973.1 |
| NEXU01000071 | 22229 | 23713 | 1485 | serine hydrolase | OXL22974.1 |
| NEXU01000071 | 25011 | 23761 | 1251 | sodium:proton antiporter | OXL22975.1 |
| NEXU01000071 | 27231 | 25321 | 1911 | dihydroxy-acid dehydratase | OXL22976.1 |
| NEXU01000071 | 28194 | 27412 | 783 | hypothetical protein | OXL22977.1 |
| NEXU01000071 | 28412 | 28867 | 456 | HIT family protein | OXL22978.1 |
| NEXU01000071 | 29241 | 31289 | 2049 | Choline-glycine betaine transporter, BCCT family | OXL22979.1 |
| NEXU01000071 | 31369 | 32400 | 1032 | tRNA dihydrouridine synthase DusB | OXL22980.1 |
| NEXU01000071 | 32568 | 33455 | 888 | fatty acyl-CoA reductase | OXL22981.1 |
| NEXU01000071 | 34039 | 33716 | 324 | hypothetical protein | OXL22982.1 |
| NEXU01000071 | 34741 | 34268 | 474 | hypothetical protein | OXL22983.1 |
| NEXU01000071 | 35478 | 34942 | 537 | hypothetical protein | OXL22984.1 |
| NEXU01000071 | 36006 | 35503 | 504 | hypothetical protein | OXL22985.1 |
| NEXU01000071 | 36674 | 36210 | 465 | hypothetical protein | OXL22986.1 |
| NEXU01000071 | 37407 | 36877 | 531 | inorganic pyrophosphatase | OXL22987.1 |
| NEXU01000071 | 38466 | 37612 | 855 | octanoyltransferase | OXL22988.1 |
| NEXU01000071 | 38881 | 40368 | 1488 | coniferyl aldehyde dehydrogenase | OXL22989.1 |
| NEXU01000071 | 40773 | 42704 | 1932 | ABC-type proline/glycine betaine transporter | OXL22993.1 |
| NEXU01000071 | 42797 | 43669 | 873 | ABC-type proline/glycine betaine transporter | OXL22990.1 |
| NEXU01000072 | 271 | 181 | 91 | tRNA-Ser |  |
| NEXU01000072 | 474 | 1382 | 909 | endonuclease | OXL22799.1 |
| NEXU01000072 | 1457 | 1720 | 264 | hypothetical protein | OXL22800.1 |
| NEXU01000072 | 1994 | 2377 | 384 | hypothetical protein | OXL22801.1 |
| NEXU01000072 | 3792 | 2530 | 1263 | ammonium transporter | OXL22802.1 |
| NEXU01000072 | 4221 | 3883 | 339 | transcriptional regulator | OXL22803.1 |
| NEXU01000072 | 5506 | 4652 | 855 | universal stress protein | OXL22804.1 |
| NEXU01000072 | 7156 | 5657 | 1500 | sodium-independent anion transporter | OXL22805.1 |
| NEXU01000072 | 8039 | 9454 | 1416 | UDP-N-acetylmuramoyl-L-alanine--D-glutamate ligase | OXL22806.1 |
| NEXU01000072 | 9851 | 10975 | 1125 | putative lipid II flippase FtsW | OXL22807.1 |
| NEXU01000072 | 12110 | 11112 | 999 | tRNA glutamyl-Q(34) synthetase GluQRS | OXL22808.1 |
| NEXU01000072 | 12816 | 12376 | 441 | RNA polymerase-binding protein DksA | OXL22809.1 |
| NEXU01000072 | 14195 | 13362 | 834 | 3',5'-cyclic-nucleotide phosphodiesterase | OXL22810.1 |
| NEXU01000072 | 15627 | 14794 | 834 | DNA repair protein | OXL22811.1 |
| NEXU01000072 | 16455 | 15700 | 756 | zinc ABC transporter ATP-binding protein | OXL22886.1 |
| NEXU01000072 | 17136 | 16615 | 522 | Fur family transcriptional regulator | OXL22812.1 |
| NEXU01000072 | 17415 | 18320 | 906 | zinc ABC transporter substrate-binding protein | OXL22813.1 |
| NEXU01000072 | 18690 | 19088 | 399 | hypothetical protein | OXL22814.1 |
| NEXU01000072 | 19760 | 20626 | 867 | F0F1 ATP synthase subunit A | OXL22815.1 |
| NEXU01000072 | 20660 | 20908 | 249 | F0F1 ATP synthase subunit C | OXL22816.1 |
| NEXU01000072 | 21026 | 21496 | 471 | F0F1 ATP synthase subunit B | OXL22817.1 |
| NEXU01000072 | 21510 | 22133 | 624 | F0F1 ATP synthase subunit delta | OXL22818.1 |
| NEXU01000072 | 22224 | 23768 | 1545 | F0F1 ATP synthase subunit alpha | OXL22819.1 |
| NEXU01000072 | 24022 | 24903 | 882 | F0F1 ATP synthase subunit gamma | OXL22820.1 |
| NEXU01000072 | 24954 | 26387 | 1434 | F0F1 ATP synthase subunit beta | OXL22821.1 |
| NEXU01000072 | 26503 | 26919 | 417 | F0F1 ATP synthase subunit epsilon | OXL22822.1 |
| NEXU01000072 | 27558 | 28274 | 717 | DNA-binding response regulator | OXL22823.1 |
| NEXU01000072 | 28604 | 30289 | 1686 | two-component sensor histidine kinase | OXL22824.1 |
| NEXU01000072 | 30371 | 30550 | 180 | hypothetical protein | OXL22825.1 |
| NEXU01000072 | 32090 | 30621 | 1470 | two-component sensor histidine kinase | OXL22826.1 |
| NEXU01000072 | 32371 | 32640 | 270 | 30S ribosomal protein S16 | OXL22827.1 |
| NEXU01000072 | 32832 | 33365 | 534 | ribosome maturation factor RimM | OXL22828.1 |
| NEXU01000072 | 33503 | 34267 | 765 | tRNA (guanosine(37)-N1)-methyltransferase TrmD | OXL22829.1 |
| NEXU01000072 | 34457 | 34849 | 393 | 50S ribosomal protein L19 | OXL22830.1 |
| NEXU01000072 | 35688 | 35131 | 558 | DUF4442 domain-containing protein | OXL22831.1 |
| NEXU01000072 | 36182 | 37702 | 1521 | hypothetical protein | OXL22832.1 |
| NEXU01000072 | 37844 | 39688 | 1845 | excinuclease ABC subunit C | OXL22833.1 |
| NEXU01000072 | 40351 | 39779 | 573 | dihydrofolate reductase | OXL22834.1 |
| NEXU01000072 | 41303 | 40416 | 888 | thymidylate synthase | OXL22835.1 |
| NEXU01000072 | 42261 | 41377 | 885 | prolipoprotein diacylglyceryl transferase | OXL22836.1 |
| NEXU01000072 | 42531 | 44219 | 1689 | hypothetical protein | OXL22837.1 |
| NEXU01000072 | 44250 | 45062 | 813 | hypothetical protein | OXL22838.1 |
| NEXU01000072 | 45674 | 45153 | 522 | RNA pyrophosphohydrolase | OXL22839.1 |
| NEXU01000072 | 46178 | 46591 | 414 | osmotically inducible protein OsmC | OXL22840.1 |
| NEXU01000072 | 46606 | 47031 | 426 | hypothetical protein | OXL22841.1 |
| NEXU01000072 | 48074 | 47082 | 993 | tRNA dihydrouridine(16) synthase DusC | OXL22842.1 |
| NEXU01000072 | 48326 | 48913 | 588 | hypothetical protein | OXL22843.1 |
| NEXU01000072 | 50451 | 48952 | 1500 | DNA helicase | OXL22844.1 |
| NEXU01000072 | 51572 | 50703 | 870 | twin-arginine translocase subunit TatC | OXL22845.1 |
| NEXU01000072 | 52294 | 51572 | 723 | twin-arginine translocase subunit TatB | OXL22846.1 |
| NEXU01000072 | 52599 | 52330 | 270 | twin-arginine translocase subunit TatA | OXL22847.1 |
| NEXU01000072 | 53694 | 52828 | 867 | phosphoribosyl-ATP diphosphatase | OXL22848.1 |
| NEXU01000072 | 54217 | 54414 | 198 | 50S ribosomal protein L35 | OXL22849.1 |
| NEXU01000072 | 54544 | 54900 | 357 | 50S ribosomal protein L20 | OXL22850.1 |
| NEXU01000072 | 55272 | 56327 | 1056 | phenylalanine--tRNA ligase subunit alpha | OXL22851.1 |
| NEXU01000072 | 56396 | 58810 | 2415 | phenylalanine--tRNA ligase subunit beta | OXL22852.1 |
| NEXU01000072 | 59086 | 59385 | 300 | integration host factor subunit alpha | OXL22853.1 |
| NEXU01000072 | 59963 | 59604 | 360 | hypothetical protein | OXL22854.1 |
| NEXU01000072 | 60638 | 61525 | 888 | neutral zinc metallopeptidase | OXL22855.1 |
| NEXU01000072 | 61945 | 61697 | 249 | hypothetical protein | OXL22856.1 |
| NEXU01000072 | 63558 | 62290 | 1269 | transcription termination factor Rho | OXL22857.1 |
| NEXU01000072 | 64447 | 64121 | 327 | thioredoxin | OXL22858.1 |
| NEXU01000072 | 64789 | 65502 | 714 | RNA pseudouridine synthase | OXL22859.1 |
| NEXU01000072 | 65700 | 67382 | 1683 | FAD-containing monooxygenase EthA | OXL22860.1 |
| NEXU01000072 | 68362 | 67427 | 936 | hydroxyacid dehydrogenase | OXL22861.1 |
| NEXU01000072 | 69540 | 68572 | 969 | response regulator receiver protein | OXL22862.1 |
| NEXU01000072 | 71925 | 69613 | 2313 | two-component sensor histidine kinase | OXL22863.1 |
| NEXU01000072 | 72545 | 75427 | 2883 | RNA polymerase-binding ATPase | OXL22887.1 |
| NEXU01000072 | 75705 | 76607 | 903 | acyl-CoA thioesterase II | OXL22864.1 |
| NEXU01000072 | 78018 | 76744 | 1275 | A/G-specific adenine glycosylase | OXL22865.1 |
| NEXU01000072 | 79017 | 78085 | 933 | hypothetical protein | OXL22866.1 |
| NEXU01000072 | 79461 | 79069 | 393 | hypothetical protein | OXL22867.1 |
| NEXU01000072 | 80492 | 79536 | 957 | ribosome biogenesis GTPase YlqF | OXL22868.1 |
| NEXU01000072 | 83774 | 80631 | 3144 | hypothetical protein | OXL22869.1 |
| NEXU01000072 | 84665 | 83928 | 738 | dienelactone hydrolase | OXL22870.1 |
| NEXU01000072 | 84840 | 85445 | 606 | hypothetical protein | OXL22871.1 |
| NEXU01000072 | 86919 | 85549 | 1371 | hypothetical protein | OXL22872.1 |
| NEXU01000072 | 87170 | 88069 | 900 | magnesium/cobalt efflux protein | OXL22873.1 |
| NEXU01000072 | 88377 | 89951 | 1575 | apolipoprotein N-acyltransferase | OXL22874.1 |
| NEXU01000072 | 90089 | 90511 | 423 | hypothetical protein | OXL22875.1 |
| NEXU01000072 | 90810 | 92381 | 1572 | MFS transporter | OXL22876.1 |
| NEXU01000072 | 92800 | 94617 | 1818 | Xaa-Pro aminopeptidase | OXL22877.1 |
| NEXU01000072 | 95591 | 94698 | 894 | hypothetical protein | OXL22878.1 |
| NEXU01000072 | 97076 | 95712 | 1365 | beta-ketoacyl-[acyl-carrier-protein] synthase II | OXL22879.1 |
| NEXU01000072 | 98597 | 97173 | 1425 | anhydro-N-acetylmuramic acid kinase | OXL22880.1 |
| NEXU01000072 | 98717 | 99928 | 1212 | tyrosine--tRNA ligase | OXL22881.1 |
| NEXU01000072 | 100052 | 100282 | 231 | hypothetical protein | OXL22882.1 |
| NEXU01000072 | 101165 | 100413 | 753 | NADP-dependent 3-hydroxy acid dehydrogenase | OXL22883.1 |
| NEXU01000072 | 101937 | 101296 | 642 | uracil phosphoribosyltransferase | OXL22884.1 |
| NEXU01000072 | 102306 | 103031 | 726 | DNA glycosylase | OXL22885.1 |
| NEXU01000073 | <1 | 686 | N/D | hypothetical protein | OXL22780.1 |
| NEXU01000073 | 2404 | 800 | 1605 | GMP synthase (glutamine-hydrolyzing) | OXL22781.1 |
| NEXU01000073 | 2920 | 4656 | 1737 | FAD-binding oxidoreductase | OXL22782.1 |
| NEXU01000073 | 4918 | 6576 | 1659 | FAD-dependent oxidoreductase | OXL22783.1 |
| NEXU01000073 | 6756 | 8324 | 1569 | carbohydrate kinase | OXL22784.1 |
| NEXU01000073 | 8392 | 8892 | 501 | hypothetical protein | OXL22785.1 |
| NEXU01000073 | 10081 | 8915 | 1167 | aminotransferase class I/II | OXL22786.1 |
| NEXU01000073 | 12400 | 10583 | 1818 | hypothetical protein | OXL22787.1 |
| NEXU01000073 | 13244 | 12429 | 816 | enoyl-CoA hydratase | OXL22788.1 |
| NEXU01000073 | 13471 | 14508 | 1038 | 23S rRNA (adenine(1618)-N(6))-methyltransferase | OXL22789.1 |
| NEXU01000074 | 1 | 218 | 218 | 23S rRNA (adenine(1618)-N(6))-methyltransferase |  |
| NEXU01000074 | 951 | 343 | 609 | manganese efflux pump MntP | OXL21822.1 |
| NEXU01000074 | 3688 | 1061 | 2628 | DNA topoisomerase (ATP-hydrolyzing) subunit B | OXL21823.1 |
| NEXU01000074 | 5436 | 4222 | 1215 | DNA replication and repair protein RecF | OXL21824.1 |
| NEXU01000074 | 6729 | 5554 | 1176 | DNA polymerase III subunit beta | OXL21825.1 |
| NEXU01000074 | 8336 | 6891 | 1446 | chromosomal replication initiation protein DnaA | OXL21826.1 |
| NEXU01000074 | 9049 | 9183 | 135 | 50S ribosomal protein L34 | OXL21827.1 |
| NEXU01000074 | 9361 | 9732 | 372 | ribonuclease P protein component | OXL21828.1 |
| NEXU01000074 | 9734 | 10120 | 387 | membrane protein insertion efficiency factor YidD | OXL21829.1 |
| NEXU01000074 | 10338 | 12035 | 1698 | membrane protein insertase YidC | OXL21830.1 |
| NEXU01000074 | 12282 | 13730 | 1449 | tRNA uridine-5-carboxymethylaminomethyl(34) synthesis GTPase MnmE | OXL21831.1 |
| NEXU01000074 | 13841 | 14299 | 459 | transcriptional regulator NrdR | OXL21832.1 |
| NEXU01000074 | 14404 | 15459 | 1056 | riboflavin biosynthesis protein RibD | OXL21833.1 |
| NEXU01000074 | 16042 | 16716 | 675 | riboflavin synthase | OXL21834.1 |
| NEXU01000074 | 19795 | 16850 | 2946 | histidine kinase | OXL21835.1 |
| NEXU01000074 | 20042 | 21166 | 1125 | methionyl-tRNA formyltransferase | OXL21836.1 |
| NEXU01000074 | 21242 | 22825 | 1584 | rRNA methyltransferase | OXL21837.1 |
| NEXU01000074 | 22871 | 24292 | 1422 | threonine synthase | OXL21838.1 |
| NEXU01000074 | 24554 | 25687 | 1134 | peptigoglycan-binding protein LysM | OXL21839.1 |
| NEXU01000074 | 25746 | 27014 | 1269 | DNA protecting protein DprA | OXL21858.1 |
| NEXU01000074 | 27142 | 27771 | 630 | tRNA threonylcarbamoyladenosine biosynthesis protein RimN | OXL21840.1 |
| NEXU01000074 | 27897 | 28415 | 519 | MarR family transcriptional regulator | OXL21841.1 |
| NEXU01000074 | 28548 | 29495 | 948 | fatty acid desaturase | OXL21842.1 |
| NEXU01000074 | 29629 | 30126 | 498 | hypothetical protein | OXL21843.1 |
| NEXU01000074 | 30161 | 31150 | 990 | epimerase | OXL21844.1 |
| NEXU01000074 | 31233 | 31694 | 462 | hypothetical protein | OXL21845.1 |
| NEXU01000074 | 31804 | 32877 | 1074 | metallophosphoesterase | OXL21846.1 |
| NEXU01000074 | 33060 | 34226 | 1167 | metallophosphoesterase | OXL21847.1 |
| NEXU01000074 | 35717 | 34239 | 1479 | lipase | OXL21848.1 |
| NEXU01000074 | 36239 | 36853 | 615 | nucleotide exchange factor GrpE | OXL21849.1 |
| NEXU01000074 | 37109 | 39052 | 1944 | molecular chaperone DnaK | OXL21850.1 |
| NEXU01000074 | 39369 | 39911 | 543 | cell envelope biogenesis protein OmpA | OXL21851.1 |
| NEXU01000074 | 40115 | 40690 | 576 | cell envelope biogenesis protein OmpA | OXL21852.1 |
| NEXU01000074 | 40948 | 41445 | 498 | hypothetical protein | OXL21853.1 |
| NEXU01000074 | 41461 | 42321 | 861 | hypothetical protein | OXL21854.1 |
| NEXU01000074 | 42494 | 43870 | 1377 | phytase esterase-like protein | OXL21855.1 |
| NEXU01000074 | 45519 | 43975 | 1545 | oxygen-independent coproporphyrinogen III oxidase | OXL21856.1 |
| NEXU01000074 | 48351 | 45847 | 2505 | peptidase S45 | OXL21857.1 |
| NEXU01000075 | 498 | 2108 | 1611 | ATP-dependent protease | OXL21794.1 |
| NEXU01000075 | 4138 | 2456 | 1683 | dihydroxy-acid dehydratase | OXL21795.1 |
| NEXU01000075 | 4939 | 4262 | 678 | phosphoglycolate phosphatase | OXL21796.1 |
| NEXU01000075 | 5569 | 5069 | 501 | hypothetical protein | OXL21797.1 |
| NEXU01000075 | 5817 | 6107 | 291 | hypothetical protein | OXL21798.1 |
| NEXU01000075 | 6277 | 6202 | 76 | tRNA-Phe |  |
| NEXU01000075 | 6823 | 6401 | 423 | hypothetical protein | OXL21799.1 |
| NEXU01000075 | 7187 | 7840 | 654 | imidazoleglycerol-phosphate dehydratase | OXL21800.1 |
| NEXU01000075 | 7841 | 8485 | 645 | imidazole glycerol phosphate synthase subunit HisH | OXL21801.1 |
| NEXU01000075 | 8604 | 8996 | 393 | hypothetical protein | OXL21802.1 |
| NEXU01000075 | 10374 | 9043 | 1332 | valine--pyruvate transaminase | OXL21803.1 |
| NEXU01000075 | 11091 | 10618 | 474 | hypothetical protein | OXL21804.1 |
| NEXU01000075 | 11472 | 12176 | 705 | hypothetical protein | OXL21805.1 |
| NEXU01000075 | 12981 | 12268 | 714 | hypothetical protein | OXL21806.1 |
| NEXU01000075 | 15704 | 13017 | 2688 | ABC transporter substrate-binding protein | OXL21807.1 |
| NEXU01000075 | 16411 | 15704 | 708 | ABC transporter | OXL21808.1 |
| NEXU01000075 | 17250 | 16408 | 843 | arylesterase | OXL21809.1 |
| NEXU01000075 | 18669 | 17341 | 1329 | MFS transporter | OXL21813.1 |
| NEXU01000075 | 19236 | 18991 | 246 | hypothetical protein | OXL21810.1 |
| NEXU01000075 | 20539 | 19394 | 1146 | thiamine biosynthesis protein | OXL21811.1 |
| NEXU01000075 | 21467 | 20667 | 801 | oxidoreductase | OXL21812.1 |
| NEXU01000076 | 1464 | 229 | 1236 | NADH:ubiquinone reductase (Na(+)-transporting) subunit F | OXL21705.1 |
| NEXU01000076 | 2299 | 1691 | 609 | NADH:ubiquinone reductase (Na(+)-transporting) subunit E |  |
| NEXU01000077 | 342 | <1 | N/D | NADH:ubiquinone reductase (Na(+)-transporting) subunit E | OXL21693.1 |
| NEXU01000077 | 1013 | 342 | 672 | NADH:ubiquinone reductase (Na(+)-transporting) subunit D | OXL21694.1 |
| NEXU01000077 | 1938 | 1015 | 924 | Na(+)-translocating NADH-quinone reductase subunit C | OXL21695.1 |
| NEXU01000077 | 3154 | 1919 | 1236 | NADH:ubiquinone reductase (Na(+)-transporting) subunit B | OXL21696.1 |
| NEXU01000077 | 4512 | 3160 | 1353 | NADH:ubiquinone reductase (Na(+)-transporting) subunit A | OXL21697.1 |
| NEXU01000077 | 5554 | 6297 | 744 | 1-(5-phosphoribosyl)-5-[(5- phosphoribosylamino)methylideneamino]imidazole-4-carboxamide isomerase | OXL21698.1 |
| NEXU01000077 | 6379 | 8094 | 1716 | mechanosensitive ion channel protein MscS | OXL21699.1 |
| NEXU01000077 | 8182 | 8478 | 297 | hypothetical protein | OXL21700.1 |
| NEXU01000077 | 10833 | 8566 | 2268 | ribonuclease R | OXL21701.1 |
| NEXU01000077 | 12210 | 10972 | 1239 | toxic anion resistance protein | OXL21702.1 |
| NEXU01000077 | 13227 | 12724 | 504 | translation initiation factor IF-3 | OXL21703.1 |
| NEXU01000077 | 15185 | 13251 | 1935 | threonine--tRNA ligase | OXL21704.1 |
| NEXU01000078 | 297 | 1052 | 756 | hypothetical protein | OXL21601.1 |
| NEXU01000078 | 1867 | 1121 | 747 | hypothetical protein | OXL21594.1 |
| NEXU01000078 | 2706 | 1960 | 747 | hypothetical protein | OXL21595.1 |
| NEXU01000078 | 3847 | 2942 | 906 | hypothetical protein | OXL21596.1 |
| NEXU01000078 | 4318 | 5517 | 1200 | MFS transporter | OXL21602.1 |
| NEXU01000078 | 5778 | 7448 | 1671 | long-chain fatty acid--CoA ligase | OXL21597.1 |
| NEXU01000078 | 7694 | 9766 | 2073 | propionate--CoA ligase | OXL21598.1 |
| NEXU01000078 | 10064 | 11548 | 1485 | methylmalonate-semialdehyde dehydrogenase (CoA acylating) | OXL21599.1 |
| NEXU01000078 | 11735 | >12397 | N/D | acyl-CoA dehydrogenase | OXL21600.1 |
| NEXU01000079 | <1 | 695 | N/D | acyl-CoA dehydrogenase | OXL21489.1 |
| NEXU01000079 | 777 | 1559 | 783 | enoyl-CoA hydratase | OXL21490.1 |
| NEXU01000079 | 1631 | 2893 | 1263 | crotonase | OXL21556.1 |
| NEXU01000079 | 3016 | 3993 | 978 | 3-hydroxyisobutyrate dehydrogenase | OXL21491.1 |
| NEXU01000079 | 4840 | 4082 | 759 | IclR family transcriptional regulator | OXL21492.1 |
| NEXU01000079 | 5486 | 6358 | 873 | hypothetical protein | OXL21493.1 |
| NEXU01000079 | 7230 | 6490 | 741 | hypothetical protein | OXL21557.1 |
| NEXU01000079 | 7937 | 7419 | 519 | peptidoglycan-associated lipoprotein | OXL21494.1 |
| NEXU01000079 | 8395 | 10983 | 2589 | aminopeptidase N | OXL21558.1 |
| NEXU01000079 | 12200 | 11118 | 1083 | 3-deoxy-7-phosphoheptulonate synthase | OXL21495.1 |
| NEXU01000079 | 12645 | 13967 | 1323 | lipid-A-disaccharide synthase | OXL21496.1 |
| NEXU01000079 | 14025 | 14888 | 864 | ribonuclease HII | OXL21497.1 |
| NEXU01000079 | 15713 | 15048 | 666 | hypothetical protein | OXL21498.1 |
| NEXU01000079 | 15803 | 17041 | 1239 | carboxynorspermidine decarboxylase | OXL21499.1 |
| NEXU01000079 | 17320 | 18591 | 1272 | saccharopine dehydrogenase | OXL21559.1 |
| NEXU01000079 | 18733 | 19377 | 645 | hypothetical protein | OXL21560.1 |
| NEXU01000079 | 19605 | 20471 | 867 | hypothetical protein | OXL21500.1 |
| NEXU01000079 | 20582 | 21001 | 420 | hypothetical protein | OXL21501.1 |
| NEXU01000079 | 21281 | 23560 | 2280 | Choline-glycine betaine transporter, BCCT family | OXL21502.1 |
| NEXU01000079 | 23757 | 24407 | 651 | hypothetical protein | OXL21503.1 |
| NEXU01000079 | 25042 | 24581 | 462 | hypothetical protein | OXL21504.1 |
| NEXU01000079 | 25522 | 25070 | 453 | hypothetical protein | OXL21505.1 |
| NEXU01000079 | 25647 | 26108 | 462 | protein-S-isoprenylcysteine methyltransferase | OXL21561.1 |
| NEXU01000079 | 26208 | 27011 | 804 | phenazine biosynthesis protein PhzF | OXL21506.1 |
| NEXU01000079 | 27018 | 27350 | 333 | hypothetical protein | OXL21562.1 |
| NEXU01000079 | 27958 | 28557 | 600 | 6-carboxytetrahydropterin synthase QueD | OXL21507.1 |
| NEXU01000079 | 28563 | 29114 | 552 | tRNA (adenosine(37)-N6)-threonylcarbamoyltransferase complex ATPase subunit type 1 TsaE | OXL21508.1 |
| NEXU01000079 | 29189 | 31042 | 1854 | ATPase | OXL21509.1 |
| NEXU01000079 | 31134 | 32345 | 1212 | tRNA (adenosine(37)-N6)-dimethylallyltransferase MiaA | OXL21510.1 |
| NEXU01000079 | 32814 | 33365 | 552 | RNA chaperone Hfq | OXL21511.1 |
| NEXU01000079 | 33636 | 34628 | 993 | D-arabinose 5-phosphate isomerase | OXL21512.1 |
| NEXU01000079 | 34667 | 35233 | 567 | HAD family hydrolase | OXL21513.1 |
| NEXU01000079 | 35230 | 35814 | 585 | LPS export ABC transporter periplasmic protein LptC | OXL21514.1 |
| NEXU01000079 | 35974 | 36543 | 570 | lipopolysaccharide transport periplasmic protein LptA | OXL21515.1 |
| NEXU01000079 | 36659 | 37438 | 780 | LPS export ABC transporter ATP-binding protein | OXL21516.1 |
| NEXU01000079 | 37687 | 39132 | 1446 | DNA repair protein RadA | OXL21517.1 |
| NEXU01000079 | 40082 | 39462 | 621 | septal ring lytic transglycosylase RlpA family lipoprotein | OXL21518.1 |
| NEXU01000079 | 42244 | 41102 | 1143 | rod shape-determining protein RodA | OXL21519.1 |
| NEXU01000079 | 42505 | 43467 | 963 | peptidase M15A | OXL21520.1 |
| NEXU01000079 | 45136 | 43562 | 1575 | peptidase M16 | OXL21521.1 |
| NEXU01000079 | 46755 | 45205 | 1551 | peptidase M16 | OXL21522.1 |
| NEXU01000079 | 47742 | 48839 | 1098 | signal recognition particle-docking protein FtsY | OXL21563.1 |
| NEXU01000079 | 48905 | 49477 | 573 | cysteine methyltransferase | OXL21523.1 |
| NEXU01000079 | 50316 | 49567 | 750 | YebC/PmpR family DNA-binding transcriptional regulator | OXL21524.1 |
| NEXU01000079 | 50659 | 51489 | 831 | ion transporter | OXL21525.1 |
| NEXU01000079 | 52828 | 51527 | 1302 | ABC transporter | OXL21526.1 |
| NEXU01000079 | 53529 | 52981 | 549 | type II 3-dehydroquinate dehydratase | OXL21527.1 |
| NEXU01000079 | 54068 | 54283 | 216 | cold-shock protein CspA | OXL21528.1 |
| NEXU01000079 | 54562 | 55716 | 1155 | ATP-dependent RNA helicase RhlB | OXL21529.1 |
| NEXU01000079 | 56670 | 55816 | 855 | hypothetical protein | OXL21530.1 |
| NEXU01000079 | 57063 | 56770 | 294 | cbb3-type cytochrome oxidase assembly protein CcoS | OXL21531.1 |
| NEXU01000079 | 57967 | 57194 | 774 | hypothetical protein | OXL21532.1 |
| NEXU01000079 | 59114 | 57957 | 1158 | UDP-N-acetylenolpyruvoylglucosamine reductase | OXL21533.1 |
| NEXU01000079 | 59723 | 59226 | 498 | protein-tyrosine-phosphatase | OXL21534.1 |
| NEXU01000079 | 60447 | 60037 | 411 | RNA-binding protein | OXL21535.1 |
| NEXU01000079 | 61070 | 60729 | 342 | hypothetical protein | OXL21536.1 |
| NEXU01000079 | 61367 | 61101 | 267 | hypothetical protein | OXL21537.1 |
| NEXU01000079 | 61636 | 61397 | 240 | hypothetical protein | OXL21564.1 |
| NEXU01000079 | 63473 | 61920 | 1554 | threonine ammonia-lyase, biosynthetic | OXL21538.1 |
| NEXU01000079 | 63797 | 64456 | 660 | ribose 5-phosphate isomerase A | OXL21539.1 |
| NEXU01000079 | 65491 | 64568 | 924 | ornithine carbamoyltransferase | OXL21540.1 |
| NEXU01000079 | 66801 | 65701 | 1101 | alanine racemase | OXL21541.1 |
| NEXU01000079 | 68318 | 66915 | 1404 | replicative DNA helicase | OXL21542.1 |
| NEXU01000079 | 68675 | 69784 | 1110 | 4-hydroxythreonine-4-phosphate dehydrogenase PdxA | OXL21565.1 |
| NEXU01000079 | 70377 | 69967 | 411 | hypothetical protein | OXL21543.1 |
| NEXU01000079 | 70903 | 71154 | 252 | hypothetical protein | OXL21544.1 |
| NEXU01000079 | 71316 | 71561 | 246 | hypothetical protein | OXL21545.1 |
| NEXU01000079 | 71801 | 72313 | 513 | transposase | OXL21546.1 |
| NEXU01000079 | 72819 | 73682 | 864 | 16S rRNA (adenine(1518)-N(6)/adenine(1519)-N(6))- dimethyltransferase | OXL21547.1 |
| NEXU01000079 | 73826 | 74668 | 843 | bis(5'-nucleosyl)-tetraphosphatase (symmetrical) | OXL21548.1 |
| NEXU01000079 | 77230 | 74750 | 2481 | recombinase | OXL21549.1 |
| NEXU01000079 | 79457 | 77673 | 1785 | acyl-CoA dehydrogenase | OXL21550.1 |
| NEXU01000079 | 81705 | 79912 | 1794 | acyl-CoA dehydrogenase | OXL21551.1 |
| NEXU01000079 | 83782 | 82058 | 1725 | GGDEF domain-containing protein | OXL21552.1 |
| NEXU01000079 | 84772 | 83876 | 897 | 16S rRNA (uracil(1498)-N(3))-methyltransferase | OXL21553.1 |
| NEXU01000079 | 86023 | 85175 | 849 | methylenetetrahydrofolate reductase [NAD(P)H] | OXL21554.1 |
| NEXU01000079 | 87461 | 86037 | 1425 | adenosylhomocysteinase | OXL21555.1 |
| NEXU01000080 | 338 | 1672 | 1335 | ubiquinone biosynthesis protein UbiH | OXL21447.1 |
| NEXU01000080 | 1912 | 3216 | 1305 | 2-octaprenyl-3-methyl-6-methoxy-1,4-benzoquinol hydroxylase | OXL21448.1 |
| NEXU01000080 | 3247 | 3903 | 657 | flagellar motor protein MotB | OXL21449.1 |
| NEXU01000080 | 3981 | 4679 | 699 | cell envelope biogenesis protein OmpA | OXL21450.1 |
| NEXU01000080 | 4691 | 5353 | 663 | flagellar motor protein MotB | OXL21451.1 |
| NEXU01000080 | 5426 | 6721 | 1296 | hypothetical protein | OXL21452.1 |
| NEXU01000080 | 6780 | 7409 | 630 | hypothetical protein | OXL21453.1 |
| NEXU01000080 | 7489 | 8178 | 690 | hypothetical protein | OXL21454.1 |
| NEXU01000080 | 9144 | 8233 | 912 | hypothetical protein | OXL21455.1 |
| NEXU01000080 | 9346 | 12126 | 2781 | hypothetical protein | OXL21456.1 |
| NEXU01000080 | 12152 | 13363 | 1212 | hypothetical protein | OXL21457.1 |
| NEXU01000080 | 13360 | 14214 | 855 | hypothetical protein | OXL21458.1 |
| NEXU01000080 | 15299 | 14388 | 912 | hypothetical protein | OXL21459.1 |
| NEXU01000080 | 17254 | 15377 | 1878 | L-aspartate oxidase | OXL21460.1 |
| NEXU01000080 | 18395 | 17256 | 1140 | quinolinate synthase | OXL21461.1 |
| NEXU01000080 | 18736 | 19812 | 1077 | hypothetical protein | OXL21462.1 |
| NEXU01000080 | 20134 | 20994 | 861 | nicotinate-nucleotide diphosphorylase (carboxylating) | OXL21463.1 |
| NEXU01000080 | 21268 | 21909 | 642 | hypothetical protein | OXL21464.1 |
| NEXU01000080 | 22269 | 23651 | 1383 | potassium transporter | OXL21465.1 |
| NEXU01000080 | >24436 | 23892 | N/D | amino acid transporter | OXL21466.1 |
| NEXU01000081 | 117 | <1 | N/D | amino acid transporter | OXL20510.1 |
| NEXU01000081 | 302 | 1042 | 741 | hypothetical protein | OXL20511.1 |
| NEXU01000081 | 1240 | 1899 | 660 | hypothetical protein | OXL20512.1 |
| NEXU01000081 | 2108 | 2665 | 558 | hypothetical protein | OXL20513.1 |
| NEXU01000081 | 3722 | 2790 | 933 | 23S rRNA pseudouridylate synthase B | OXL20514.1 |
| NEXU01000081 | 4152 | 5258 | 1107 | sugar ABC transporter substrate-binding protein | OXL20515.1 |
| NEXU01000081 | 5278 | 5709 | 432 | protein tyrosine phosphatase | OXL20516.1 |
| NEXU01000081 | 5779 | 8040 | 2262 | lipopolysaccharide biosynthesis protein | OXL20517.1 |
| NEXU01000081 | 8094 | 10103 | 2010 | capsule biosynthesis protein CapD | OXL20518.1 |
| NEXU01000081 | 10169 | 11446 | 1278 | Vi polysaccharide biosynthesis protein VipA/TviB | OXL20519.1 |
| NEXU01000081 | 11464 | 12408 | 945 | oxidoreductase | OXL20520.1 |
| NEXU01000081 | 12421 | 13005 | 585 | N-acetyltransferase | OXL20521.1 |
| NEXU01000081 | 13007 | 14086 | 1080 | aminotransferase DegT | OXL20522.1 |
| NEXU01000081 | 14083 | 15336 | 1254 | hypothetical protein | OXL20523.1 |
| NEXU01000081 | 15323 | 16753 | 1431 | polysaccharide biosynthesis protein | OXL20524.1 |
| NEXU01000081 | 17266 | 18474 | 1209 | hypothetical protein | OXL20525.1 |
| NEXU01000081 | 18736 | 19824 | 1089 | hypothetical protein | OXL20526.1 |
| NEXU01000081 | 19821 | 20933 | 1113 | group 1 glycosyl transferase | OXL20527.1 |
| NEXU01000081 | 20930 | 22000 | 1071 | UDP-N-acetylglucosamine 2-epimerase (non-hydrolyzing) | OXL20528.1 |
| NEXU01000081 | 22004 | 23242 | 1239 | glycosyltransferase WbuB | OXL20529.1 |
| NEXU01000081 | 23246 | 23839 | 594 | sugar transferase | OXL20530.1 |
| NEXU01000081 | 23859 | 24497 | 639 | acetyltransferase | OXL20531.1 |
| NEXU01000081 | 24607 | 25809 | 1203 | aminotransferase | OXL20532.1 |
| NEXU01000081 | 25904 | 27352 | 1449 | hypothetical protein | OXL20552.1 |
| NEXU01000081 | 27785 | 28858 | 1074 | sugar ABC transporter substrate-binding protein | OXL20533.1 |
| NEXU01000081 | 28959 | 31241 | 2283 | lipopolysaccharide biosynthesis protein | OXL20534.1 |
| NEXU01000081 | 31324 | 32601 | 1278 | Vi polysaccharide biosynthesis protein VipA/TviB | OXL20535.1 |
| NEXU01000081 | 32641 | 33678 | 1038 | LPS biosynthesis protein WbpP | OXL20536.1 |
| NEXU01000081 | 33690 | 35489 | 1800 | multidrug ABC transporter ATP-binding protein | OXL20537.1 |
| NEXU01000081 | 35512 | 36528 | 1017 | hypothetical protein | OXL20538.1 |
| NEXU01000081 | 36866 | 38203 | 1338 | aminopeptidase | OXL20539.1 |
| NEXU01000081 | 38560 | 39558 | 999 | UDP-N-acetylglucosamine 4,6-dehydratase (inverting) | OXL20540.1 |
| NEXU01000081 | 39559 | 40725 | 1167 | UDP-4-amino-4, 6-dideoxy-N-acetyl-beta-L-altrosamine transaminase | OXL20541.1 |
| NEXU01000081 | 40710 | 41606 | 897 | hypothetical protein | OXL20542.1 |
| NEXU01000081 | 41603 | 42502 | 900 | hypothetical protein | OXL20543.1 |
| NEXU01000081 | 42499 | 43254 | 756 | hypothetical protein | OXL20544.1 |
| NEXU01000081 | 43248 | 43952 | 705 | acylneuraminate cytidylyltransferase | OXL20545.1 |
| NEXU01000081 | 43949 | 45070 | 1122 | UDP-2,4-diacetamido-2,4, 6-trideoxy-beta-L-altropyranose hydrolase | OXL20546.1 |
| NEXU01000081 | 45087 | 45734 | 648 | UDP-glucuronic acid dehydrogenase | OXL20547.1 |
| NEXU01000081 | 45724 | 46785 | 1062 | pseudaminic acid synthase | OXL20548.1 |
| NEXU01000081 | 46969 | 47868 | 900 | hypothetical protein | OXL20549.1 |
| NEXU01000081 | 48013 | 49098 | 1086 | hypothetical protein | OXL20550.1 |
| NEXU01000081 | 49098 | 50978 | 1881 | asparagine synthase (glutamine-hydrolyzing) | OXL20551.1 |
| NEXU01000082 | 268 | 1221 | 954 | hypothetical protein | OXL20503.1 |
| NEXU01000082 | 1452 | 2684 | 1233 | hypothetical protein | OXL20504.1 |
| NEXU01000082 | 2681 | 3364 | 684 | hypothetical protein | OXL20505.1 |
| NEXU01000082 | 3361 | 4125 | 765 | hypothetical protein | OXL20506.1 |
| NEXU01000082 | 4136 | 6094 | 1959 | asparagine synthase (glutamine-hydrolyzing) | OXL20507.1 |
| NEXU01000082 | 6309 | 7490 | 1182 | hypothetical protein | OXL20508.1 |
| NEXU01000083 | 350 | 781 | 432 | nucleoside-diphosphate kinase | OXL20390.1 |
| NEXU01000083 | 1107 | 2336 | 1230 | 23S rRNA (adenine(2503)-C(2))-methyltransferase | OXL20391.1 |
| NEXU01000083 | 2548 | 3495 | 948 | type IV pilus biogenesis/stability protein PilW | OXL20392.1 |
| NEXU01000083 | 3492 | 4310 | 819 | DNA-binding protein | OXL20393.1 |
| NEXU01000083 | 4496 | 5611 | 1116 | 4-hydroxy-3-methylbut-2-en-1-yl diphosphate synthase | OXL20394.1 |
| NEXU01000083 | 5738 | 7051 | 1314 | histidine--tRNA ligase | OXL20395.1 |
| NEXU01000083 | 7211 | 8005 | 795 | hypothetical protein | OXL20396.1 |
| NEXU01000083 | 8190 | 9398 | 1209 | outer membrane protein assembly factor BamB | OXL20397.1 |
| NEXU01000083 | 9789 | 11210 | 1422 | ribosome biogenesis GTPase Der | OXL20398.1 |
| NEXU01000083 | 11418 | 13370 | 1953 | two-component sensor histidine kinase | OXL20399.1 |
| NEXU01000083 | 14667 | 13501 | 1167 | serine-type D-Ala-D-Ala carboxypeptidase | OXL20400.1 |
| NEXU01000083 | 15715 | 15281 | 435 | hypothetical protein | OXL20401.1 |
| NEXU01000083 | 16231 | 17043 | 813 | 5'/3'-nucleotidase SurE | OXL20402.1 |
| NEXU01000083 | 17231 | 18043 | 813 | peptigoglycan-binding protein LysM | OXL20403.1 |
| NEXU01000083 | 18167 | 19471 | 1305 | lytic transglycosylase | OXL20404.1 |
| NEXU01000083 | 20449 | 19601 | 849 | UDP-2,3-diacylglucosamine diphosphatase | OXL20405.1 |
| NEXU01000083 | 21000 | 20485 | 516 | cyclophilin | OXL20406.1 |
| NEXU01000083 | 21077 | 21268 | 192 | hypothetical protein | OXL20407.1 |
| NEXU01000083 | 21387 | 23126 | 1740 | glutamine--tRNA ligase | OXL20408.1 |
| NEXU01000083 | 23342 | 24607 | 1266 | tellurium resistance protein (TerF) | OXL20409.1 |
| NEXU01000083 | 24909 | 25547 | 639 | peroxiredoxin | OXL20410.1 |
| NEXU01000083 | 25796 | 26458 | 663 | glutathione S-transferase | OXL20411.1 |
| NEXU01000083 | 26786 | 27745 | 960 | hypothetical protein | OXL20437.1 |
| NEXU01000083 | 28444 | 27860 | 585 | crossover junction endodeoxyribonuclease RuvC | OXL20412.1 |
| NEXU01000083 | 28598 | 29227 | 630 | hypothetical protein | OXL20413.1 |
| NEXU01000083 | 29824 | 31482 | 1659 | phosphoglycerate mutase (2,3-diphosphoglycerate-independent) | OXL20414.1 |
| NEXU01000083 | 31823 | 33370 | 1548 | peptidase S41 | OXL20415.1 |
| NEXU01000083 | 35183 | 33393 | 1791 | sigma-54-dependent Fis family transcriptional regulator | OXL20416.1 |
| NEXU01000083 | 37153 | 35375 | 1779 | two-component sensor histidine kinase | OXL20417.1 |
| NEXU01000083 | 37831 | 37196 | 636 | two-component system response regulator UvrY | OXL20418.1 |
| NEXU01000083 | 38251 | 39336 | 1086 | peptidase S11 | OXL20419.1 |
| NEXU01000083 | 39418 | 40101 | 684 | thiamine phosphate synthase | OXL20420.1 |
| NEXU01000083 | 40476 | 40147 | 330 | conjugal transfer protein TraR | OXL20421.1 |
| NEXU01000083 | 40817 | 42031 | 1215 | phosphoglycerate kinase | OXL20422.1 |
| NEXU01000083 | 42291 | 42677 | 387 | hypothetical protein | OXL20423.1 |
| NEXU01000083 | 43118 | 44155 | 1038 | fructose-1,6-bisphosphate aldolase | OXL20424.1 |
| NEXU01000083 | 44241 | 44864 | 624 | Holliday junction branch migration protein RuvA | OXL20425.1 |
| NEXU01000083 | 45039 | 46019 | 981 | Holliday junction branch migration DNA helicase RuvB | OXL20438.1 |
| NEXU01000083 | 47550 | 46120 | 1431 | FAD-binding oxidoreductase | OXL20426.1 |
| NEXU01000083 | 49401 | 47740 | 1662 | 2-octaprenylphenol hydroxylase | OXL20427.1 |
| NEXU01000083 | 50274 | 49567 | 708 | hypothetical protein | OXL20428.1 |
| NEXU01000083 | 51558 | 50431 | 1128 | bifunctional demethylmenaquinone methyltransferase/2-methoxy-6-polyprenyl-1,4-benzoquinol methylase | OXL20429.1 |
| NEXU01000083 | 52360 | 51650 | 711 | hypothetical protein | OXL20430.1 |
| NEXU01000083 | 52668 | 54347 | 1680 | ATPase | OXL20431.1 |
| NEXU01000083 | 54746 | 54516 | 231 | hypothetical protein | OXL20432.1 |
| NEXU01000083 | 55013 | 56620 | 1608 | phosphate permease | OXL20433.1 |
| NEXU01000083 | 58378 | 56741 | 1638 | aminotransferase | OXL20434.1 |
| NEXU01000083 | 58807 | 59232 | 426 | peptide-methionine (R)-S-oxide reductase | OXL20435.1 |
| NEXU01000083 | 59380 | 59871 | 492 | glutathione peroxidase | OXL20436.1 |
| NEXU01000084 | 35 | 111 | 77 | tRNA-Asp |  |
| NEXU01000084 | 1268 | 735 | 534 | hypothetical protein | OXL20385.1 |
| NEXU01000084 | 1556 | 2416 | 861 | universal stress protein | OXL20386.1 |
| NEXU01000084 | 2480 | 2815 | 336 | GlpM family protein | OXL20387.1 |
| NEXU01000084 | 3984 | 3028 | 957 | acyltransferase | OXL20388.1 |
| NEXU01000084 | >5585 | 4431 | N/D | arsenical efflux pump membrane protein ArsB | OXL20389.1 |
| NEXU01000085 | 153 | <1 | N/D | hypothetical protein | OXL20333.1 |
| NEXU01000085 | 1492 | 353 | 1140 | hypothetical protein | OXL20316.1 |
| NEXU01000085 | 2047 | 1685 | 363 | transcriptional regulator | OXL20317.1 |
| NEXU01000085 | 2806 | 2288 | 519 | hypothetical protein | OXL20318.1 |
| NEXU01000085 | 3880 | 3089 | 792 | sterol desaturase | OXL20319.1 |
| NEXU01000085 | 4529 | 3870 | 660 | hypothetical protein | OXL20320.1 |
| NEXU01000085 | 5254 | 4592 | 663 | ABC transporter ATP-binding protein | OXL20321.1 |
| NEXU01000085 | 7171 | 5381 | 1791 | ABC transporter permease | OXL20322.1 |
| NEXU01000085 | 8466 | 7168 | 1299 | ABC transporter substrate-binding protein | OXL20323.1 |
| NEXU01000085 | 10783 | 8630 | 2154 | pyridine nucleotide-disulfide oxidoreductase | OXL20324.1 |
| NEXU01000085 | 11065 | 10875 | 191 | hypothetical protein |  |
| NEXU01000085 | 12054 | 11173 | 882 | transcriptional regulator CynR | OXL20325.1 |
| NEXU01000085 | 12229 | 12891 | 663 | carbonic anhydrase | OXL20326.1 |
| NEXU01000085 | 13051 | 13521 | 471 | cyanase | OXL20327.1 |
| NEXU01000085 | 13618 | 14829 | 1212 | MFS transporter | OXL20328.1 |
| NEXU01000085 | 15179 | 14898 | 282 | hypothetical protein | OXL20329.1 |
| NEXU01000085 | 15848 | 15183 | 666 | DNA-binding response regulator | OXL20330.1 |
| NEXU01000085 | 16564 | 15986 | 579 | peptidase | OXL20331.1 |
| NEXU01000085 | 17376 | 16747 | 630 | alkaline phosphatase | OXL20332.1 |
| NEXU01000086 | 1241 | 183 | 1059 | tRNA pseudouridine(55) synthase TruB | OXL20260.1 |
| NEXU01000086 | 1664 | 1260 | 405 | ribosome-binding factor A | OXL20261.1 |
| NEXU01000086 | 4686 | 1960 | 2727 | translation initiation factor IF-2 | OXL20262.1 |
| NEXU01000086 | 6322 | 4838 | 1485 | transcription termination/antitermination protein NusA | OXL20263.1 |
| NEXU01000086 | 6996 | 6496 | 501 | ribosome assembly cofactor RimP | OXL20264.1 |
| NEXU01000086 | 7490 | 7414 | 77 | tRNA-Met |  |
| NEXU01000086 | 7696 | 7612 | 85 | tRNA-Leu |  |
| NEXU01000086 | 8164 | 7856 | 309 | preprotein translocase subunit SecG | OXL20265.1 |
| NEXU01000086 | 9191 | 8382 | 810 | triose-phosphate isomerase | OXL20266.1 |
| NEXU01000086 | 9623 | 11332 | 1710 | type IV-A pilus assembly ATPase PilB | OXL20267.1 |
| NEXU01000086 | 11562 | 12785 | 1224 | type II secretion system protein F | OXL20268.1 |
| NEXU01000086 | 12811 | 13710 | 900 | prepilin peptidase | OXL20269.1 |
| NEXU01000086 | 13864 | 14556 | 693 | dephospho-CoA kinase | OXL20270.1 |
| NEXU01000086 | 15289 | 14519 | 771 | 23S rRNA (guanosine(2251)-2'-O)-methyltransferase RlmB | OXL20271.1 |
| NEXU01000086 | 15830 | 15495 | 336 | hypothetical protein | OXL20272.1 |
| NEXU01000086 | 16413 | 18092 | 1680 | DNA repair protein RecN | OXL20273.1 |
| NEXU01000086 | 18224 | 18787 | 564 | hypothetical protein | OXL20274.1 |
| NEXU01000086 | 18851 | 19405 | 555 | Holliday junction resolvase RuvX | OXL20275.1 |
| NEXU01000086 | 19483 | 20007 | 525 | hypothetical protein | OXL20276.1 |
| NEXU01000086 | 20064 | 20324 | 261 | sulfurtransferase TusA | OXL20277.1 |
| NEXU01000086 | 20377 | 21585 | 1209 | methylase | OXL20278.1 |
| NEXU01000086 | 21696 | 22634 | 939 | site-specific tyrosine recombinase XerD | OXL20279.1 |
| NEXU01000086 | 23262 | 22828 | 435 | hypothetical protein | OXL20280.1 |
| NEXU01000086 | 23647 | 24398 | 752 | IS5/IS1182 family transposase |  |
| NEXU01000086 | 25726 | 24668 | 1059 | hypothetical protein | OXL20281.1 |
| NEXU01000086 | 26095 | 26703 | 609 | methyltransferase | OXL20282.1 |
| NEXU01000086 | 28025 | 26799 | 1227 | LPS export ABC transporter permease LptG | OXL20283.1 |
| NEXU01000086 | 29317 | 28025 | 1293 | LPS export ABC transporter permease LptF | OXL20305.1 |
| NEXU01000086 | 29852 | 30451 | 600 | isochorismatase | OXL20284.1 |
| NEXU01000086 | 30687 | 32381 | 1695 | leucyl aminopeptidase | OXL20285.1 |
| NEXU01000086 | 32418 | 32918 | 501 | DNA polymerase III subunit chi | OXL20286.1 |
| NEXU01000086 | 33115 | 34317 | 1203 | serine/threonine transporter SstT | OXL20287.1 |
| NEXU01000086 | 34838 | 34527 | 312 | MerR family transcriptional regulator | OXL20288.1 |
| NEXU01000086 | 35930 | 34947 | 984 | DNA-binding protein | OXL20289.1 |
| NEXU01000086 | 38060 | 36039 | 2022 | hypothetical protein | OXL20290.1 |
| NEXU01000086 | 38689 | 38198 | 492 | hypothetical protein | OXL20291.1 |
| NEXU01000086 | 39213 | 39575 | 363 | septal ring lytic transglycosylase RlpA family lipoprotein | OXL20292.1 |
| NEXU01000086 | 41716 | 39698 | 2019 | ABC transporter ATP-binding protein | OXL20293.1 |
| NEXU01000086 | 42209 | 42577 | 369 | iron-sulfur cluster insertion protein ErpA | OXL20294.1 |
| NEXU01000086 | 42664 | 42963 | 300 | hypothetical protein |  |
| NEXU01000086 | 43295 | 44869 | 1575 | glutamate--cysteine ligase | OXL20295.1 |
| NEXU01000086 | 44924 | 45448 | 525 | disulfide bond formation protein B | OXL20296.1 |
| NEXU01000086 | 45678 | 46304 | 627 | copper homeostasis protein | OXL20297.1 |
| NEXU01000086 | 46645 | 48150 | 1506 | UDP-N-acetylmuramate:L-alanyl-gamma-D-glutamyl- meso-diaminopimelate ligase | OXL20298.1 |
| NEXU01000086 | 48487 | 48741 | 255 | GlsB/YeaQ/YmgE family stress response membrane protein | OXL20299.1 |
| NEXU01000086 | 48947 | 49492 | 546 | peptide deformylase | OXL20300.1 |
| NEXU01000086 | 49570 | 50745 | 1176 | ornithine decarboxylase | OXL20306.1 |
| NEXU01000086 | 51871 | 50960 | 912 | hypothetical protein | OXL20301.1 |
| NEXU01000086 | 52736 | 51891 | 846 | 4-hydroxy-tetrahydrodipicolinate reductase | OXL20302.1 |
| NEXU01000086 | 54042 | 52906 | 1137 | molecular chaperone DnaJ | OXL20303.1 |
| NEXU01000086 | 54503 | >55096 | N/D | hypothetical protein | OXL20304.1 |
| NEXU01000087 | 780 | 1 | 780 | hypothetical protein |  |
| NEXU01000087 | 1879 | 1175 | 705 | 16S rRNA pseudouridine(516) synthase | OXL20248.1 |
| NEXU01000087 | 2076 | 2672 | 597 | YigZ family protein | OXL20232.1 |
| NEXU01000087 | 2794 | 3840 | 1047 | alpha/beta hydrolase | OXL20233.1 |
| NEXU01000087 | 3890 | 4432 | 543 | thioeseterase | OXL20234.1 |
| NEXU01000087 | 6527 | 4713 | 1815 | hypothetical protein | OXL20235.1 |
| NEXU01000087 | 6777 | 7820 | 1044 | 2-nitropropane dioxygenase | OXL20236.1 |
| NEXU01000087 | 9005 | 8058 | 948 | alpha/beta hydrolase | OXL20237.1 |
| NEXU01000087 | 9266 | 10831 | 1566 | MATE family efflux transporter | OXL20238.1 |
| NEXU01000087 | 11820 | 11062 | 759 | aspartate/glutamate racemase | OXL20239.1 |
| NEXU01000087 | 12052 | 12306 | 255 | hypothetical protein | OXL20240.1 |
| NEXU01000087 | 12390 | 13568 | 1179 | hypothetical protein | OXL20241.1 |
| NEXU01000087 | 13561 | 14760 | 1200 | hypothetical protein | OXL20242.1 |
| NEXU01000087 | 14757 | 15380 | 624 | hypothetical protein | OXL20243.1 |
| NEXU01000087 | 15518 | 16495 | 978 | alcohol dehydrogenase | OXL20244.1 |
| NEXU01000087 | 16962 | 17942 | 981 | alcohol dehydrogenase | OXL20245.1 |
| NEXU01000087 | 18022 | 19041 | 1020 | oxidoreductase | OXL20246.1 |
| NEXU01000087 | 19504 | 20160 | 657 | hypothetical protein | OXL20247.1 |
| NEXU01000088 | 387 | 2048 | 1662 | energy-dependent translational throttle protein EttA | OXL20210.1 |
| NEXU01000088 | 5011 | 2201 | 2811 | glycerol-3-phosphate 1-O-acyltransferase | OXL20211.1 |
| NEXU01000088 | 5259 | 5762 | 504 | aminoacyl-tRNA deacylase | OXL20212.1 |
| NEXU01000088 | 5845 | 6219 | 375 | 5-carboxymethyl-2-hydroxymuconate isomerase | OXL20213.1 |
| NEXU01000088 | 6733 | 6242 | 492 | hypothetical protein | OXL20214.1 |
| NEXU01000088 | 8706 | 6886 | 1821 | acyl-CoA dehydrogenase | OXL20215.1 |
| NEXU01000088 | 9116 | 10255 | 1140 | FUSC family protein | OXL20216.1 |
| NEXU01000088 | 11348 | 10482 | 867 | shikimate dehydrogenase | OXL20217.1 |
| NEXU01000088 | 11773 | 12447 | 675 | hypothetical protein | OXL20218.1 |
| NEXU01000088 | 12451 | 13413 | 963 | aminodeoxychorismate lyase | OXL20219.1 |
| NEXU01000088 | 13769 | 13425 | 345 | hypothetical protein | OXL20220.1 |
| NEXU01000088 | 13939 | 15222 | 1284 | aminodeoxychorismate lyase | OXL20221.1 |
| NEXU01000088 | 15333 | 16010 | 678 | dTMP kinase | OXL20222.1 |
| NEXU01000088 | 16872 | 16147 | 726 | hypothetical protein | OXL20223.1 |
| NEXU01000089 | 1822 | <1 | N/D | sodium:proton antiporter | OXL19708.1 |
| NEXU01000089 | 2497 | 3951 | 1455 | serine protease | OXL19709.1 |
| NEXU01000089 | 4497 | 5402 | 906 | 4-hydroxy-tetrahydrodipicolinate synthase | OXL19710.1 |
| NEXU01000089 | 5506 | 5847 | 342 | hypothetical protein | OXL19711.1 |
| NEXU01000089 | 5984 | 6697 | 714 | phosphoribosylaminoimidazolesuccinocarboxamide synthase | OXL19712.1 |
| NEXU01000089 | 6978 | 7064 | 87 | replication initiation protein |  |
| NEXU01000089 | 7538 | 7122 | 417 | hypothetical protein | OXL19713.1 |
| NEXU01000089 | 8016 | 8663 | 648 | 3-methyladenine DNA glycosylase | OXL19714.1 |
| NEXU01000089 | 8719 | 9030 | 312 | NGG1p interacting factor NIF3 | OXL19715.1 |
| NEXU01000089 | 9501 | 9130 | 372 | hypothetical protein | OXL19716.1 |
| NEXU01000089 | 10079 | 9723 | 357 | hypothetical protein | OXL19717.1 |
| NEXU01000089 | 10946 | 10296 | 651 | hypothetical protein | OXL19718.1 |
| NEXU01000089 | 12050 | 11847 | 204 | hypothetical protein | OXL19719.1 |
| NEXU01000089 | 12616 | 13666 | 1051 | dehydrogenase |  |
| NEXU01000090 | 1 | 1209 | 1209 | dehydrogenase |  |
| NEXU01000090 | 2772 | 1441 | 1332 | amino acid dehydrogenase | OXL19666.1 |
| NEXU01000090 | 3576 | 2809 | 768 | folate-binding Fe/S cluster repair protein | OXL19667.1 |
| NEXU01000090 | 3883 | 4236 | 354 | alkylphosphonate utilization protein | OXL19668.1 |
| NEXU01000090 | 4347 | 5648 | 1302 | glutamate-1-semialdehyde-2,1-aminomutase | OXL19669.1 |
| NEXU01000090 | 6140 | 5736 | 405 | hypothetical protein | OXL19699.1 |
| NEXU01000090 | 6638 | 8164 | 1527 | exopolyphosphatase | OXL19670.1 |
| NEXU01000090 | 8408 | 9211 | 804 | acetyl-CoA carboxylase carboxyl transferase subunit alpha | OXL19671.1 |
| NEXU01000090 | 9404 | 11011 | 1608 | tRNA lysidine(34) synthetase TilS | OXL19672.1 |
| NEXU01000090 | 11192 | 12025 | 834 | pyrroline-5-carboxylate reductase | OXL19673.1 |
| NEXU01000090 | 12148 | 12717 | 570 | hypothetical protein | OXL19674.1 |
| NEXU01000090 | 13732 | 12938 | 795 | hydroxymethylpyrimidine/phosphomethylpyrimidine kinase | OXL19675.1 |
| NEXU01000090 | 13979 | 14068 | 90 | tRNA-Ser |  |
| NEXU01000090 | 14252 | 14328 | 77 | tRNA-Arg |  |
| NEXU01000090 | 14506 | 15153 | 648 | hypothetical protein | OXL19676.1 |
| NEXU01000090 | 16141 | 15167 | 975 | calcium/sodium antiporter | OXL19677.1 |
| NEXU01000090 | 16719 | 16339 | 381 | aspartate 1-decarboxylase | OXL19678.1 |
| NEXU01000090 | 17414 | 16833 | 582 | aminoacyl-tRNA hydrolase | OXL19679.1 |
| NEXU01000090 | 18293 | 17610 | 684 | 50S ribosomal protein L25/general stress protein Ctc | OXL19680.1 |
| NEXU01000090 | 19561 | 18614 | 948 | ribose-phosphate pyrophosphokinase | OXL19681.1 |
| NEXU01000090 | 19747 | 19672 | 76 | tRNA-Gln |  |
| NEXU01000090 | 20066 | 19991 | 76 | tRNA-Gln |  |
| NEXU01000090 | 20817 | 20239 | 579 | hypothetical protein | OXL19682.1 |
| NEXU01000090 | 21864 | 20896 | 969 | 4-(cytidine 5'-diphospho)-2-C-methyl-D-erythritol kinase | OXL19683.1 |
| NEXU01000090 | 22611 | 21970 | 642 | outer membrane lipoprotein LolB | OXL19684.1 |
| NEXU01000090 | 24836 | 22890 | 1947 | hypothetical protein | OXL19685.1 |
| NEXU01000090 | 25563 | 26978 | 1416 | glutamyl-tRNA reductase | OXL19686.1 |
| NEXU01000090 | 27514 | 28650 | 1137 | aminotransferase | OXL19687.1 |
| NEXU01000090 | 28845 | 29282 | 438 | YeeE/YedE family protein | OXL19688.1 |
| NEXU01000090 | 29367 | 29789 | 423 | hypothetical protein | OXL19700.1 |
| NEXU01000090 | 31574 | 29901 | 1674 | acyl-CoA synthetase | OXL19689.1 |
| NEXU01000090 | 31892 | 34027 | 2136 | NADPH-dependent 2,4-dienoyl-CoA reductase | OXL19690.1 |
| NEXU01000090 | 34135 | 34788 | 654 | hypothetical protein | OXL19691.1 |
| NEXU01000090 | 34900 | 36105 | 1206 | serine hydrolase | OXL19692.1 |
| NEXU01000090 | 36549 | 36133 | 417 | lactoylglutathione lyase | OXL19693.1 |
| NEXU01000090 | 37621 | 36617 | 1005 | lipoate--protein ligase | OXL19694.1 |
| NEXU01000090 | 37840 | 38490 | 651 | hypothetical protein | OXL19695.1 |
| NEXU01000090 | 40208 | 38610 | 1599 | cholesterol oxidase | OXL19696.1 |
| NEXU01000090 | 40718 | 40972 | 255 | hypothetical protein | OXL19697.1 |
| NEXU01000090 | 41511 | 41693 | 183 | hypothetical protein |  |
| NEXU01000090 | 41793 | 42689 | 897 | EamA family transporter | OXL19698.1 |
| NEXU01000091 | 390 | 124 | 267 | hypothetical protein | OXL19657.1 |
| NEXU01000091 | 1340 | 492 | 849 | YciK family oxidoreductase | OXL19662.1 |
| NEXU01000091 | 2154 | 1444 | 711 | phosphoglycolate phosphatase | OXL19658.1 |
| NEXU01000091 | 3036 | 2191 | 846 | bifunctional 3-demethylubiquinol 3-O-methyltransferase/2-polyprenyl-6-hydroxyphenol methylase | OXL19659.1 |
| NEXU01000091 | 3533 | 4153 | 621 | disulfide bond formation protein DsbA | OXL19660.1 |
| NEXU01000091 | 4800 | 4288 | 513 | hypothetical protein | OXL19661.1 |
| NEXU01000092 | 1140 | 190 | 951 | RNase adaptor protein RapZ | OXL19647.1 |
| NEXU01000092 | 2066 | 1182 | 885 | pantoate--beta-alanine ligase | OXL19648.1 |
| NEXU01000092 | 2918 | 2118 | 801 | 3-methyl-2-oxobutanoate hydroxymethyltransferase | OXL19649.1 |
| NEXU01000092 | 3565 | 2993 | 573 | 2-amino-4-hydroxy-6- hydroxymethyldihydropteridine diphosphokinase | OXL19650.1 |
| NEXU01000092 | 5810 | 3600 | 2211 | poly(A) polymerase | OXL19651.1 |
| NEXU01000092 | 6353 | 5976 | 378 | competence protein ComEA | OXL19652.1 |
| NEXU01000092 | 7203 | 6340 | 864 | nucleoside triphosphate pyrophosphohydrolase | OXL19653.1 |
| NEXU01000092 | 7359 | 8891 | 1533 | phosphomannomutase | OXL19654.1 |
| NEXU01000092 | 10467 | 9052 | 1416 | UDP-glucose 6-dehydrogenase | OXL19655.1 |
| NEXU01000092 | 10696 | 10505 | 192 | hypothetical protein |  |
| NEXU01000093 | 1678 | <1 | N/D | glucose-6-phosphate isomerase | OXL19625.1 |
| NEXU01000093 | 2566 | 1682 | 885 | UTP--glucose-1-phosphate uridylyltransferase | OXL19626.1 |
| NEXU01000093 | >2928 | 2621 | N/D | UDP-glucose 4-epimerase | OXL19627.1 |
| NEXU01000094 | 645 | <1 | N/D | UDP-glucose 4-epimerase GalE | OXL19620.1 |
| NEXU01000094 | 969 | 761 | 209 | succinate--CoA ligase subunit alpha |  |
| NEXU01000095 | 1006 | 131 | 876 | succinate--CoA ligase subunit alpha | OXL19594.1 |
| NEXU01000095 | 2312 | 1146 | 1167 | succinate--CoA ligase subunit beta | OXL19595.1 |
| NEXU01000095 | 3954 | 2503 | 1452 | dihydrolipoyl dehydrogenase | OXL19596.1 |
| NEXU01000095 | 5453 | 4215 | 1239 | dihydrolipoamide succinyltransferase | OXL19597.1 |
| NEXU01000095 | 8495 | 5610 | 2886 | 2-oxoglutarate dehydrogenase E1 component | OXL19598.1 |
| NEXU01000095 | 10199 | 9489 | 711 | succinate dehydrogenase iron-sulfur subunit | OXL19599.1 |
| NEXU01000095 | 12241 | 10391 | 1851 | succinate dehydrogenase flavoprotein subunit | OXL19600.1 |
| NEXU01000095 | 13055 | 12651 | 405 | succinate dehydrogenase, hydrophobic membrane anchor protein | OXL19611.1 |
| NEXU01000095 | 13444 | 13058 | 387 | succinate dehydrogenase, cytochrome b556 subunit | OXL19601.1 |
| NEXU01000095 | 14470 | 15750 | 1281 | citrate (Si)-synthase | OXL19602.1 |
| NEXU01000095 | 16820 | 15819 | 1002 | hypothetical protein | OXL19603.1 |
| NEXU01000095 | 17418 | 16885 | 534 | type IV secretion protein Rhs | OXL19604.1 |
| NEXU01000095 | 17722 | 19011 | 1290 | AAA family ATPase | OXL19605.1 |
| NEXU01000095 | 19344 | 20456 | 1113 | 3,4-dihydroxy-2-butanone-4-phosphate synthase | OXL19606.1 |
| NEXU01000095 | 21085 | 20588 | 498 | thioesterase | OXL19607.1 |
| NEXU01000095 | 22242 | 21304 | 939 | hypothetical protein | OXL19608.1 |
| NEXU01000095 | 23342 | 22338 | 1005 | uroporphyrinogen III synthase | OXL19609.1 |
| NEXU01000095 | 24501 | 23446 | 1056 | hydroxymethylbilane synthase | OXL19610.1 |
| NEXU01000096 | 1106 | 369 | 738 | DNA-binding response regulator | OXL19580.1 |
| NEXU01000096 | 2596 | 1445 | 1152 | histidine kinase | OXL19581.1 |
| NEXU01000096 | 2710 | 4083 | 1374 | argininosuccinate lyase | OXL19584.1 |
| NEXU01000096 | 4269 | 4559 | 291 | oxidative damage protection protein | OXL19582.1 |
| NEXU01000096 | 5024 | 6895 | 1872 | phosphomethylpyrimidine synthase ThiC | OXL19585.1 |
| NEXU01000096 | 7277 | >9548 | N/D | hypothetical protein | OXL19583.1 |
| NEXU01000097 | <1 | 1025 | N/D | hypothetical protein | OXL19215.1 |
| NEXU01000097 | 2019 | 1129 | 891 | fructose bisphosphate aldolase | OXL19216.1 |
| NEXU01000097 | 3084 | 2191 | 894 | hypothetical protein | OXL19217.1 |
| NEXU01000097 | 3444 | 5336 | 1893 | multidrug ABC transporter ATP-binding protein | OXL19218.1 |
| NEXU01000097 | 5385 | 6275 | 891 | alpha/beta hydrolase | OXL19219.1 |
| NEXU01000097 | 6285 | 6902 | 618 | alpha-ketoglutarate-dependent dioxygenase AlkB | OXL19220.1 |
| NEXU01000097 | 7931 | 6936 | 996 | NADPH:quinone reductase | OXL19221.1 |
| NEXU01000097 | 8759 | 8001 | 759 | hypothetical protein | OXL19227.1 |
| NEXU01000097 | 10137 | 9025 | 1113 | hypothetical protein | OXL19222.1 |
| NEXU01000097 | 10643 | 12838 | 2196 | oligopeptidase A | OXL19223.1 |
| NEXU01000097 | 12839 | 13096 | 258 | hypothetical protein | OXL19224.1 |
| NEXU01000097 | 13238 | 13771 | 534 | hypothetical protein | OXL19225.1 |
| NEXU01000097 | 13798 | 14361 | 564 | hypothetical protein | OXL19226.1 |
| NEXU01000097 | >16490 | 14475 | N/D | polyribonucleotide nucleotidyltransferase | OXL19228.1 |
| NEXU01000098 | 1087 | 821 | 267 | 30S ribosomal protein S15 | OXL19211.1 |
| NEXU01000098 | 1530 | 1345 | 186 | hypothetical protein | OXL19212.1 |
| NEXU01000098 | 2442 | 1918 | 525 | peptidase C56 | OXL19213.1 |
| NEXU01000099 | 830 | 60 | 771 | IclR family transcriptional regulator | OXL19204.1 |
| NEXU01000099 | 1000 | 1335 | 336 | transporter |  |
| NEXU01000099 | 1341 | 2072 | 732 | hypothetical protein |  |
| NEXU01000099 | 2748 | 5522 | 2775 | phosphoenolpyruvate carboxylase | OXL19205.1 |
| NEXU01000099 | 6752 | 5769 | 984 | oxidoreductase | OXL19206.1 |
| NEXU01000100 | 591 | 1736 | 1146 | hypothetical protein | OXL19169.1 |
| NEXU01000100 | 2472 | 1771 | 702 | hypothetical protein | OXL19194.1 |
| NEXU01000100 | 3202 | 2549 | 654 | cytochrome C oxidase Cbb3 | OXL19195.1 |
| NEXU01000100 | 4880 | 3279 | 1602 | flavin monoamine oxidase | OXL19170.1 |
| NEXU01000100 | 6354 | 4990 | 1365 | cytochrome C oxidase Cbb3 | OXL19171.1 |
| NEXU01000100 | 7069 | 6455 | 615 | polyisoprenoid-binding protein | OXL19172.1 |
| NEXU01000100 | 8152 | 7454 | 699 | hypothetical protein | OXL19173.1 |
| NEXU01000100 | 8935 | 8201 | 735 | phosphoesterase | OXL19174.1 |
| NEXU01000100 | 9425 | 8982 | 444 | diacylglycerol kinase | OXL19175.1 |
| NEXU01000100 | 10859 | 9552 | 1308 | two-component sensor histidine kinase | OXL19176.1 |
| NEXU01000100 | 11645 | 10959 | 687 | DNA-binding response regulator | OXL19177.1 |
| NEXU01000100 | 13388 | 11691 | 1698 | phosphoethanolamine transferase | OXL19178.1 |
| NEXU01000100 | 13542 | 14027 | 486 | hypothetical protein | OXL19179.1 |
| NEXU01000100 | 14288 | 15283 | 996 | phosphoesterase PA-phosphatase | OXL19180.1 |
| NEXU01000100 | 16069 | 15533 | 537 | hypothetical protein |  |
| NEXU01000100 | 16380 | 16063 | 318 | DNA polymerase III subunit epsilon |  |
| NEXU01000100 | 16411 | 17514 | 1104 | acyl-CoA dehydrogenase | OXL19181.1 |
| NEXU01000100 | 17911 | 18405 | 495 | glycosyl transferase |  |
| NEXU01000100 | 18507 | 19268 | 762 | hypothetical protein | OXL19196.1 |
| NEXU01000100 | 19306 | 19665 | 360 | hypothetical protein | OXL19182.1 |
| NEXU01000100 | 20030 | 20893 | 864 | hypothetical protein | OXL19183.1 |
| NEXU01000100 | 21895 | 20909 | 987 | hypothetical protein | OXL19184.1 |
| NEXU01000100 | 23196 | 21994 | 1203 | hypothetical protein | OXL19185.1 |
| NEXU01000100 | 24557 | 23199 | 1359 | hypothetical protein | OXL19186.1 |
| NEXU01000100 | 26351 | 24678 | 1674 | hypothetical protein | OXL19187.1 |
| NEXU01000100 | 27605 | 27000 | 606 | hypothetical protein |  |
| NEXU01000100 | 29265 | 27610 | 1656 | hypothetical protein | OXL19188.1 |
| NEXU01000100 | 29977 | 29318 | 660 | DNA-binding response regulator | OXL19189.1 |
| NEXU01000100 | 30733 | 30137 | 597 | hypothetical protein | OXL19190.1 |
| NEXU01000100 | 31063 | 31806 | 744 | hypothetical protein | OXL19197.1 |
| NEXU01000100 | 31977 | 34715 | 2739 | hypothetical protein | OXL19191.1 |
| NEXU01000100 | 34773 | 35465 | 693 | hypothetical protein | OXL19192.1 |
| NEXU01000100 | >36104 | 35776 | N/D | hypothetical protein | OXL19193.1 |
| NEXU01000101 | 613 | <1 | N/D | hypothetical protein | OXL19161.1 |
| NEXU01000101 | 1360 | 689 | 672 | hypothetical protein | OXL19162.1 |
| NEXU01000101 | 1531 | 2928 | 1398 | aspartate aminotransferase | OXL19163.1 |
| NEXU01000101 | 3289 | 2963 | 327 | HPP family protein |  |
| NEXU01000101 | 4362 | 3683 | 680 | hypothetical protein |  |
| NEXU01000101 | 4647 | 11066 | 6420 | hypothetical protein | OXL19164.1 |
| NEXU01000101 | 13180 | 11423 | 1758 | hypothetical protein | OXL19165.1 |
| NEXU01000102 | 428 | 90 | 339 | hypothetical protein | OXL19148.1 |
| NEXU01000102 | 901 | 2076 | 1176 | hypothetical protein | OXL19149.1 |
| NEXU01000102 | 2258 | 3181 | 924 | hypothetical protein | OXL19150.1 |
| NEXU01000102 | 3610 | 4200 | 591 | resolvase | OXL19151.1 |
| NEXU01000103 | 870 | <1 | N/D | hypothetical protein | OXL18949.1 |
| NEXU01000103 | 1709 | 3292 | 1584 | cell filamentation protein Fic | OXL18950.1 |
| NEXU01000103 | 3561 | 4648 | 1088 | IS3 family transposase |  |
| NEXU01000103 | 10072 | 4802 | 5271 | hypothetical protein | OXL18951.1 |
| NEXU01000103 | 11148 | 10960 | 189 | hypothetical protein | OXL18952.1 |
| NEXU01000103 | 11785 | 11347 | 439 | IS3 family transposase |  |
| NEXU01000103 | 11863 | 12611 | 749 | IS5/IS1182 family transposase |  |
| NEXU01000103 | 12969 | 12607 | 363 | IS3 family transposase |  |
| NEXU01000104 | 320 | 1 | 320 | IS3 family transposase |  |
| NEXU01000104 | 1178 | 444 | 735 | hypothetical protein | OXL18920.1 |
| NEXU01000104 | 1766 | 1936 | 171 | transposase |  |
| NEXU01000104 | 2673 | 2078 | 596 | resolvase |  |
| NEXU01000104 | 3088 | 3618 | 531 | ferrous iron transporter B | OXL18921.1 |
| NEXU01000104 | 3697 | 4422 | 726 | pilus assembly protein | OXL18922.1 |
| NEXU01000104 | 4493 | 7048 | 2556 | fimbrial protein | OXL18923.1 |
| NEXU01000104 | 7045 | 8061 | 1017 | fimbrial protein | OXL18924.1 |
| NEXU01000104 | 8616 | 8335 | 282 | hypothetical protein | OXL18925.1 |
| NEXU01000104 | 9102 | 8899 | 204 | hypothetical protein | OXL18926.1 |
| NEXU01000104 | 10732 | 9749 | 984 | LysR family transcriptional regulator | OXL18927.1 |
| NEXU01000104 | 10833 | 11921 | 1089 | tartrate dehydrogenase | OXL18928.1 |
| NEXU01000104 | 12096 | 13802 | 1707 | BCCT family transporter | OXL18929.1 |
| NEXU01000104 | 14083 | 15198 | 1116 | ring-hydroxylating oxygenase subunit alpha | OXL18947.1 |
| NEXU01000104 | 15273 | 16724 | 1452 | succinate-semialdehyde dehydrogenase (NADP(+)) | OXL18930.1 |
| NEXU01000104 | 16769 | 17728 | 960 | ferredoxin--NADP(+) reductase | OXL18931.1 |
| NEXU01000104 | 17849 | 19561 | 1713 | hypothetical protein | OXL18932.1 |
| NEXU01000104 | 19788 | 20798 | 1011 | NADPH:quinone reductase | OXL18933.1 |
| NEXU01000104 | 20925 | 22436 | 1512 | hypothetical protein | OXL18934.1 |
| NEXU01000104 | 23527 | 22700 | 828 | IclR family transcriptional regulator | OXL18935.1 |
| NEXU01000104 | 23675 | 24859 | 1185 | acyl-CoA dehydrogenase | OXL18936.1 |
| NEXU01000104 | 25035 | 26273 | 1239 | CoA transferase | OXL18937.1 |
| NEXU01000104 | 26389 | 26652 | 264 | hypothetical protein | OXL18938.1 |
| NEXU01000104 | 26857 | 27336 | 480 | hypothetical protein | OXL18939.1 |
| NEXU01000104 | 28089 | 27587 | 503 | IS3 family transposase |  |
| NEXU01000104 | 28243 | 28587 | 345 | QacE family quaternary ammonium compound efflux SMR transporter | OXL18940.1 |
| NEXU01000104 | 28591 | 29182 | 592 | TetR family transcriptional regulator |  |
| NEXU01000104 | 30384 | 29752 | 633 | hypothetical protein |  |
| NEXU01000104 | 32413 | 31169 | 1245 | hypothetical protein | OXL18941.1 |
| NEXU01000104 | 32499 | 32708 | 210 | transcriptional regulator | OXL18942.1 |
| NEXU01000104 | 33279 | 34193 | 915 | hypothetical protein | OXL18943.1 |
| NEXU01000104 | 34362 | 35000 | 639 | peptide transporter | OXL18944.1 |
| NEXU01000104 | 35004 | 35213 | 210 | hypothetical protein | OXL18945.1 |
| NEXU01000104 | 35449 | >36236 | N/D | IS256 family transposase | OXL18946.1 |
| NEXU01000105 | <1 | >266 | N/D | IS256 family transposase | OXL18914.1 |
| NEXU01000106 | 176 | 1042 | 867 | transposase | OXL18907.1 |
| NEXU01000106 | 1104 | 1640 | 537 | hypothetical protein | OXL18908.1 |
| NEXU01000106 | 1671 | 2081 | 411 | osmotically inducible protein OsmC | OXL18909.1 |
| NEXU01000106 | 2118 | 2582 | 465 | flavodoxin | OXL18910.1 |
| NEXU01000106 | 2583 | 3209 | 627 | methylase | OXL18911.1 |
| NEXU01000106 | >3520 | 3317 | N/D | dienelactone hydrolase | OXL18912.1 |
| NEXU01000107 | 508 | <1 | N/D | dienelactone hydrolase | OXL18769.1 |
| NEXU01000107 | 1062 | 3149 | 2088 | catalase | OXL18754.1 |
| NEXU01000107 | 3499 | 4281 | 783 | Zn-dependent hydrolase | OXL18755.1 |
| NEXU01000107 | 4532 | 4457 | 76 | tRNA-Lys |  |
| NEXU01000107 | 4765 | 4690 | 76 | tRNA-Lys |  |
| NEXU01000107 | 7155 | 5158 | 1998 | macrolide ABC transporter permease/ATP-binding protein MacB | OXL18756.1 |
| NEXU01000107 | 8630 | 7266 | 1365 | efflux transporter periplasmic adaptor subunit | OXL18757.1 |
| NEXU01000107 | 9946 | 8876 | 1071 | DNA polymerase III subunit delta | OXL18758.1 |
| NEXU01000107 | 10679 | 10020 | 660 | hypothetical protein | OXL18770.1 |
| NEXU01000107 | 13585 | 10793 | 2793 | leucine--tRNA ligase | OXL18759.1 |
| NEXU01000107 | 13745 | 14101 | 357 | arsenate reductase | OXL18760.1 |
| NEXU01000107 | 14206 | 14565 | 360 | hypothetical protein | OXL18761.1 |
| NEXU01000107 | 15577 | 14666 | 912 | acetylglutamate kinase | OXL18762.1 |
| NEXU01000107 | 17678 | 15822 | 1857 | phosphomannomutase | OXL18763.1 |
| NEXU01000107 | 18246 | 17782 | 465 | deoxyuridine 5'-triphosphate nucleotidohydrolase | OXL18764.1 |
| NEXU01000107 | 19741 | 18338 | 1404 | magnesium transporter | OXL18765.1 |
| NEXU01000107 | 21873 | 19840 | 2034 | ATP-dependent DNA helicase Rep | OXL18766.1 |
| NEXU01000107 | 22771 | 23397 | 627 | NADH-quinone oxidoreductase subunit A | OXL18767.1 |
| NEXU01000107 | 23586 | 24254 | 669 | NADH-quinone oxidoreductase subunit B | OXL18768.1 |
| NEXU01000108 | 120 | 1895 | 1776 | NADH-quinone oxidoreductase subunit C/D | OXL18740.1 |
| NEXU01000108 | 2052 | 2561 | 510 | NADH-quinone oxidoreductase subunit E | OXL18741.1 |
| NEXU01000108 | 2558 | 3982 | 1425 | NADH oxidoreductase (quinone) subunit F | OXL18742.1 |
| NEXU01000108 | 3993 | 7103 | 3111 | NADH-quinone oxidoreductase subunit G | OXL18743.1 |
| NEXU01000108 | 7105 | 8148 | 1044 | NADH-quinone oxidoreductase subunit H | OXL18744.1 |
| NEXU01000108 | 8204 | 8752 | 549 | NADH-quinone oxidoreductase subunit I | OXL18745.1 |
| NEXU01000108 | 8749 | 9495 | 747 | NADH-quinone oxidoreductase subunit J | OXL18746.1 |
| NEXU01000108 | 9594 | 9902 | 309 | NADH-quinone oxidoreductase subunit K | OXL18751.1 |
| NEXU01000108 | 9907 | 11781 | 1875 | NADH-quinone oxidoreductase subunit L | OXL18747.1 |
| NEXU01000108 | 11783 | 13408 | 1626 | NADH-quinone oxidoreductase subunit M | OXL18748.1 |
| NEXU01000108 | 13412 | 14890 | 1479 | NADH-quinone oxidoreductase subunit N | OXL18749.1 |
| NEXU01000108 | 15062 | 15904 | 843 | ferredoxin--NADP(+) reductase | OXL18750.1 |
| NEXU01000108 | 16731 | 16021 | 711 | hypothetical protein |  |
| NEXU01000109 | 285 | 1257 | 973 | energy transducer TonB |  |
| NEXU01000109 | 1411 | 2286 | 876 | mechanosensitive ion channel protein MscS | OXL18734.1 |
| NEXU01000109 | 2882 | 2283 | 600 | rRNA maturation RNase YbeY | OXL18735.1 |
| NEXU01000109 | 3114 | 3941 | 828 | hypothetical protein | OXL18736.1 |
| NEXU01000109 | 5149 | 4064 | 1086 | PhoH family protein | OXL18737.1 |
| NEXU01000109 | 5148 | 5327 | 180 | hypothetical protein | OXL18738.1 |
| NEXU01000110 | 1627 | 296 | 1332 | lipase | OXL18540.1 |
| NEXU01000110 | 1988 | 2698 | 711 | succinate dehydrogenase iron-sulfur subunit | OXL18541.1 |
| NEXU01000110 | 2710 | 4293 | 1584 | hypothetical protein | OXL18542.1 |
| NEXU01000110 | 5627 | 4323 | 1305 | FAD-dependent oxidoreductase | OXL18543.1 |
| NEXU01000110 | 7707 | 6067 | 1641 | long-chain fatty acid--CoA ligase | OXL18544.1 |
| NEXU01000110 | 9002 | 7851 | 1152 | acyl-CoA dehydrogenase | OXL18545.1 |
| NEXU01000110 | 9394 | 10494 | 1101 | lipase chaperone | OXL18546.1 |
| NEXU01000110 | 10722 | 11774 | 1053 | hypothetical protein | OXL18547.1 |
| NEXU01000110 | 13144 | 11924 | 1221 | hypothetical protein | OXL18548.1 |
| NEXU01000110 | 14949 | 13261 | 1689 | long-chain fatty acid--CoA ligase | OXL18549.1 |
| NEXU01000110 | 16165 | 15107 | 1059 | CoA transferase | OXL18550.1 |
| NEXU01000110 | 18329 | 16158 | 2172 | 3-hydroxyacyl-CoA dehydrogenase | OXL18551.1 |
| NEXU01000110 | 19627 | 18419 | 1209 | acetyl-CoA acetyltransferase | OXL18552.1 |
| NEXU01000110 | 19888 | 20628 | 741 | TetR family transcriptional regulator | OXL18553.1 |
| NEXU01000110 | 22761 | 21079 | 1683 | NAD-dependent malic enzyme | OXL18554.1 |
| NEXU01000110 | 23648 | 24823 | 1176 | serine--glyoxylate aminotransferase | OXL18555.1 |
| NEXU01000110 | 24888 | 25868 | 981 | serine dehydratase | OXL18556.1 |
| NEXU01000110 | 26002 | 27153 | 1152 | alanine racemase | OXL18557.1 |
| NEXU01000110 | 27299 | 28306 | 1008 | ornithine cyclodeaminase | OXL18558.1 |
| NEXU01000110 | 29428 | 28490 | 939 | AraC family transcriptional regulator | OXL18559.1 |
| NEXU01000110 | 29949 | 30209 | 261 | hypothetical protein | OXL18560.1 |
| NEXU01000110 | 30349 | 30882 | 534 | hypothetical protein | OXL18561.1 |
| NEXU01000110 | 32787 | 30997 | 1791 | K+/H+ antiporter | OXL18562.1 |
| NEXU01000110 | 34818 | 33154 | 1665 | indolepyruvate decarboxylase | OXL18563.1 |
| NEXU01000110 | 35081 | 35155 | 75 | tRNA-Gly |  |
| NEXU01000110 | 35481 | 35260 | 222 | hypothetical protein | OXL18564.1 |
| NEXU01000110 | 35809 | 35585 | 225 | transcriptional regulator | OXL18565.1 |
| NEXU01000110 | 36006 | 36221 | 216 | hypothetical protein | OXL18566.1 |
| NEXU01000110 | 36238 | 37482 | 1245 | site-specific integrase | OXL18567.1 |
| NEXU01000110 | 37479 | 37772 | 294 | hypothetical protein | OXL18568.1 |
| NEXU01000110 | 37759 | 38100 | 342 | hypothetical protein | OXL18569.1 |
| NEXU01000110 | 39199 | 38303 | 897 | hypothetical protein | OXL18570.1 |
| NEXU01000110 | 39736 | 40620 | 885 | hypothetical protein | OXL18571.1 |
| NEXU01000110 | 41510 | 40692 | 819 | hypothetical protein |  |
| NEXU01000110 | 42052 | 41627 | 426 | hypothetical protein | OXL18572.1 |
| NEXU01000110 | 42865 | 42086 | 780 | hypothetical protein | OXL18573.1 |
| NEXU01000110 | 44804 | 43149 | 1656 | hypothetical protein | OXL18574.1 |
| NEXU01000110 | 45794 | 46225 | 432 | hypothetical protein | OXL18575.1 |
| NEXU01000110 | 46276 | 46635 | 360 | hypothetical protein | OXL18576.1 |
| NEXU01000110 | 47167 | 46889 | 279 | peptidylprolyl isomerase | OXL18577.1 |
| NEXU01000110 | 48354 | 47368 | 987 | 23S rRNA pseudouridine synthase F | OXL18578.1 |
| NEXU01000110 | 49368 | 48577 | 792 | aspartate dehydrogenase | OXL18579.1 |
| NEXU01000110 | 49678 | 49962 | 285 | hypothetical protein | OXL18580.1 |
| NEXU01000111 | 155 | 2395 | 2241 | hypothetical protein | OXL18537.1 |
| NEXU01000112 | 590 | 514 | 77 | tRNA-Met |  |
| NEXU01000112 | 1476 | 673 | 804 | pteridine reductase | OXL18511.1 |
| NEXU01000112 | 2748 | 1546 | 1203 | tRNA nucleotidyltransferase | OXL18512.1 |
| NEXU01000112 | 5287 | 2951 | 2337 | NADP-dependent malic enzyme | OXL18513.1 |
| NEXU01000112 | 7104 | 5632 | 1473 | hypothetical protein | OXL18514.1 |
| NEXU01000112 | 8690 | 7287 | 1404 | fatty acid desaturase | OXL18515.1 |
| NEXU01000112 | 9644 | 8916 | 729 | glycerophosphodiester phosphodiesterase | OXL18516.1 |
| NEXU01000112 | 10028 | 10951 | 924 | hypothetical protein | OXL18517.1 |
| NEXU01000112 | 11673 | 11074 | 600 | ATP:cob(I)alamin adenosyltransferase | OXL18518.1 |
| NEXU01000112 | 12009 | 13469 | 1461 | cysteine--tRNA ligase | OXL18519.1 |
| NEXU01000112 | 13679 | 15685 | 2007 | mechanosensitive ion channel protein MscS | OXL18534.1 |
| NEXU01000112 | 16059 | 17531 | 1473 | IMP dehydrogenase | OXL18520.1 |
| NEXU01000112 | 17808 | 19175 | 1368 | phosphoglucosamine mutase | OXL18521.1 |
| NEXU01000112 | 19228 | 19878 | 651 | pyridoxamine 5'-phosphate oxidase | OXL18522.1 |
| NEXU01000112 | 21325 | 20048 | 1278 | hemolysin D | OXL18523.1 |
| NEXU01000112 | 23040 | 21424 | 1617 | MFS transporter | OXL18524.1 |
| NEXU01000112 | 23966 | 23337 | 630 | glutathione S-transferase | OXL18525.1 |
| NEXU01000112 | 24690 | 24013 | 678 | type 1 glutamine amidotransferase domain-containing protein | OXL18526.1 |
| NEXU01000112 | 25901 | 24852 | 1050 | NADP-dependent oxidoreductase | OXL18527.1 |
| NEXU01000112 | 27234 | 26014 | 1221 | NADH-dependent alcohol dehydrogenase | OXL18528.1 |
| NEXU01000112 | 28119 | 27481 | 639 | TetR family transcriptional regulator | OXL18529.1 |
| NEXU01000112 | 28486 | 29970 | 1485 | aldehyde dehydrogenase | OXL18530.1 |
| NEXU01000112 | 30830 | 30600 | 231 | hypothetical protein | OXL18531.1 |
| NEXU01000112 | 31109 | 32362 | 1254 | NnrS family protein | OXL18532.1 |
| NEXU01000112 | 32485 | 33069 | 585 | Rrf2 family transcriptional regulator | OXL18533.1 |
| NEXU01000113 | 214 | 1461 | 1248 | tRNA epoxyqueuosine(34) reductase QueG | OXL18360.1 |
| NEXU01000113 | 2100 | 1591 | 510 | peptidoglycan-binding protein LysM | OXL18361.1 |
| NEXU01000113 | 2700 | 2464 | 237 | acyl carrier protein | OXL18362.1 |
| NEXU01000113 | 3711 | 2983 | 729 | 3-oxoacyl-ACP reductase | OXL18363.1 |
| NEXU01000113 | 4676 | 3708 | 969 | [acyl-carrier-protein] S-malonyltransferase | OXL18364.1 |
| NEXU01000113 | 5291 | 5109 | 183 | 50S ribosomal protein L32 | OXL18365.1 |
| NEXU01000113 | 6218 | 5625 | 594 | hypothetical protein | OXL18366.1 |
| NEXU01000113 | 6401 | 7108 | 708 | hypothetical protein | OXL18367.1 |
| NEXU01000113 | 7428 | 8048 | 621 | threonylcarbamoyl-AMP synthase | OXL18368.1 |
| NEXU01000113 | 8487 | 8128 | 360 | 50S ribosomal protein L17 | OXL18369.1 |
| NEXU01000113 | 9514 | 8507 | 1008 | DNA-directed RNA polymerase subunit alpha | OXL18370.1 |
| NEXU01000113 | 10237 | 9596 | 642 | 30S ribosomal protein S4 | OXL18371.1 |
| NEXU01000113 | 10675 | 10286 | 390 | 30S ribosomal protein S11 | OXL18372.1 |
| NEXU01000113 | 11052 | 10696 | 357 | 30S ribosomal protein S13 | OXL18373.1 |
| NEXU01000113 | 11425 | 11309 | 117 | 50S ribosomal protein L36 | OXL18374.1 |
| NEXU01000113 | 12803 | 11457 | 1347 | preprotein translocase subunit SecY | OXL18375.1 |
| NEXU01000113 | 13248 | 12808 | 441 | 50S ribosomal protein L15 | OXL18376.1 |
| NEXU01000113 | 13427 | 13248 | 180 | 50S ribosomal protein L30 | OXL18377.1 |
| NEXU01000113 | 13963 | 13448 | 516 | 30S ribosomal protein S5 | OXL18378.1 |
| NEXU01000113 | 14316 | 13966 | 351 | 50S ribosomal protein L18 | OXL18379.1 |
| NEXU01000113 | 14861 | 14328 | 534 | 50S ribosomal protein L6 | OXL18380.1 |
| NEXU01000113 | 15435 | 15037 | 399 | 30S ribosomal protein S8 | OXL18381.1 |
| NEXU01000113 | 15752 | 15447 | 306 | 30S ribosomal protein S14 | OXL18382.1 |
| NEXU01000113 | 16301 | 15765 | 537 | 50S ribosomal protein L5 | OXL18383.1 |
| NEXU01000113 | 16640 | 16323 | 318 | 50S ribosomal protein L24 | OXL18384.1 |
| NEXU01000113 | 17017 | 16649 | 369 | 50S ribosomal protein L14 | OXL18385.1 |
| NEXU01000113 | 17472 | 17200 | 273 | 30S ribosomal protein S17 | OXL18386.1 |
| NEXU01000113 | 17666 | 17469 | 198 | 50S ribosomal protein L29 | OXL18387.1 |
| NEXU01000113 | 18079 | 17666 | 414 | 50S ribosomal protein L16 | OXL18388.1 |
| NEXU01000113 | 18811 | 18083 | 729 | 30S ribosomal protein S3 | OXL18389.1 |
| NEXU01000113 | 19144 | 18815 | 330 | 50S ribosomal protein L22 | OXL18390.1 |
| NEXU01000113 | 19430 | 19155 | 276 | 30S ribosomal protein S19 | OXL18391.1 |
| NEXU01000113 | 20270 | 19443 | 828 | 50S ribosomal protein L2 | OXL18392.1 |
| NEXU01000113 | 20632 | 20282 | 351 | 50S ribosomal protein L23 | OXL18393.1 |
| NEXU01000113 | 21231 | 20629 | 603 | 50S ribosomal protein L4 | OXL18394.1 |
| NEXU01000113 | 21884 | 21246 | 639 | 50S ribosomal protein L3 | OXL18395.1 |
| NEXU01000113 | 22243 | 21932 | 312 | 30S ribosomal protein S10 | OXL18396.1 |
| NEXU01000113 | 24293 | 22707 | 1587 | ribonuclease E/G | OXL18397.1 |
| NEXU01000113 | 25165 | 24461 | 705 | septum formation inhibitor Maf | OXL18398.1 |
| NEXU01000113 | 25787 | 25302 | 486 | rod shape-determining protein MreD | OXL18399.1 |
| NEXU01000113 | 26780 | 25920 | 861 | rod shape-determining protein MreC | OXL18400.1 |
| NEXU01000113 | 28164 | 27136 | 1029 | rod shape-determining protein | OXL18401.1 |
| NEXU01000113 | 28806 | 29120 | 315 | aspartyl/glutamyl-tRNA(Asn/Gln) amidotransferase subunit C | OXL18402.1 |
| NEXU01000113 | 29190 | 30686 | 1497 | aspartyl/glutamyl-tRNA amidotransferase subunit A | OXL18403.1 |
| NEXU01000113 | 30686 | >30922 | N/D | hypothetical protein | OXL18404.1 |
| NEXU01000114 | <1 | 1252 | N/D | glutaminyl-tRNA synthase (glutamine-hydrolyzing) subunit B | OXL18351.1 |
| NEXU01000114 | 1367 | 1443 | 77 | tRNA-Pro |  |
| NEXU01000114 | 1659 | 2456 | 798 | hypothetical protein | OXL18352.1 |
| NEXU01000115 | 40 | 1845 | 1806 | NAD(P)H-hydrate dehydratase | OXL18348.1 |
| NEXU01000115 | 2370 | 1870 | 501 | DUF4124 domain-containing protein | OXL18347.1 |
| NEXU01000115 | 3147 | 4725 | 1579 | acetolactate synthase, large subunit, biosynthetic type |  |
| NEXU01000116 | 7 | 524 | 518 | acetolactate synthase 3 large subunit |  |
| NEXU01000116 | 524 | 1021 | 498 | acetolactate synthase small subunit | OXL18228.1 |
| NEXU01000116 | 1174 | 2190 | 1017 | ketol-acid reductoisomerase | OXL18229.1 |
| NEXU01000116 | 2463 | 2888 | 426 | hypothetical protein | OXL18247.1 |
| NEXU01000116 | 3269 | 3481 | 213 | cold shock protein CspE | OXL18230.1 |
| NEXU01000116 | 4239 | 4988 | 750 | electron transfer flavoprotein subunit beta | OXL18231.1 |
| NEXU01000116 | 5120 | 6052 | 933 | electron transfer flavoprotein subunit alpha | OXL18232.1 |
| NEXU01000116 | 6946 | 6152 | 795 | RNA methyltransferase | OXL18233.1 |
| NEXU01000116 | 8031 | 7048 | 984 | fructose-bisphosphatase class I | OXL18234.1 |
| NEXU01000116 | 8435 | 9508 | 1074 | aspartate carbamoyltransferase | OXL18235.1 |
| NEXU01000116 | 9611 | 10792 | 1182 | aspartate carbamoyltransferase | OXL18236.1 |
| NEXU01000116 | 10725 | 11288 | 564 | hypothetical protein | OXL18237.1 |
| NEXU01000116 | 12733 | 11456 | 1278 | sodium:proton antiporter | OXL18238.1 |
| NEXU01000116 | 14531 | 12885 | 1647 | hypothetical protein | OXL18239.1 |
| NEXU01000116 | 17641 | 14825 | 2817 | glycosyl transferase | OXL18240.1 |
| NEXU01000116 | 19359 | 17806 | 1554 | hypothetical protein | OXL18241.1 |
| NEXU01000116 | 19693 | 20307 | 615 | NAD(P)H:quinone oxidoreductase, type IV | OXL18242.1 |
| NEXU01000116 | 20341 | 20805 | 465 | hypothetical protein | OXL18243.1 |
| NEXU01000116 | 21693 | 20923 | 771 | hypothetical protein | OXL18244.1 |
| NEXU01000116 | 22253 | 22543 | 291 | co-chaperone GroES | OXL18245.1 |
| NEXU01000116 | 22639 | >23767 | N/D | chaperonin GroL | OXL18246.1 |
| NEXU01000117 | 1 | 744 | 744 | molecular chaperone GroEL |  |
| NEXU01000118 | <1 | 389 | N/D | hypothetical protein | OXL18103.1 |
| NEXU01000118 | 973 | 416 | 558 | preprotein translocase subunit SecA | OXL18104.1 |
| NEXU01000118 | 1749 | 1024 | 726 | hydrolase | OXL18123.1 |
| NEXU01000118 | 1863 | 2888 | 1026 | hypothetical protein | OXL18105.1 |
| NEXU01000118 | 3183 | 5990 | 2808 | aconitate hydratase 1 | OXL18106.1 |
| NEXU01000118 | 6560 | 6102 | 459 | hypothetical protein | OXL18107.1 |
| NEXU01000118 | 6974 | 6624 | 351 | glutaredoxin | OXL18108.1 |
| NEXU01000118 | 7767 | 7114 | 654 | homoserine lactone transporter | OXL18109.1 |
| NEXU01000118 | 8077 | 8002 | 76 | tRNA-Glu |  |
| NEXU01000118 | 8311 | 8236 | 76 | tRNA-Ala |  |
| NEXU01000118 | 8799 | 8434 | 366 | hypothetical protein | OXL18110.1 |
| NEXU01000118 | 10494 | 8953 | 1542 | glutamate--tRNA ligase | OXL18111.1 |
| NEXU01000118 | 10749 | 10570 | 180 | hypothetical protein | OXL18112.1 |
| NEXU01000118 | 10764 | 11933 | 1170 | 16S rRNA (cytosine(1402)-N(4))-methyltransferase | OXL18113.1 |
| NEXU01000118 | 12184 | 12732 | 549 | cell division protein FtsL | OXL18114.1 |
| NEXU01000118 | 12729 | 14837 | 2109 | peptidoglycan synthetase | OXL18115.1 |
| NEXU01000118 | 14907 | 16646 | 1740 | UDP-N-acetylmuramoyl-L-alanyl-D-glutamate--2, 6-diaminopimelate ligase | OXL18116.1 |
| NEXU01000118 | 16738 | 18195 | 1458 | UDP-N-acetylmuramoyl-tripeptide--D-alanyl-D- alanine ligase | OXL18117.1 |
| NEXU01000118 | 18199 | 19317 | 1119 | phospho-N-acetylmuramoyl-pentapeptide- transferase | OXL18118.1 |
| NEXU01000118 | 19623 | 20585 | 963 | rRNA (guanine-N1)-methyltransferase | OXL18119.1 |
| NEXU01000118 | 22212 | 20773 | 1440 | cardiolipin synthase | OXL18120.1 |
| NEXU01000118 | 23132 | 22338 | 795 | hypothetical protein | OXL18124.1 |
| NEXU01000118 | 23675 | 24298 | 624 | hypothetical protein | OXL18121.1 |
| NEXU01000118 | 24482 | 25078 | 597 | hypothetical protein | OXL18125.1 |
| NEXU01000118 | 25138 | 28137 | 3000 | hypothetical protein | OXL18126.1 |
| NEXU01000118 | 28221 | 29009 | 789 | hypothetical protein | OXL18122.1 |
| NEXU01000119 | 15 | 110 | 96 | hypothetical protein |  |
| NEXU01000119 | 903 | 202 | 702 | molybdenum ABC transporter permease subunit | OXL18081.1 |
| NEXU01000119 | 1995 | 1096 | 900 | molybdate ABC transporter substrate-binding protein | OXL18082.1 |
| NEXU01000119 | 2648 | 2157 | 492 | molybdenum cofactor biosynthesis protein MoaE | OXL18083.1 |
| NEXU01000119 | 2992 | 2714 | 279 | molybdopterin synthase sulfur carrier subunit | OXL18084.1 |
| NEXU01000119 | 3589 | 3053 | 537 | cyclic pyranopterin monophosphate synthase MoaC | OXL18085.1 |
| NEXU01000119 | 4380 | 3616 | 765 | molybdenum cofactor guanylyltransferase | OXL18086.1 |
| NEXU01000119 | 4911 | 4381 | 531 | molybdenum cofactor biosynthesis protein B | OXL18087.1 |
| NEXU01000119 | 6082 | 4979 | 1104 | GTP 3',8-cyclase MoaA | OXL18088.1 |
| NEXU01000119 | 7643 | 6348 | 1296 | molybdopterin molybdenumtransferase MoeA | OXL18089.1 |
| NEXU01000119 | 8651 | 7752 | 900 | peptidylprolyl isomerase | OXL18090.1 |
| NEXU01000119 | 8655 | 8951 | 297 | hypothetical protein | OXL18091.1 |
| NEXU01000119 | 10353 | 9139 | 1215 | flavohemoprotein | OXL18092.1 |
| NEXU01000119 | 11319 | 10594 | 726 | respiratory nitrate reductase subunit gamma | OXL18093.1 |
| NEXU01000119 | 12188 | 11331 | 858 | nitrate reductase molybdenum cofactor assembly chaperone | OXL18094 |
| NEXU01000119 | 13728 | 12190 | 1539 | nitrate reductase subunit beta | OXL18095.1 |
| NEXU01000119 | 17624 | 13866 | 3759 | nitrate reductase subunit alpha | OXL18096.1 |
| NEXU01000119 | 19427 | 18048 | 1380 | nitrate/nitrite transporter | OXL18097.1 |
| NEXU01000119 | 19794 | 21836 | 2043 | histidine kinase | OXL18098.1 |
| NEXU01000119 | 21952 | 22611 | 660 | two-component system response regulator NarL | OXL18099.1 |
| NEXU01000119 | 24227 | 22746 | 1482 | MFS transporter | OXL18100.1 |
| NEXU01000119 | 24588 | 24989 | 402 | hypothetical protein | OXL18101.1 |
| NEXU01000119 | 25402 | 27675 | 2274 | nitric oxide reductase large subunit | OXL18102.1 |
| NEXU01000120 | 453 | 187 | 267 | Fe-S protein | OXL17985.1 |
| NEXU01000120 | 628 | 1284 | 657 | hypothetical protein | OXL17986.1 |
| NEXU01000120 | 1395 | 3281 | 1887 | DNA topoisomerase IV subunit B | OXL17987.1 |
| NEXU01000120 | 3426 | 4118 | 693 | glycerol acyltransferase | OXL17988.1 |
| NEXU01000120 | 4942 | 4277 | 666 | hypothetical protein | OXL17989.1 |
| NEXU01000120 | 5216 | 6628 | 1413 | hypothetical protein | OXL17990.1 |
| NEXU01000120 | 8552 | 6771 | 1782 | hypothetical protein | OXL17991.1 |
| NEXU01000120 | 8956 | 9687 | 732 | peptidylprolyl isomerase | OXL17992.1 |
| NEXU01000120 | 11231 | 9909 | 1323 | amino-acid N-acetyltransferase | OXL17993.1 |
| NEXU01000120 | 11636 | 12223 | 588 | hypothetical protein | OXL17994.1 |
| NEXU01000120 | 13477 | 12449 | 1029 | patatin | OXL17995.1 |
| NEXU01000120 | 13761 | 16148 | 2388 | ATP-dependent DNA helicase RecG | OXL17996.1 |
| NEXU01000120 | 16752 | 17846 | 1095 | hypothetical protein | OXL17997.1 |
| NEXU01000120 | 18369 | 19721 | 1353 | efflux transporter periplasmic adaptor subunit | OXL17998.1 |
| NEXU01000120 | 19718 | 22819 | 3102 | RND transporter | OXL17999.1 |
| NEXU01000121 | <1 | 587 | N/D | hypothetical protein | OXL17967.1 |
| NEXU01000121 | 588 | 1457 | 870 | hypothetical protein | OXL17968.1 |
| NEXU01000121 | 1485 | 2087 | 603 | hypothetical protein | OXL17969.1 |
| NEXU01000121 | 2266 | 2457 | 192 | hypothetical protein | OXL17970.1 |
| NEXU01000121 | 2521 | 3399 | 879 | NADPH-dependent 7-cyano-7-deazaguanine reductase QueF | OXL17971.1 |
| NEXU01000121 | 3484 | 3873 | 390 | hypothetical protein | OXL17972.1 |
| NEXU01000121 | 5667 | 3949 | 1719 | proline--tRNA ligase | OXL17973.1 |
| NEXU01000121 | 6274 | 7053 | 780 | hypothetical protein | OXL17974.1 |
| NEXU01000121 | 7223 | 7435 | 213 | hypothetical protein | OXL17975.1 |
| NEXU01000121 | 9273 | 7624 | 1650 | deoxyribodipyrimidine photolyase | OXL17976.1 |
| NEXU01000121 | 9487 | 10170 | 684 | N-(5'-phosphoribosyl)anthranilate isomerase | OXL17977.1 |
| NEXU01000121 | 10294 | 11568 | 1275 | tryptophan synthase subunit beta | OXL17978.1 |
| NEXU01000121 | 11696 | 12520 | 825 | tryptophan synthase subunit alpha | OXL17979.1 |
| NEXU01000121 | 12857 | 13732 | 876 | acetyl-CoA carboxylase, carboxyltransferase subunit beta | OXL17984.1 |
| NEXU01000121 | 13921 | 15303 | 1383 | bifunctional folylpolyglutamate synthase/dihydrofolate synthase | OXL17980.1 |
| NEXU01000121 | 15578 | 16792 | 1215 | peptigoglycan-binding protein LysM | OXL17981.1 |
| NEXU01000121 | 17104 | 17634 | 531 | hypothetical protein | OXL17982.1 |
| NEXU01000121 | 18616 | 17663 | 954 | hypothetical protein | OXL17983.1 |
| NEXU01000122 | 910 | <1 | N/D | hypothetical protein | OXL17894.1 |
| NEXU01000122 | 1838 | 1131 | 708 | hypothetical protein | OXL17895.1 |
| NEXU01000123 | 308 | <1 | N/D | hypothetical protein | OXL17877.1 |
| NEXU01000123 | 1236 | 328 | 909 | class II glutamine amidotransferase | OXL17878.1 |
| NEXU01000123 | 1497 | 1237 | 261 | hypothetical protein | OXL17879.1 |
| NEXU01000123 | 2810 | 1560 | 1251 | homoserine kinase | OXL17880.1 |
| NEXU01000123 | 3000 | 3782 | 783 | imidazole glycerol phosphate synthase subunit HisF | OXL17881.1 |
| NEXU01000123 | 3930 | 4451 | 522 | 6,7-dimethyl-8-ribityllumazine synthase | OXL17882.1 |
| NEXU01000123 | 4711 | 5865 | 1155 | nitrogen utilization protein B | OXL17883.1 |
| NEXU01000123 | 5868 | 6902 | 1035 | thiamine-phosphate kinase | OXL17884.1 |
| NEXU01000123 | 6906 | 7496 | 591 | phosphatidylglycerophosphatase A | OXL17885.1 |
| NEXU01000123 | 7669 | 9045 | 1377 | UDP-N-acetylglucosamine diphosphorylase/glucosamine-1-phosphate N-acetyltransferase | OXL17886.1 |
| NEXU01000123 | 9158 | 9520 | 363 | hypothetical protein | OXL17887.1 |
| NEXU01000123 | 9691 | 11535 | 1845 | glutamine--fructose-6-phosphate transaminase (isomerizing) | OXL17888.1 |
| NEXU01000123 | 12510 | 11677 | 834 | hypothetical protein | OXL17889.1 |
| NEXU01000123 | 13105 | 12590 | 516 | flavin reductase | OXL17890.1 |
| NEXU01000123 | 14226 | 13180 | 1047 | 5-methyltetrahydropteroyltriglutamate-- homocysteine methyltransferase | OXL17891.1 |
| NEXU01000123 | 15277 | 14270 | 1008 | hypothetical protein | OXL17892.1 |
| NEXU01000123 | 15527 | >15625 | N/D | DNA-binding protein | OXL17893.1 |
| NEXU01000124 | 324 | 1823 | 1500 | lytic transglycosylase | OXL17806.1 |
| NEXU01000124 | 2264 | 1965 | 300 | hypothetical protein | OXL17807.1 |
| NEXU01000124 | 3529 | 2495 | 1035 | DUF4062 domain-containing protein | OXL17808.1 |
| NEXU01000124 | 4718 | 3729 | 990 | glutamate racemase | OXL17809.1 |
| NEXU01000124 | 5600 | 4866 | 735 | hypothetical protein | OXL17810.1 |
| NEXU01000124 | 6215 | 7882 | 1668 | electron transfer flavoprotein-ubiquinone oxidoreductase | OXL17817.1 |
| NEXU01000124 | 9521 | 8025 | 1497 | sodium:alanine symporter family protein | OXL17811.1 |
| NEXU01000124 | 10559 | 10819 | 261 | hypothetical protein | OXL17812.1 |
| NEXU01000124 | 10824 | 12692 | 1869 | cation acetate symporter | OXL17813.1 |
| NEXU01000124 | 12964 | 13509 | 546 | hypothetical protein | OXL17814.1 |
| NEXU01000124 | 14196 | 13591 | 606 | rhomboid family intramembrane serine protease | OXL17815.1 |
| NEXU01000124 | 15285 | 14233 | 1053 | 5'-nucleotidase | OXL17816.1 |
| NEXU01000124 | 15916 | 15698 | 219 | hypothetical protein | OXL17818.1 |
| NEXU01000125 | 14 | 1324 | 1311 | hypothetical protein | OXL17805.1 |
| NEXU01000125 | 1470 | 2840 | 1371 | acyl-CoA desaturase | OXL17795.1 |
| NEXU01000125 | 3097 | 3390 | 294 | GNAT family N-acetyltransferase | OXL17796.1 |
| NEXU01000125 | 3690 | 3502 | 189 | hypothetical protein | OXL17797.1 |
| NEXU01000125 | 3689 | 3982 | 294 | 4a-hydroxytetrahydrobiopterin dehydratase | OXL17798.1 |
| NEXU01000125 | 4301 | 4146 | 156 | DUF1328 domain-containing protein | OXL17799.1 |
| NEXU01000125 | 4859 | 6154 | 1296 | saccharopine dehydrogenase | OXL17800.1 |
| NEXU01000125 | 6236 | 6700 | 465 | hypothetical protein | OXL17801.1 |
| NEXU01000125 | 7177 | 7668 | 492 | hypothetical protein | OXL17802.1 |
| NEXU01000125 | 10378 | 7838 | 2541 | RNA-binding transcriptional accessory protein | OXL17803.1 |
| NEXU01000125 | 10755 | 11519 | 765 | two-component system response regulator OmpR | OXL17804.1 |
| NEXU01000126 | 1004 | 57 | 948 | polyketide cyclase | OXL17768.1 |
| NEXU01000126 | 2124 | 1066 | 1059 | polyketide cyclase | OXL17769.1 |
| NEXU01000126 | 2795 | 2253 | 543 | polyketide cyclase | OXL17770.1 |
| NEXU01000126 | 3595 | 2891 | 705 | acyl transferase | OXL17771.1 |
| NEXU01000126 | >4163 | 3689 | N/D | hypothetical protein | OXL17772.1 |
| NEXU01000127 | 1281 | 313 | 969 | cupin | OXL17753.1 |
| NEXU01000127 | 2410 | 1352 | 1059 | LacI family transcriptional regulator | OXL17749.1 |
| NEXU01000127 | 3311 | 2538 | 774 | 3-oxoacyl-ACP reductase | OXL17750.1 |
| NEXU01000127 | 4691 | 3378 | 1314 | histidinol dehydrogenase | OXL17751.1 |
| NEXU01000127 | 5243 | 6259 | 1017 | polyketide cyclase | OXL17752.1 |
| NEXU01000128 | 550 | 86 | 465 | hypothetical protein | OXL17726.1 |
| NEXU01000128 | 2109 | 820 | 1290 | adenylosuccinate synthase | OXL17727.1 |
| NEXU01000128 | 3606 | 2299 | 1308 | ATP phosphoribosyltransferase regulatory subunit | OXL17728.1 |
| NEXU01000128 | 4125 | 4514 | 390 | hypothetical protein | OXL17729.1 |
| NEXU01000128 | 4578 | 5792 | 1215 | magnesium and cobalt transport protein CorA | OXL17730.1 |
| NEXU01000128 | 6392 | 5907 | 486 | phosphate starvation-inducible protein PhoH | OXL17734.1 |
| NEXU01000128 | 7348 | 6452 | 897 | bifunctional 5,10-methylene-tetrahydrofolate dehydrogenase/5,10-methylene-tetrahydrofolate cyclohydrolase | OXL17731.1 |
| NEXU01000128 | 7604 | 8354 | 751 | IS5/IS1182 family transposase |  |
| NEXU01000128 | 9087 | 8398 | 690 | sugar transferase | OXL17732.1 |
| NEXU01000128 | >9999 | 9274 | N/D | glycosyltransferase family 1 protein | OXL17733.1 |
| NEXU01000129 | 702 | 223 | 480 | hypothetical protein | OXL17512.1 |
| NEXU01000129 | 1987 | 1055 | 933 | CDP-diacylglycerol--serine O-phosphatidyltransferase | OXL17504.1 |
| NEXU01000129 | 2873 | 1998 | 876 | 23S rRNA (adenine(2030)-N(6))-methyltransferase RlmJ | OXL17505.1 |
| NEXU01000129 | 3014 | 3802 | 789 | oxygenase | OXL17506.1 |
| NEXU01000129 | 3860 | 5068 | 1209 | succinyl-diaminopimelate desuccinylase | OXL17507.1 |
| NEXU01000129 | 6390 | 5248 | 1143 | hypothetical protein | OXL17508.1 |
| NEXU01000129 | 6950 | 6417 | 534 | AraC family transcriptional regulator | OXL17509.1 |
| NEXU01000129 | 7261 | 7052 | 210 | Fe-S assembly protein IscX | OXL17510.1 |
| NEXU01000129 | 7947 | 7393 | 555 | hypothetical protein | OXL17511.1 |
| NEXU01000130 | 3610 | 3535 | 76 | tRNA-Ala |  |
| NEXU01000130 | 3698 | 3622 | 77 | tRNA-Ile |  |
| NEXU01000131 | 166 | 1233 | 1068 | hypothetical protein | OXL17500.1 |
| NEXU01000131 | 1677 | 2600 | 924 | hypothetical protein | OXL17501.1 |
| NEXU01000131 | 2654 | 3091 | 438 | magnesium transporter MgtC | OXL17502.1 |
| NEXU01000131 | 4008 | 4775 | 768 | DUF5020 domain-containing protein | OXL17503.1 |
| NEXU01000132 | 1516 | 119 | 1398 | NAD synthetase | OXL17497.1 |
| NEXU01000132 | 1890 | 1513 | 378 | NAD(P) transhydrogenase subunit alpha | OXL17498.1 |
| NEXU01000132 | >3068 | 1964 | N/D | NAD(P) transhydrogenase subunit alpha | OXL17499.1 |
| NEXU01000133 | <1 | 573 | N/D | hypothetical protein | OXL17494.1 |
| NEXU01000133 | 1423 | 623 | 801 | hypothetical protein | OXL17495.1 |
| NEXU01000134 | 79 | 531 | 453 | hypothetical protein | OXL17270.1 |
| NEXU01000134 | 770 | 1027 | 258 | hypothetical protein | OXL17271.1 |
| NEXU01000134 | 1235 | 1552 | 318 | hypothetical protein | OXL17272.1 |
| NEXU01000135 | 695 | <1 | N/D | hypothetical protein | OXL17268.1 |
| NEXU01000135 | 1367 | 708 | 660 | thiopurine S-methyltransferase | OXL17269.1 |
| NEXU01000136 | 825 | 31 | 795 | hypothetical protein | OXL17267.1 |
| NEXU01000137 | 514 | <1 | N/D | DNA-binding protein | OXL17157.1 |
| NEXU01000138 | 464 | 6 | 459 | transposase | OXL17154.1 |
| NEXU01000138 | 855 | 505 | 351 | transposase | OXL17155.1 |
| NEXU01000139 | 437 | <1 | N/D | IS630 family transposase | OXL17152.1 |
| NEXU01000139 | 828 | 472 | 357 | transposase | OXL17153.1 |
| NEXU01000140 | 57 | >844 | N/D | IS256 family transposase | OXL17067.1 |
| NEXU01000141 | 777 | <1 | N/D | hypothetical protein | OXL17065.1 |
| NEXU01000142 | 395 | <1 | N/D | transposase | OXL16972.1 |
| NEXU01000142 | >748 | 448 | N/D | transposase | OXL16971.1 |
| NEXU01000143 | 393 | <1 | N/D | hypothetical protein | OXL16968.1 |
| NEXU01000143 | 632 | 390 | 243 | hypothetical protein | OXL16969.1 |
| NEXU01000144 | >634 | 16 | N/D | IS5/IS1182 family transposase | OXL16967.1 |
| NEXU01000145 | <1 | 188 | N/D | hypothetical protein | OXL16889.1 |
| NEXU01000145 | 423 | 596 | 174 | LysR family transcriptional regulator |  |
| NEXU01000146 | 371 | <1 | N/D | hypothetical protein | OXL16888.1 |
| NEXU01000147 | 275 | >555 | N/D | hypothetical protein | OXL16887.1 |
| NEXU01000148 | >527 | <1 | N/D | elongation factor 4 | OXL16729.1 |
| NEXU01000149 | >518 | 97 | N/D | DNA-binding response regulator | OXL16666.1 |
| NEXU01000150 | 480 | 265 | 216 | hypothetical protein | OXL16665.1 |
| NEXU01000151 | >510 | 79 | N/D | elongation factor Tu | OXL16664.1 |
| NEXU01000152 | >496 | <1 | N/D | UDP-glucose 4-epimerase | OXL16598.1 |
| NEXU01000153 | >487 | <1 | N/D | DNA helicase UvrD | OXL15520.1 |
| NEXU01000154 | 11 | 453 | 443 | hypothetical protein |  |
| NEXU01000155 | 448 | 1 | 448 | adenosine deaminase |  |
| NEXU01000156 | 1 | 300 | 300 | DNA polymerase III subunit epsilon |  |
| NEXU01000157 | >447 | <1 | N/D | DNA topoisomerase IV subunit A | OXL15400.1 |
| NEXU01000158 | <1 | >437 | N/D | hypothetical protein | OXL15399.1 |
| NEXU01000159 | <1 | >433 | N/D | hypothetical protein | OXL15398.1 |
| NEXU01000160 | <1 | 327 | N/D | hypothetical protein | OXL15345.1 |
| NEXU01000161 | >409 | 192 | N/D | hypothetical protein | OXL15344.1 |
| NEXU01000162 | 405 | <1 | N/D | glycosyltransferase family 1 protein | OXL15343.1 |
| NEXU01000163 | 9 | 353 | 345 | uroporphyrinogen III synthase |  |
| NEXU01000164 | <1 | >400 | N/D | hypothetical protein | OXL15286.1 |
| NEXU01000165 | <1 | >387 | N/D | 1-deoxy-D-xylulose-5-phosphate synthase | OXL15285.1 |
| NEXU01000166 | 23 | 385 | 363 | hypothetical protein |  |
| NEXU01000168 | >377 | <1 | N/D | peptide ABC transporter permease | OXL14743.1 |
| NEXU01000169 | <1 | >375 | N/D | hypothetical protein | OXL14742.1 |
| NEXU01000170 | <1 | >365 | N/D | hypothetical protein | OXL14741.1 |
| NEXU01000171 | <1 | >342 | N/D | hypothetical protein | OXL14702.1 |
| NEXU01000172 | 305 | <1 | N/D | hypothetical protein | OXL14701.1 |
| NEXU01000173 | 5 | 336 | 332 | phosphotransferase family protein |  |
| NEXU01000175 | >334 | <1 | N/D | hypothetical protein | OXL14425.1 |
| NEXU01000176 | 289 | <1 | N/D | hypothetical protein | OXL14399.1 |
| NEXU01000178 | >313 | <1 | N/D | hypothetical protein | OXL14363.1 |
| NEXU01000179 | <1 | >312 | N/D | ATP-binding protein | OXL14323.1 |
| NEXU01000180 | 188 | <1 | N/D | hypothetical protein | OXL14293.1 |
| NEXU01000181 | 310 | 71 | 240 | hypothetical protein |  |
| NEXU01000182 | >306 | 156 | N/D | 30S ribosomal protein S30 | OXL14262.1 |
| NEXU01000185 | <1 | >302 | N/D | hypothetical protein | OXL14235.1 |
| NEXU01000186 | 197 | <1 | N/D | hypothetical protein | OXL14213.1 |
| NEXU01000188 | >289 | <1 | N/D | ABC transporter | OXL14188.1 |
| NEXU01000190 | <1 | 237 | N/D | hypothetical protein | OXL14158.1 |
| NEXU01000193 | 219 | <1 | N/D | hypothetical protein | OXL14143.1 |
| NEXU01000198 | 191 | <1 | N/D | hypothetical protein | OXL14082.1 |
| NEXU01000201 | 1 | 76 | 76 | tRNA-Val |  |
| NEXU01000201 | 132 | 208 | 77 | tRNA-Asp |  |
| NEXU01000202 | <1 | >253 | N/D | dienelactone hydrolase | OXL13792.1 |
| NEXU01000203 | >250 | 46 | N/D | hypothetical protein | OXL13791.1 |
| NEXU01000204 | <1 | >249 | N/D | hypothetical protein | OXL13770.1 |
| NEXU01000205 | >249 | <1 | N/D | hypothetical protein | OXL13769.1 |
| NEXU01000206 | >245 | <1 | N/D | NADP-dependent oxidoreductase | OXL13749.1 |
| NEXU01000207 | >243 | <1 | N/D | DNA recombination/repair protein RecA | OXL13597.1 |
| NEXU01000208 | <1 | >243 | N/D | hypothetical protein | OXL13596.1 |
| NEXU01000209 | <1 | >234 | N/D | MBL fold metallo-hydrolase | OXL13583.1 |
| NEXU01000212 | 230 | 12 | 219 | peptide ABC transporter permease |  |
| NEXU01000213 | 93 | >226 | N/D | IS5/IS1182 family transposase | OXL13325.1 |
| NEXU01000214 | >224 | <1 | N/D | hypothetical protein | OXL13311.1 |
| NEXU01000216 | 21 | >205 | N/D | hypothetical protein | OXL13206.1 |

^a^ N/D – gene length cannot be determined due to imprecise gene coordinates (start and/or stop codons of predicted genes are localized beyond contig)
