# Supplementary material for: Exploring the genome of Arctic Psychrobacter sp. DAB_AL32B and construction of novel Psychrobacter-specific cloning vectors of an increased carrying capacity
Source: Arch Microbiol. 2018 Nov 17;201(5):559–69. doi: 10.1007/s00203-018-1595-y (PMC6579772; doi:10.1007/s00203-018-1595-y)
Supplement: Supplementary file 2 — Supplementary material 2 (DOCX 50 KB) [file 203_2018_1595_MOESM2_ESM.docx]

**Exploring the genome of Arctic *Psychrobacter* sp. DAB_AL32B and construction of novel *Psychrobacter*-specific cloning vectors of an increased carrying capacity**

Anna Ciok^1^, Lukasz Dziewit^1,^ *

^1^ University of Warsaw, Faculty of Biology, Institute of Microbiology, Department of Bacterial Genetics, Miecznikowa 1, 02-096 Warsaw, Poland

* Corresponding author:

Lukasz Dziewit

University of Warsaw

Faculty of Biology

Institute of Microbiology

Department of Bacterial Genetics

Miecznikowa 1, 02-096 Warsaw, Poland

tel: 48 225541406; fax: 48 225541402;

e-mail: [ldziewit@biol.uw.edu.pl](mailto:ldziewit@biol.uw.edu.pl)

**Table S2.** Results of Taxoblast anaysis and their manual examination.

| Contig name | Contig length (bp) | Number of hits | Number of queries | %Tax:497 | %Tax:2 | Contig classifiaction after manual examination |
| --- | --- | --- | --- | --- | --- | --- |
| 00001 | 14402 | 10 | 15 | 100 | 100 | not contaminant |
| 00002 | 692 | 0 | 1 |  |  | no significant homology |
| 00003 | 41562 | 8 | 42 | 100 | 100 | not contaminant |
| 00004 | 31946 | 32 | 32 | 100 | 100 | not contaminant |
| 00005 | 10842 | 11 | 11 | 100 | 100 | not contaminant |
| 00006 | 62358 | 63 | 63 | 100 | 100 | not contaminant |
| 00007 | 25665 | 26 | 26 | 100 | 100 | not contaminant |
| 00008 | 50783 | 51 | 51 | 100 | 100 | not contaminant |
| 00009 | 5655 | 4 | 6 | 75 | 100 | not contaminant |
| 00010 | 44474 | 45 | 45 | 95 | 100 | not contaminant |
| 00011 | 20296 | 21 | 21 | 100 | 100 | not contaminant |
| 00012 | 1295 | 2 | 2 | 100 | 100 | not contaminant |
| 00013 | 3517 | 4 | 4 | 100 | 100 | not contaminant |
| 00014 | 39237 | 39 | 40 | 100 | 100 | not contaminant |
| 00015 | 27165 | 28 | 28 | 100 | 100 | not contaminant |
| 00016 | 4269 | 5 | 5 | 100 | 100 | not contaminant |
| 00017 | 193226 | 193 | 194 | 100 | 100 | not contaminant |
| 00018 | 73854 | 74 | 74 | 98 | 100 | not contaminant |
| 00019 | 13470 | 14 | 14 | 100 | 100 | not contaminant |
| 00020 | 55035 | 54 | 56 | 88 | 100 | not contaminant |
| 00021 | 36454 | 37 | 37 | 100 | 100 | not contaminant |
| 00022 | 12930 | 13 | 13 | 92 | 100 | not contaminant |
| 00023 | 594 | 1 | 1 | 100 | 100 | not contaminant |
| 00024 | 30578 | 31 | 31 | 100 | 100 | not contaminant |
| 00025 | 17425 | 18 | 18 | 94 | 100 | not contaminant |
| 00026 | 747 | 1 | 1 | 100 | 100 | not contaminant |
| 00027 | 47206 | 48 | 48 | 100 | 100 | not contaminant |
| 00028 | 22039 | 23 | 23 | 100 | 100 | not contaminant |
| 00029 | 6566 | 7 | 7 | 100 | 100 | not contaminant |
| 00030 | 39211 | 40 | 40 | 100 | 100 | not contaminant |
| 00031 | 21551 | 22 | 22 | 100 | 100 | not contaminant |
| 00032 | 18338 | 19 | 19 | 100 | 100 | not contaminant |
| 00033 | 23945 | 24 | 24 | 100 | 100 | not contaminant |
| 00034 | 3662 | 4 | 4 | 100 | 100 | not contaminant |
| 00035 | 14259 | 15 | 15 | 100 | 100 | not contaminant |
| 00036 | 27802 | 28 | 28 | 100 | 100 | not contaminant |
| 00037 | 8196 | 9 | 9 | 100 | 100 | not contaminant |
| 00038 | 44674 | 42 | 45 | 59 | 83 | not contaminant |
| 00039 | 1892 | 2 | 2 | 100 | 100 | not contaminant |
| 00040 | 2778 | 3 | 3 | 100 | 100 | not contaminant |
| 00041 | 2487 | 3 | 3 | 100 | 100 | not contaminant |
| 00042 | 25198 | 26 | 26 | 100 | 100 | not contaminant |
| 00043 | 13913 | 14 | 14 | 100 | 100 | not contaminant |
| 00044 | 3867 | 4 | 4 | 100 | 100 | not contaminant |
| 00045 | 41489 | 42 | 42 | 100 | 100 | not contaminant |
| 00046 | 40267 | 41 | 41 | 100 | 100 | not contaminant |
| 00047 | 7455 | 8 | 8 | 100 | 100 | not contaminant |
| 00048 | 47938 | 37 | 48 | 51 | 91 | not contaminant |
| 00049 | 60884 | 57 | 61 | 91 | 100 | not contaminant |
| 00050 | 18104 | 19 | 19 | 100 | 100 | not contaminant |
| 00051 | 21241 | 22 | 22 | 100 | 100 | not contaminant |
| 00052 | 98787 | 96 | 99 | 95 | 98 | not contaminant |
| 00053 | 2984 | 3 | 3 | 100 | 100 | not contaminant |
| 00054 | 27230 | 28 | 28 | 100 | 100 | not contaminant |
| 00055 | 1215 | 2 | 2 | 100 | 100 | not contaminant |
| 00056 | 13383 | 14 | 14 | 100 | 100 | not contaminant |
| 00057 | 55855 | 56 | 56 | 100 | 100 | not contaminant |
| 00058 | 9880 | 10 | 10 | 100 | 100 | not contaminant |
| 00059 | 55474 | 56 | 56 | 100 | 100 | not contaminant |
| 00060 | 19163 | 20 | 20 | 100 | 100 | not contaminant |
| 00061 | 9630 | 10 | 10 | 100 | 100 | not contaminant |
| 00062 | 5708 | 6 | 6 | 100 | 100 | not contaminant |
| 00063 | 53911 | 54 | 54 | 100 | 100 | not contaminant |
| 00064 | 32813 | 33 | 33 | 100 | 100 | not contaminant |
| 00065 | 42096 | 43 | 43 | 100 | 100 | not contaminant |
| 00066 | 41846 | 41 | 42 | 100 | 100 | not contaminant |
| 00067 | 45526 | 46 | 46 | 100 | 100 | not contaminant |
| 00068 | 26846 | 27 | 27 | 100 | 100 | not contaminant |
| 00069 | 6698 | 7 | 7 | 100 | 100 | not contaminant |
| 00070 | 7965 | 8 | 8 | 100 | 100 | not contaminant |
| 00071 | 43685 | 44 | 44 | 100 | 100 | not contaminant |
| 00072 | 103334 | 104 | 104 | 100 | 100 | not contaminant |
| 00073 | 14650 | 15 | 15 | 93 | 100 | not contaminant |
| 00074 | 48657 | 49 | 49 | 100 | 100 | not contaminant |
| 00075 | 21682 | 22 | 22 | 100 | 100 | not contaminant |
| 00076 | 2316 | 3 | 3 | 100 | 100 | not contaminant |
| 00077 | 15862 | 16 | 16 | 100 | 100 | not contaminant |
| 00078 | 12397 | 13 | 13 | 100 | 100 | not contaminant |
| 00079 | 87912 | 88 | 88 | 98 | 100 | not contaminant |
| 00080 | 24436 | 25 | 25 | 100 | 100 | not contaminant |
| 00081 | 50988 | 51 | 51 | 70 | 98 | not contaminant |
| 00082 | 7712 | 5 | 8 | 80 | 80 | not contaminant |
| 00083 | 59891 | 60 | 60 | 100 | 100 | not contaminant |
| 00084 | 5585 | 6 | 6 | 100 | 100 | not contaminant |
| 00085 | 17557 | 18 | 18 | 100 | 100 | not contaminant |
| 00086 | 55096 | 56 | 56 | 98 | 100 | not contaminant |
| 00087 | 20385 | 21 | 21 | 95 | 100 | not contaminant |
| 00088 | 16955 | 17 | 17 | 100 | 100 | not contaminant |
| 00089 | 13666 | 14 | 14 | 100 | 100 | not contaminant |
| 00090 | 42716 | 43 | 43 | 100 | 100 | not contaminant |
| 00091 | 4982 | 5 | 5 | 100 | 100 | not contaminant |
| 00092 | 10696 | 11 | 11 | 100 | 100 | not contaminant |
| 00093 | 2928 | 3 | 3 | 100 | 100 | not contaminant |
| 00094 | 969 | 1 | 1 | 100 | 100 | not contaminant |
| 00095 | 24539 | 25 | 25 | 100 | 100 | not contaminant |
| 00096 | 9548 | 10 | 10 | 80 | 100 | not contaminant |
| 00097 | 16490 | 17 | 17 | 100 | 100 | not contaminant |
| 00098 | 2595 | 3 | 3 | 100 | 100 | not contaminant |
| 00099 | 7093 | 8 | 8 | 100 | 100 | not contaminant |
| 00100 | 36104 | 37 | 37 | 100 | 100 | not contaminant |
| 00101 | 13323 | 5 | 14 | 100 | 100 | not contaminant |
| 00102 | 4671 | 4 | 5 | 75 | 100 | not contaminant |
| 00103 | 12969 | 8 | 13 | 100 | 100 | not contaminant |
| 00104 | 36236 | 37 | 37 | 62 | 97 | not contaminant |
| 00105 | 266 | 1 | 1 | 100 | 100 | not contaminant |
| 00106 | 3520 | 4 | 4 | 100 | 100 | not contaminant |
| 00107 | 24307 | 25 | 25 | 100 | 100 | not contaminant |
| 00108 | 16731 | 17 | 17 | 100 | 100 | not contaminant |
| 00109 | 5528 | 6 | 6 | 100 | 100 | not contaminant |
| 00110 | 50123 | 51 | 51 | 90 | 100 | not contaminant |
| 00111 | 2808 | 3 | 3 | 100 | 100 | not contaminant |
| 00112 | 33124 | 34 | 34 | 100 | 100 | not contaminant |
| 00113 | 30922 | 31 | 31 | 100 | 100 | not contaminant |
| 00114 | 2493 | 2 | 3 | 100 | 100 | not contaminant |
| 00115 | 4725 | 5 | 5 | 100 | 100 | not contaminant |
| 00116 | 23767 | 24 | 24 | 100 | 100 | not contaminant |
| 00117 | 1002 | 1 | 2 | 100 | 100 | not contaminant |
| 00118 | 29090 | 28 | 30 | 100 | 100 | not contaminant |
| 00119 | 28038 | 29 | 29 | 100 | 100 | not contaminant |
| 00120 | 22899 | 23 | 23 | 100 | 100 | not contaminant |
| 00121 | 19014 | 17 | 20 | 100 | 100 | not contaminant |
| 00122 | 1840 | 2 | 2 | 50 | 100 | not contaminant |
| 00123 | 15625 | 16 | 16 | 100 | 100 | not contaminant |
| 00124 | 16048 | 17 | 17 | 100 | 100 | not contaminant |
| 00125 | 11550 | 12 | 12 | 100 | 100 | not contaminant |
| 00126 | 4163 | 5 | 5 | 100 | 100 | not contaminant |
| 00127 | 6395 | 7 | 7 | 100 | 100 | not contaminant |
| 00128 | 9999 | 10 | 10 | 80 | 100 | not contaminant |
| 00129 | 7983 | 8 | 8 | 100 | 100 | not contaminant |
| 00130 | 5769 | 6 | 6 | 100 | 100 | not contaminant |
| 00131 | 4802 | 5 | 5 | 100 | 100 | not contaminant |
| 00132 | 3068 | 4 | 4 | 100 | 100 | not contaminant |
| 00133 | 1952 | 2 | 2 | 100 | 100 | not contaminant |
| 00134 | 1600 | 2 | 2 | 50 | 100 | contaminant |
| 00135 | 1497 | 2 | 2 | 100 | 100 | not contaminant |
| 00136 | 862 | 1 | 1 | 100 | 100 | not contaminant |
| 00137 | 558 | 1 | 1 | 100 | 100 | not contaminant |
| 00138 | 919 | 1 | 1 | 100 | 100 | not contaminant |
| 00139 | 845 | 1 | 1 | 100 | 100 | not contaminant |
| 00140 | 844 | 1 | 1 | 0 | 100 | not contaminant |
| 00141 | 824 | 1 | 1 | 100 | 100 | not contaminant |
| 00142 | 748 | 1 | 1 | 100 | 100 | not contaminant |
| 00143 | 717 | 1 | 1 | 0 | 100 | contaminant |
| 00144 | 634 | 1 | 1 | 100 | 100 | not contaminant |
| 00145 | 606 | 1 | 1 | 100 | 100 | not contaminant |
| 00146 | 576 | 1 | 1 | 0 | 100 | contaminant |
| 00147 | 555 | 1 | 1 | 100 | 100 | not contaminant |
| 00148 | 527 | 1 | 1 | 100 | 100 | not contaminant |
| 00149 | 518 | 1 | 1 | 100 | 100 | not contaminant |
| 00150 | 511 | 0 | 1 |  |  | no significant homology |
| 00151 | 510 | 1 | 1 | 100 | 100 | not contaminant |
| 00152 | 496 | 1 | 1 | 100 | 100 | not contaminant |
| 00153 | 487 | 1 | 1 | 100 | 100 | not contaminant |
| 00154 | 453 | 1 | 1 | 100 | 100 | not contaminant |
| 00155 | 448 | 1 | 1 | 100 | 100 | not contaminant |
| 00156 | 446 | 1 | 1 | 100 | 100 | not contaminant |
| 00157 | 447 | 1 | 1 | 100 | 100 | not contaminant |
| 00158 | 437 | 1 | 1 | 100 | 100 | not contaminant |
| 00159 | 433 | 1 | 1 | 100 | 100 | not contaminant |
| 00160 | 433 | 1 | 1 | 100 | 100 | not contaminant |
| 00161 | 409 | 1 | 1 | 100 | 100 | not contaminant |
| 00162 | 407 | 1 | 1 | 0 | 100 | not contaminant |
| 00163 | 401 | 1 | 1 | 100 | 100 | not contaminant |
| 00164 | 400 | 1 | 1 | 100 | 100 | not contaminant |
| 00165 | 387 | 1 | 1 | 100 | 100 | not contaminant |
| 00166 | 385 | 1 | 1 | 100 | 100 | not contaminant |
| 00167 | 380 | 1 | 1 | 100 | 100 | not contaminant |
| 00168 | 377 | 1 | 1 | 100 | 100 | not contaminant |
| 00169 | 375 | 1 | 1 | 100 | 100 | not contaminant |
| 00170 | 365 | 1 | 1 | 100 | 100 | not contaminant |
| 00171 | 342 | 1 | 1 | 100 | 100 | not contaminant |
| 00172 | 337 | 1 | 1 | 100 | 100 | not contaminant |
| 00173 | 336 | 1 | 1 | 100 | 100 | not contaminant |
| 00174 | 333 | 1 | 1 | 0 | 100 | contaminant |
| 00175 | 334 | 1 | 1 | 100 | 100 | not contaminant |
| 00176 | 322 | 1 | 1 | 100 | 100 | not contaminant |
| 00177 | 318 | 0 | 1 |  |  | no significant homology |
| 00178 | 313 | 1 | 1 | 100 | 100 | not contaminant |
| 00179 | 312 | 1 | 1 | 100 | 100 | not contaminant |
| 00180 | 306 | 1 | 1 | 100 | 100 | not contaminant |
| 00181 | 310 | 1 | 1 | 0 | 100 | contaminant |
| 00182 | 306 | 1 | 1 | 100 | 100 | not contaminant |
| 00183 | 302 | 1 | 1 | 100 | 100 | not contaminant |
| 00184 | 301 | 0 | 1 |  |  | no significant homology |
| 00185 | 302 | 1 | 1 | 100 | 100 | not contaminant |
| 00186 | 292 | 1 | 1 | 100 | 100 | not contaminant |
| 00187 | 292 | 1 | 1 | 100 | 100 | not contaminant |
| 00188 | 289 | 1 | 1 | 100 | 100 | not contaminant |
| 00189 | 285 | 1 | 1 | 100 | 100 | not contaminant |
| 00190 | 286 | 1 | 1 | 100 | 100 | not contaminant |
| 00191 | 279 | 1 | 1 | 100 | 100 | not contaminant |
| 00192 | 281 | 1 | 1 | 100 | 100 | not contaminant |
| 00193 | 278 | 0 | 1 |  |  | no significant homology |
| 00194 | 272 | 1 | 1 | 100 | 100 | not contaminant |
| 00195 | 266 | 1 | 1 | 0 | 0 | contaminant |
| 00196 | 263 | 1 | 1 | 0 | 100 | contaminant |
| 00197 | 263 | 1 | 1 | 0 | 100 | contaminant |
| 00198 | 262 | 1 | 1 | 100 | 100 | not contaminant |
| 00199 | 257 | 0 | 1 |  |  | no significant homology |
| 00200 | 260 | 1 | 1 | 100 | 100 | not contaminant |
| 00201 | 257 | 1 | 1 | 100 | 100 | not contaminant |
| 00202 | 253 | 1 | 1 | 100 | 100 | not contaminant |
| 00203 | 250 | 1 | 1 | 100 | 100 | not contaminant |
| 00204 | 249 | 1 | 1 | 100 | 100 | not contaminant |
| 00205 | 249 | 1 | 1 | 0 | 100 | not contaminant |
| 00206 | 245 | 1 | 1 | 100 | 100 | not contaminant |
| 00207 | 243 | 1 | 1 | 100 | 100 | not contaminant |
| 00208 | 243 | 1 | 1 | 100 | 100 | not contaminant |
| 00209 | 234 | 1 | 1 | 100 | 100 | not contaminant |
| 00210 | 230 | 1 | 1 | 0 | 100 | contaminant |
| 00211 | 233 | 1 | 1 | 100 | 100 | not contaminant |
| 00212 | 230 | 1 | 1 | 100 | 100 | not contaminant |
| 00213 | 226 | 1 | 1 | 100 | 100 | not contaminant |
| 00214 | 224 | 1 | 1 | 100 | 100 | not contaminant |
| 00215 | 223 | 1 | 1 | 100 | 100 | not contaminant |
| 00216 | 205 | 1 | 1 | 100 | 100 | not contaminant |
| 00217 | 200 | 1 | 1 | 100 | 100 | not contaminant |
| 00218 | 200 | 0 | 1 |  |  | no significant homology |
