# Supplementary material for: Exploring the genome of Arctic Psychrobacter sp. DAB_AL32B and construction of novel Psychrobacter-specific cloning vectors of an increased carrying capacity
Source: Arch Microbiol. 2018 Nov 17;201(5):559–69. doi: 10.1007/s00203-018-1595-y (PMC6579772; doi:10.1007/s00203-018-1595-y)
Supplement: Supplementary file 3 — Supplementary material 3 (DOCX 31 KB) [file 203_2018_1595_MOESM3_ESM.docx]

**Exploring the genome of Arctic *Psychrobacter* sp. DAB_AL32B and construction of novel *Psychrobacter*-specific cloning vectors of an increased carrying capacity**

Anna Ciok^1^, Lukasz Dziewit^1,^ *

^1^ University of Warsaw, Faculty of Biology, Institute of Microbiology, Department of Bacterial Genetics, Miecznikowa 1, 02-096 Warsaw, Poland

* Corresponding author:

Lukasz Dziewit

University of Warsaw

Faculty of Biology

Institute of Microbiology

Department of Bacterial Genetics

Miecznikowa 1, 02-096 Warsaw, Poland

tel: 48 225541406; fax: 48 225541402;

e-mail: [ldziewit@biol.uw.edu.pl](mailto:ldziewit@biol.uw.edu.pl)

**Table S3.** The average nucleotide identity (ANI) and alignment fraction values values between DAB_AL32B and 76 genomes of *Psychrobacter* (sorted in descending order).

| Strain | GenBank assembly accession number | ANI value | Alignment fraction |
| --- | --- | --- | --- |
| *Psychrobacter* sp. DAB_AL43B | GCA_900168255.1 | 95.672 | 0.854 |
| *Psychrobacter* *arcticus* 273-4 | GCA_000012305.1 | 82.923 | 0.505 |
| *Psychrobacter* *cryohalolentis* FDAARGOS_308 | GCA_002208765.2 | 81.920 | 0.633 |
| *Psychrobacter* *cryohalolentis* K5 | GCA_000013905.1 | 81.918 | 0.633 |
| *Psychrobacter* sp. G | GCA_000418305.1 | 81.809 | 0.634 |
| *Psychrobacter* sp. UBA2769 | GCA_002352555.1 | 80.918 | 0.633 |
| *Psychrobacter* sp. UBA5041 | GCA_002416155.1 | 80.621 | 0.455 |
| *Psychrobacter* *cibarius* W1 | GCA_900016235.2 | 80.101 | 0.593 |
| *Psychrobacter* sp. JCM 18903 | GCA_000586475.1 | 80.045 | 0.592 |
| *Psychrobacter* glacincola BNF20 | GCA_001411745.2 | 80.045 | 0.594 |
| *Psychrobacter* sp. P11F6 | GCA_001435295.1 | 80.043 | 0.591 |
| *Psychrobacter* sp. JCM 18902 | GCA_000586455.1 | 80.022 | 0.592 |
| *Psychrobacter* *immobilis* DSM 7229 | GCA_003148585.1 | 80.020 | 0.577 |
| *Psychrobacter* sp. Sarcosine-02u-2 | GCA_002836335.1 | 79.993 | 0.590 |
| *Psychrobacter* sp. UBA6766 | GCA_002453355.1 | 79.992 | 0.600 |
| *Psychrobacter* sp. SHUES1 | GCA_001652315.1 | 79.907 | 0.592 |
| *Psychrobacter* sp. UBA3483 | GCA_002377905.1 | 79.905 | 0.592 |
| *Psychrobacter* sp. UBA6291 | GCA_002439405.1 | 79.873 | 0.594 |
| *Psychrobacter* sp. TB67 | GCA_000511065.1 | 79.748 | 0.559 |
| *Psychrobacter* sp. AC24 | GCA_000511635.1 | 79.742 | 0.559 |
| *Psychrobacter* sp. UBA5136 | GCA_002414005.1 | 79.715 | 0.565 |
| *Psychrobacter* sp. TB47 | GCA_000511045.1 | 79.682 | 0.553 |
| *Psychrobacter* sp. Sarcosine-3u-12 | GCA_002836005.1 | 79.678 | 0.566 |
| *Psychrobacter* sp. UBA6739 | GCA_002453835.1 | 79.628 | 0.572 |
| *Psychrobacter* sp. UBA6730 | GCA_002454035.1 | 79.611 | 0.556 |
| *Psychrobacter* sp. UBA4193 | GCA_002380345.1 | 79.590 | 0.403 |
| *Psychrobacter* sp. UBA2071 | GCA_002332465.1 | 79.379 | 0.529 |
| *Psychrobacter* sp. PAMC 21119 | GCA_000247495.2 | 79.323 | 0.553 |
| *Psychrobacter* *aquaticus* CMS 56 | GCA_000471625.1 | 79.269 | 0.538 |
| *Psychrobacter* *fozii* CECT 5889 | GCA_003217155.1 | 79.024 | 0.535 |
| *Psychrobacter* sp. UBA3068 | GCA_002366815.1 | 78.959 | 0.531 |
| *Psychrobacter* sp. P11G5 | GCA_001593305.1 | 78.719 | 0.520 |
| *Psychrobacter* sp. P2G3 | GCA_001593285.1 | 78.602 | 0.514 |
| *Psychrobacter* sp. 4Dc | GCA_002836715.1 | 78.519 | 0.522 |
| *Psychrobacter* sp. JCM 18901 | GCA_000586435.1 | 78.421 | 0.526 |
| *Psychrobacter* sp. B29-1 | GCA_001742065.1 | 78.420 | 0.507 |
| *Psychrobacter* sp. Choline-02u-13 | GCA_002836235.1 | 78.399 | 0.517 |
| *Psychrobacter* sp. Choline-02u-9 | GCA_002836505.1 | 78.382 | 0.522 |
| *Psychrobacter* sp. Choline-3u-12 | GCA_002836165.1 | 78.373 | 0.520 |
| *Psychrobacter* sp. MES7-P7E | GCA_002863645.1 | 78.365 | 0.517 |
| *Psychrobacter* sp. 4Bb | GCA_002836735.1 | 78.348 | 0.529 |
| *Psychrobacter* *alimentarius* PAMC 27889 | GCA_001606025.1 | 78.269 | 0.525 |
| *Psychrobacter* sp. JB193 | GCA_002285555.1 | 78.227 | 0.532 |
| *Psychrobacter* sp. UBA3480 | GCA_002377945.1 | 78.217 | 0.519 |
| *Psychrobacter* sp. JCM 18900 | GCA_000586415.1 | 78.217 | 0.520 |
| *Psychrobacter* sp. P11G3 | GCA_001435845.1 | 78.216 | 0.530 |
| *Psychrobacter* *piscatorii* LQ58 | GCA_001444505.1 | 78.175 | 0.519 |
| *Psychrobacter* sp. C 20.9 | GCA_001921765.1 | 78.175 | 0.519 |
| *Psychrobacter* sp. JB385 | GCA_900163785.1 | 78.173 | 0.521 |
| *Psychrobacter* sp. AntiMn-1 | GCA_001854065.1 | 78.031 | 0.513 |
| *Psychrobacter* *pacificensis* DSM 23406 | GCA_900101915.1 | 77.990 | 0.514 |
| *Psychrobacter* sp. UBA2514 | GCA_002341255.1 | 77.973 | 0.484 |
| *Psychrobacter* sp. Marseille-P5312 | GCA_900291405.1 | 77.839 | 0.465 |
| *Psychrobacter* *urativorans* R10.10B | GCA_001298525.1 | 77.821 | 0.439 |
| *Psychrobacter* sp. Rd 27.2 | GCA_001921755.1 | 77.796 | 0.478 |
| *Psychrobacter* sp. L7 | GCA_002810365.1 | 77.777 | 0.481 |
| *Psychrobacter* sp. ENNN9_III | GCA_001462175.1 | 77.669 | 0.447 |
| *Psychrobacter* sp. Cmf 22.2 | GCA_001921745.1 | 77.590 | 0.484 |
| *Psychrobacter* sp. TB15 | GCA_000511655.1 | 76.404 | 0.392 |
| *Psychrobacter* sp. TB2 | GCA_000508345.1 | 76.394 | 0.391 |
| *Psychrobacter* sp. ARS82 | GCA_002686735.1 | 76.136 | 0.242 |
| *Psychrobacter* sp. FDAARGOS_221 | GCA_002313155.2 | 74.624 | 0.210 |
| *Psychrobacter* sp. YP14 | GCA_003209315.1 | 74.612 | 0.218 |
| *Psychrobacter* sp. UBA6684 | GCA_002454875.1 | 74.557 | 0.245 |
| *Psychrobacter* sp. PRwf-1 | GCA_000016885.1 | 74.525 | 0.222 |
| *Psychrobacter* sp. 1501(2011) | GCA_000213615.1 | 74.401 | 0.215 |
| *Psychrobacter* *pasteurii* CIP110853 | GCA_900162815.1 | 74.360 | 0.227 |
| *Psychrobacter* *lutiphocae* DSM 21542 | GCA_000382145.1 | 74.312 | 0.198 |
| *Psychrobacter* *piechaudii* CIP110854 | GCA_900162825.1 | 74.300 | 0.212 |
| *Psychrobacter* sp. UBA3962 | GCA_002385225.1 | 74.098 | 0.214 |
| *Psychrobacter* *phenylpyruvicus* NBRC 102152 | GCA_001591185.1 | 74.096 | 0.215 |
| *Psychrobacter* *phenylpyruvicus* DSM 7000 | GCA_000685805.1 | 74.005 | 0.214 |
| *Psychrobacter* sp. UBA5114 | GCA_002415515.1 | 73.993 | 0.136 |
| *Psychrobacter* sp. UBA2578 | GCA_002340175.1 | 73.949 | 0.194 |
| *Psychrobacter* sp. 310(2012) | GCA_000690075.1 | 73.279 | 0.015 |
| *Psychrobacter* *immobilis* R988 | GCA_900203135.1 | 69.262 | 0.019 |
